# Community evaluation of glycoproteomics informatics solutions reveals high-performance search strategies for serum glycopeptide analysis

**DOI:** 10.1038/s41592-021-01309-x

**Published:** 2021-11-01

**Authors:** Rebeca Kawahara, Anastasia Chernykh, Kathirvel Alagesan, Marshall Bern, Weiqian Cao, Robert J. Chalkley, Kai Cheng, Matthew S. Choo, Nathan Edwards, Radoslav Goldman, Marcus Hoffmann, Yingwei Hu, Yifan Huang, Jin Young Kim, Doron Kletter, Benoit Liquet, Mingqi Liu, Yehia Mechref, Bo Meng, Sriram Neelamegham, Terry Nguyen-Khuong, Jonas Nilsson, Adam Pap, Gun Wook Park, Benjamin L. Parker, Cassandra L. Pegg, Josef M. Penninger, Toan K. Phung, Markus Pioch, Erdmann Rapp, Enes Sakalli, Miloslav Sanda, Benjamin L. Schulz, Nichollas E. Scott, Georgy Sofronov, Johannes Stadlmann, Sergey Y. Vakhrushev, Christina M. Woo, Hung-Yi Wu, Pengyuan Yang, Wantao Ying, Hui Zhang, Yong Zhang, Jingfu Zhao, Joseph Zaia, Stuart M. Haslam, Giuseppe Palmisano, Jong Shin Yoo, Göran Larson, Kai-Hooi Khoo, Katalin F. Medzihradszky, Daniel Kolarich, Nicolle H. Packer, Morten Thaysen-Andersen

**Affiliations:** 1 Department of Molecular Sciences, Macquarie University, Sydney, NSW Australia; 2 Institute for Glycomics, Griffith University Gold Coast Campus, Southport, QLD Australia; 3 Protein Metrics Inc., Cupertino, CA USA; 4 Institutes of Biomedical Sciences, and the NHC Key Laboratory of Glycoconjugates Research, Fudan University, Shanghai, China; 5 UCSF, School of Pharmacy, Department of Pharmaceutical Chemistry, San Francisco, CA USA; 6 State University of New York, Buffalo, NY USA; 7 Analytics Group, Bioprocessing Technology Institute, Agency for Science, Technology and Research, Singapore, Singapore; 8 Clinical and Translational Glycoscience Research Center (CTGRC), Georgetown University, Washington, DC USA; 9 Department of Biochemistry and Molecular & Cellular Biology, Georgetown University, Washington, DC USA; 10 Department of Oncology, Georgetown University, Washington, DC USA; 11 Max Planck Institute for Dynamics of Complex Technical Systems, Bioprocess Engineering, Magdeburg, Germany; 12 Department of Pathology, The Johns Hopkins University, Baltimore, MD USA; 13 Department of Chemistry and Biochemistry, Texas Tech University, Lubbock, TX USA; 14 Research Center of Bioconvergence Analysis, Korea Basic Science Institute, Daejeon, Republic of Korea; 15 Department of Mathematics and Statistics, Macquarie University, Sydney, NSW Australia; 16 CNRS, Laboratoire de Mathématiques et de leurs Applications de PAU, E2S-UPPA, Pau, France; 17 State Key Laboratory of Proteomics, Beijing Institute of Lifeomics, Beijing Proteome Research Center, National Center for Protein Sciences (Beijing), Beijing, China; 18 Proteomics Core Facility, Sahlgrenska academy, University of Gothenburg, Gothenburg, Sweden; 19 BRC, Laboratory of Proteomics Research, Szeged, Hungary; 20 Doctoral School in Biology, Faculty of Science and Informatics, University of Szeged, Szeged, Hungary; 21 Department of Anatomy and Physiology, University of Melbourne, Melbourne, VIC Australia; 22 School of Chemistry and Molecular Biosciences, University of Queensland, Queensland, QLD Australia; 23 IMBA, Institute of Molecular Biotechnology of the Austrian Academy of Sciences, Vienna, Austria; 24 Department of Medical Genetics, Life Sciences Institute, University of British Columbia, Vancouver, BC Canada; 25 glyXera GmbH, Magdeburg, Germany; 26 Deparment of Microbiology and Immunology, University of Melbourne, Melbourne, VIC Australia; 27 Copenhagen Center for Glycomics, Department of Cellular and Molecular Medicine, University of Copenhagen, Copenhagen, Denmark; 28 Department of Chemistry and Chemical Biology, Harvard University, Cambridge, MA USA; 29 Department of Biochemistry, Boston University Medical Campus, Boston, MA USA; 30 Department of Life Sciences, Imperial College London, London, UK; 31 Instituto de Ciências Biomédicas, Departamento de Parasitologia, Universidade de São Paulo, São Paulo, SP Brazil; 32 Graduate School of Analytical Science and Technology, Chungnam National University, Daejeon, Republic of Korea; 33 Department of Laboratory Medicine, Sahlgrenska Academy, University of Gothenburg, Gothenburg, Sweden; 34 Institute of Biological Chemistry, Academia Sinica, Taipei, Taiwan; 35 Biomolecular Discovery Research Centre, Macquarie University, Sydney, NSW Australia

**Keywords:** Computational platforms and environments, Research data, Software, Glycobiology

## Abstract

Glycoproteomics is a powerful yet analytically challenging research tool. Software packages aiding the interpretation of complex glycopeptide tandem mass spectra have appeared, but their relative performance remains untested. Conducted through the HUPO Human Glycoproteomics Initiative, this community study, comprising both developers and users of glycoproteomics software, evaluates solutions for system-wide glycopeptide analysis. The same mass spectrometry based glycoproteomics datasets from human serum were shared with participants and the relative team performance for *N﻿-* and *O*-glycopeptide data analysis was comprehensively established by orthogonal performance tests. Although the results were variable, several high-performance glycoproteomics informatics strategies were identified. Deep analysis of the data revealed key performance-associated search parameters and led to recommendations for improved ‘high-coverage’ and ‘high-accuracy’ glycoproteomics search solutions. This study concludes that diverse software packages for comprehensive glycopeptide data analysis exist, points to several high-performance search strategies and specifies key variables that will guide future software developments and assist informatics decision-making in glycoproteomics.

## Abstract (web summary)

This analysis presents the results of a community-based evaluation of existing software for large-scale glycopeptide data analysis.

## Main

Protein glycosylation, the attachment of complex carbohydrates (glycans) to discrete sites on proteins, plays diverse roles in biology^1^. The system-wide analysis of intact glycopeptides, or glycoproteomics, aims to study glycan structures, modification sites and protein carriers at scale within a single experiment^2,3^. Facilitated by the recent advances in separation science, mass spectrometry (MS) and informatics, glycoproteomics has matured over past decades and is now ready to tackle biological questions and generate new insights into the heterogeneous glycoproteomes of biological systems^4–7^.

While glycoproteomics studies now routinely report thousands of *N-* and *O*-glycopeptides^8^, accurate identification of glycopeptides from large volumes of mass spectral data remains a bottleneck. The annotation process of glycopeptide MS/MS data is highly error prone due to the challenging task of correctly assigning the glycan composition, modification site(s) as well as the peptide carrier^9–11^. As a result, glycopeptides reported in glycoproteomics publications are frequently misidentified or suffer from ambiguous annotation even in studies attempting to control the false discovery rate (FDR) of assignments.

Diverse fragmentation modes including resonance-activation collision*-*induced dissociation (CID), beam-type CID (higher-energy collisional dissociation; HCD) and electron*-*transfer dissociation (ETD) have proved valuable for glycoproteomics^12–15^. When applied in concert—now possible, for example, on Orbitrap Tribrid mass spectrometers—these fragmentation strategies provide complementary structural information on glycopeptides. Briefly, HCD-MS/MS informs on the peptide carrier and produces useful diagnostic glycan fragments, enabling glycopeptide classification and deduction of generic glycan compositions, ETD-MS/MS reveals in favorable cases the modification site and peptide identity, while resonance-activation CID-MS/MS informs primarily on the glycan composition, sequence and topology^16,17^. Hybrid-type fragmentation strategies including electron-transfer/collision-induced dissociation (ETciD) and electron-transfer/higher-energy collision dissociation (EThcD) are becoming popular given their ability to generate information*-*rich glycopeptide fragment spectra containing multiple fragment types^18^. Accurate mass measurements (<5–10 ppm) at high resolution of precursor and product ions, available on most contemporary instruments, are essential in glycoproteomics. Despite these exciting advances, unambiguous glycopeptide identification remains challenging. Informatics advances are therefore required to ensure accurate glycoproteome profiling to further the field^19^.

Glycoproteomics has seen the development of diverse commercial and academic software showing promise for precise annotation and identification of glycopeptides from MS/MS data^20,21^. While some of these tools are already well established and widely applied in glycoproteomics^22^, the relative performance of software available to the community remains untested, leaving a critical knowledge gap that hinders rapid progress in the field.

Facilitated by the HUPO Human Glycoproteomics Initiative (HGI), we here perform a comprehensive community-based evaluation of existing informatics solutions for large-scale glycopeptide analysis. While informatics challenges undoubtedly still exist in glycoproteomics, our study highlights that several computational tools, some already demonstrating high performance and others showing considerable potential, are available to the community. Importantly, key performance-associated search parameters and high-performance search strategies were identified that may help software developers and users to improve glycoproteomics data analysis in the immediate future.

## Results

### Study design and overview

**Fig. 1:**
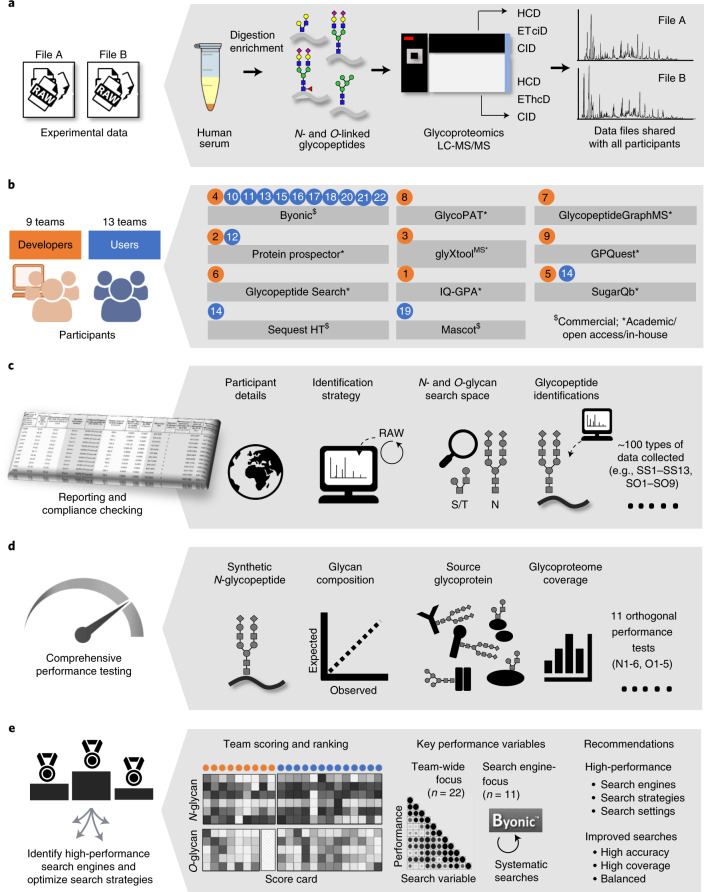
Study overview. **a**, Two glycoproteomics data files of human serum (Files A and B) were generated and shared with participants. **b**, Participants comprising both developers (orange) and users (blue, team identifiers indicated) employed diverse search engines to complete the study. **c**, Teams returned a common reporting template capturing details of the applied search strategy including key search settings (SS1–SS13) and search output (SO1–SO9, Table 1) and their identified glycopeptides. **d**, Complementary performance tests (N1–N6, O1–O5; Table 2) were used to comprehensively evaluate the ability of teams to identify *N-* and *O-*glycopeptides. **e**, The performance profiles were used to score and rank the developers and users separately. Diverse team-wide and search engine-centric (Byonic-focused) approaches were employed to identify performance-associated variables and high-performance search strategies.

Two glycoproteomics data files (Files A and B) were generated using HCD-ETciD-CID-MS/MS and HCD-EThcD-CID-MS/MS of *N*- and *O*-glycopeptides from human serum, respectively (Fig. 1a). A synthetic *N*-glycopeptide was included as a positive control. Serum is a well-characterized biospecimen displaying profound heterogeneity of *N-* and *O-*glycoproteins^23–25^. Thus, Files A and B displayed characteristics (file size, complexity, type) similar to those of data typically encountered in glycoproteomics^26–29^ and were compatible with most search engines.

**Extended Data Fig. 1:**
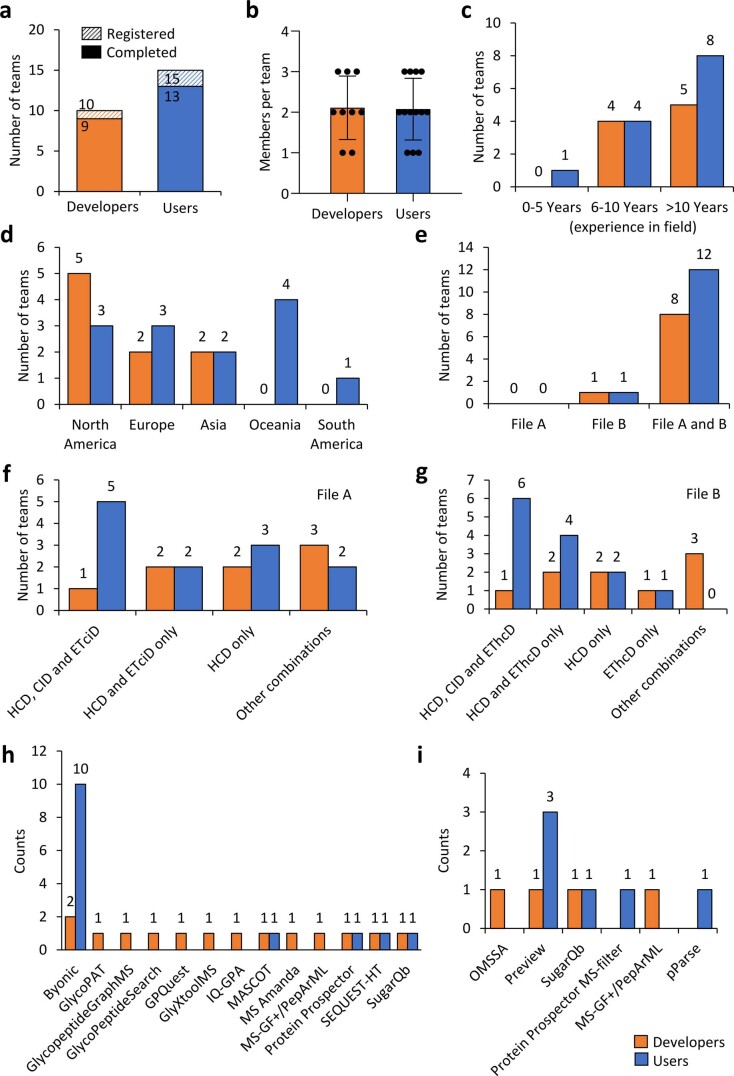
Overview of the participating teams and their search strategies grouped according to their status as either developers (orange) or users (blue) of glycoproteomics software. **a**. Number and type of teams that registered for and completed the study. Note that a few registered teams did not complete the study; individuals within these non-completing teams and their data (if any) were not included in the study outcome. **b**. Average number of members in each of the completing teams. Data is represented by mean ± SD (n = 9, developers and n = 13, users). **c**. The self-reported experience in glycoproteomics of each team. **d**. Team origin by continent. **e**. Data files (File A and/or B) handled by the teams. **f-g**. Type of fragmentation spectra used by teams to identify glycopeptides. **h**. Search engine(s) and **i**. pre- and postprocessing tools used for the glycopeptide identification.

Files A and B were shared with all 22 participating teams, who classified themselves as developers (9 teams) or users (13 teams) of glycoproteomics software (Fig. 1b and Extended Data Fig. 1a,b). All teams identified *N-* and *O-*glycopeptides from Files A and B and reported their approaches and identifications in a standardized reporting template. Most developers (5 teams) and users (8 teams) were experienced in glycoproteomics (>10 years). Participants were from North America, South America, Europe, Asia and Oceania (Extended Data Fig. 1c,d).

Unlike File A, which was processed by 20 of 22 teams (90.9%), File B was processed by all teams (Extended Data Fig. 1e). While most participants reported on spectra acquired with multiple fragmentation methods, a few teams used only HCD- or EThcD-MS/MS for the identifications (Extended Data Fig. 1f,g).

Participants used diverse search engines (Fig. 1b and Extended Data Fig. 1h). Some search engines were used as stand-alone tools or with other software while others were applied with pre- or postprocessing tools to aid the identification (Extended Data Fig. 1i). The developers used nine different glycopeptide-centric search engines, including the following: team 1: IQ-GPA v2.5^30^; team 2: Protein Prospector v5.20.23^31^; team 3: glyXtool^MS^ v0.1.4^32^; team 4: Byonic v2.16.16^33^; team 5: Sugar Qb^34^; team 6: Glycopeptide Search v2.0alpha^35^; team 7: GlycopeptideGraphMS v1.0^36^/Byonic^33^; team 8: GlycoPAT v2.0^37^ and team 9: GPQuest v2.0^38^. Among the 13 users, 10 teams (~75%) used Byonic (teams 10, 11, 13, 15–18 and 20–22), while a few teams used Protein Prospector (team 12), SugarQB/Sequest HT (team 14) and Mascot (team 19) (Supplementary Table 1).

**Extended Data Fig. 2:**
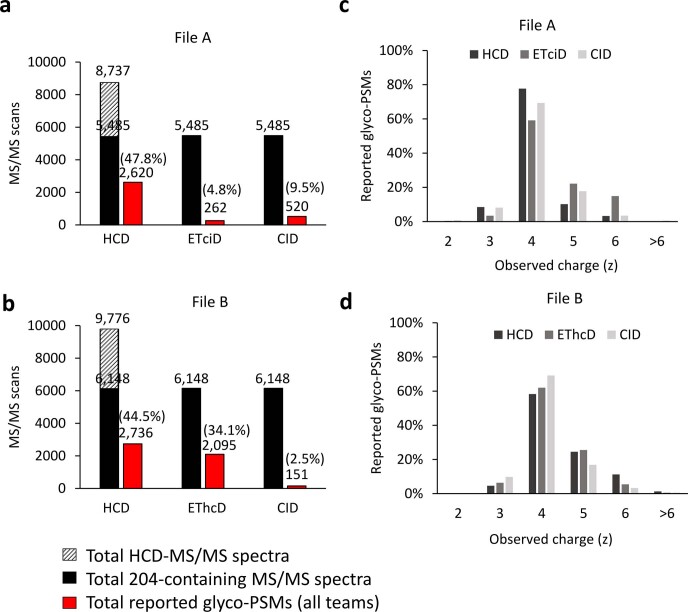
Overview of the MS/MS data and charge state distribution of the reported glycopeptides. **a-b**. The total number of all recorded HCD-MS/MS scans within Files A-B (striped bars), the total number of *m/z* 204-containing MS/MS scans (potential glycopeptide MS/MS spectra, black bars) and the total number of glycoPSMs collectively reported from all teams (red bars) over the different fragmentation methods. **c-d**. Charge state distribution of the reported glycoPSMs from Files A-B (data are plotted as the mean calculated from all teams).

Files A and B contained 8,737/9,776 HCD-MS/MS scans, of which 5,485/6,148 (~63%) spectra contained glycopeptide-specific oxonium ions (e.g., *m/z* 204.0867) used for ETciD/EThcD/CID-MS/MS triggering (Extended Data Fig. 2a,b). Among all potential glycopeptide MS/MS spectra (Files A and B, 16,445/18,444, considering all fragmentation modes), 3,402/4,982 (20.7%/27.0%) nonredundant (unique) glycopeptide-to-spectrum matches (glycoPSMs) were collectively reported by participants. Most teams reported on HCD- and EThcD-MS/MS data, while only a few teams used CID- and ETciD-MS/MS. Similar charge distribution (most frequently quadruply charged precursors) was observed for glycopeptides reported from different fragmentation modes (Extended Data Fig. 2c,d).

A wealth of data was collected via a comprehensive reporting template. The team reports covered intricate details of the employed search strategies and identified glycopeptides (Fig. 1c). Details of the applied search settings were captured including permitted peptide modifications, mass tolerance, postsearch filtering criteria (Supplementary Table 1) and the applied glycan search space (Supplementary Table 2). The search settings (SS1–SS13, Table 1) varied considerably across teams.

**Table 1:** Overview of important study variables including key search settings (SS1–SS13) and search output (SO1–SO9)

**Search settings**		**Type**^**a**^	**Range or definition of category (team count)**
SS1	*N-*glycan search space	Num	23–381 unique *N*-glycan compositions
SS2	*O-*glycan search space	Num	3–223 unique *O*-glycan compositions
SS3	Search engine(s) applied	Cat	Byonic (11), Protein Prospector (2), GlycoPAT (1), GlycopeptideGraphMS (1), glyXtool^MS^ (1), GPQuest (1), other (5)
SS4	Type of search engine	Cat	Academic/open access/in-house = 0 (7), commercial = 1 (15)
SS5	Spectral calibration postacquisition	Cat	No = 0 (18), Yes = 1 (4)
SS6	Protease specificity	Cat	Nontryptic (N*-* or C*-*ragged or nonspecific) = 0 (8), tryptic = −1 (14)
SS7	Missed peptide cleavages permitted	Num	0–2 missed cleavages
SS8	Variable peptide modification(s) (nonglycan)	Num	0–14 nonglycan modification types
SS9	Maximum glycans per peptide	Num	1–5 glycans/peptide
SS10	Maximum other variable modifications	Num	0–5 variable modifications/peptide
SS11	Precursor ion mass error permitted	Cat	Low (<5 ppm) = 0 (6), medium (5–10 ppm) = 1 (14), high (>10 ppm) = 2 (2)
SS12	Product ion mass error permitted	Cat	Low (<5 ppm) = 0 (0), medium (5–10 ppm) = 1 (9), high (>10 ppm) = 2 (13)
SS13	Peptide/protein FDR (decoy/contaminant database)	Cat	No decoy/contaminant = 0 (3), only decoy or contaminant = 1 (9), both decoy and contaminant = 2 (10)
**Search output**		**Type**^**a**^	**Range**
			(*N-* or *O-*glycosylation)
SO1	Glycopeptide LC retention time^b^	Num	41.7–57.2 min (NG)
			26.2–55.8 min (OG)
SO2	Glycopeptide *m/z* (observed)^b^	Num	*m/z* 712.9–1199.4 (NG)
			*m/z* 619.6–1229.0 (OG)
SO3	Glycopeptide charge state (observed)^b^	Num	*z* = 3.9–4.3 (NG)
			*z* = 3.1–5.4 (OG)
SO4	Monoisotopic correction (off-by-*X*, positive values)^b^	Num	0–2.1 Da (NG)
			0–2.0 Da (OG)
SO5	Glycopeptide mass ([M + H]^+^, observed)^b^	Num	3144.6–4913.0 Da (NG)
			1892.9–6057.1 Da (OG)
SO6	Glycopeptide actual mass error (observed, positive values)^b^	Num	0.5–2.8 ppm (NG)
			1.1–5.9 ppm (OG)
SO7	Glycopeptide length^b^	Num	16.9–26.8 AA (NG)
			14.5–38.9 AA (OG)
SO8	Glycan mass (calculated from reported glycopeptides)^b^	Num	1880.8–2410.1 Da (NG)
			195.8–2216.8 Da (OG)
SO9	Reported glycoPSMs	Num	49–2122 glycoPSMs (NG)
			5–578 glycoPSMs (OG)

See Supplementary Tables 1–4 for details. ^a^Num, numerical variable; Cat, categorical variable. NG, *N-*glycosylation; OG, *O-*glycosylation. ^b^Average of output data reported by each team. AA, amino acid residues. While reported glycoPSMs were considered search output (SO9), unique glycopeptides were used to score the glycoproteome coverage (N4, O3).

**Extended Data Fig. 3:**
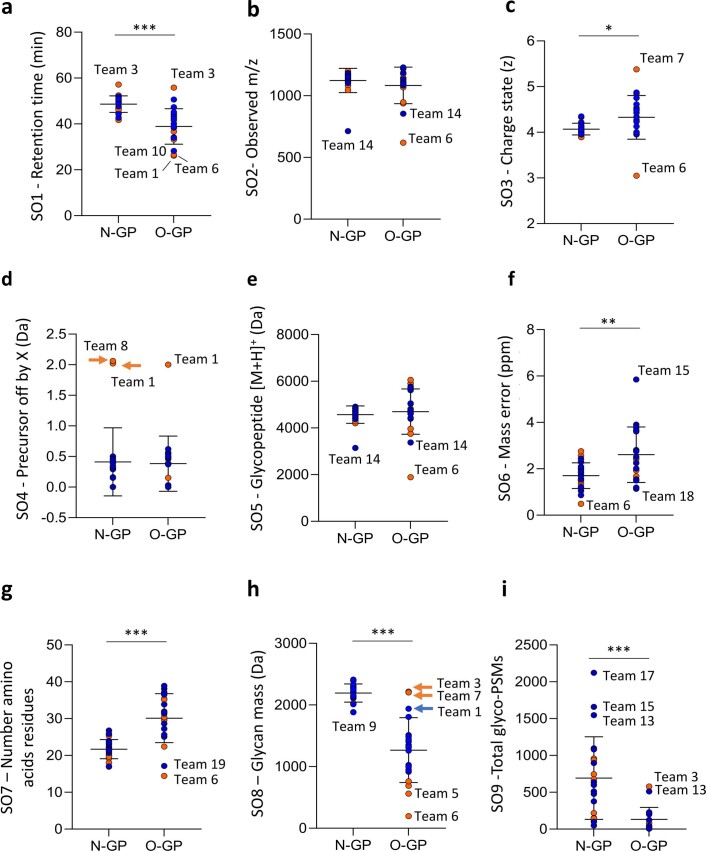
Team-centric overview of the search output data from the glycopeptide identification process (SO1-SO9). Distribution of the **a**. LC retention time (min), ***P = 4.52 ×10^-6^, **b**. observed glycopeptide *m/z*, **c**. observed charge state (z), *P = 1.97 ×10^-2^, **d**. observed precursor selection off-by-X (Da, positive values only), **e**. observed glycopeptide mass [M + H]^+^ (Da), **f**. actual mass error of observed glycopeptides (ppm, positive values only), **P = 2.78 ×10^-3^, **g**. length of observed glycopeptides, ***P = 2.44 ×10^-6^, **h**. glycan mass of observed glycopeptides (M, Da), ***P = 1.03 ×10^-9^, **i**. total *N*- and *O*-glycoPSMs reported by the participants, ***P = 1.02 ×10^-4^. The mean and SDs of data from all teams are also indicated for each graph. Developer data are plotted in orange and user data points are in blue. Teams reporting data outside the SDs have been labelled. The *N*-glycopeptide (N-GP, n = 22) data were statistically compared to the *O*-glycopeptide (O-GP, n = 20) data using unpaired two-sided t-tests where *P < 0.05, **P < 0.01 and ***P < 0.001. See Supplementary Table 4 for data.

Diverse output data arising from the glycopeptide identification process were captured (Supplementary Table 3). The output data also varied notably across teams (Supplementary Table 4). Analysis of key search output variables (SO1–SO9, Table 1) revealed that the reported *N-* and *O-*glycopeptides, as expected, showed different characteristics (e.g., liquid chromatography (LC) retention time, glycan mass) while other characteristics (e.g., observed precursor *m/z*) were similar between the two analyte classes (Extended Data Fig. 3). Analysis of SO1–SO9 data also demonstrated that some teams reported highly discrepant outputs. For example, and without being able to link these observations to performance, the developers of Glycopeptide Search (team 6) and GlycopeptideGraphMS (team 7) reported glycopeptides with unusually low (*z* = ~3^+^) and high (~5.5^+^) charge states relative to other teams (~4.5^+^) (Extended Data Fig. 3c). These output data comparisons may be valuable for developers to better understand, further develop and ultimately improve their software.

**Extended Data Fig. 4:**
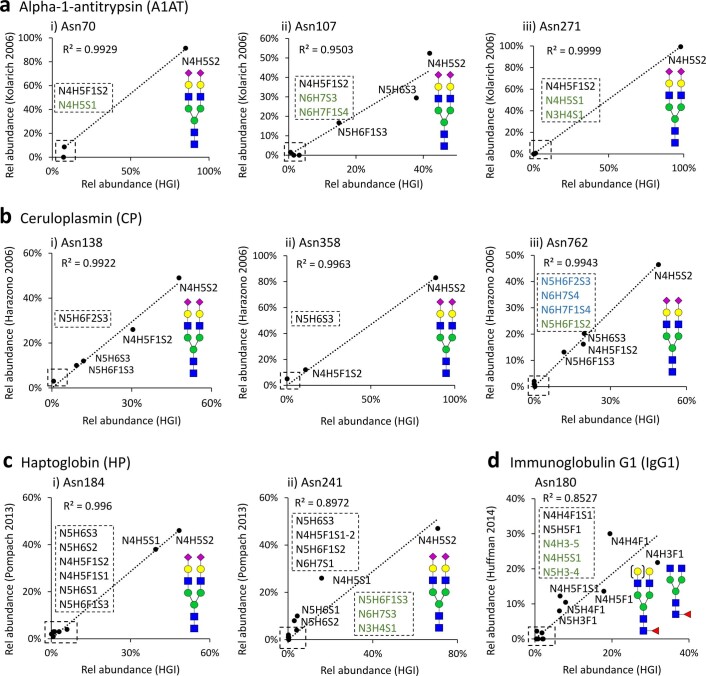
The site-specific *N*-glycosylation of proteins in the investigated serum sample was found to quantitatively match previously reported *N*-glycoform distributions of the same proteins from normal human serum. Four high abundance glycoproteins each harboring multiple *N*-glycosylation sites were selected for this comparison including **a**. alpha-1-antitrypsin (A1AT, P01009), **b**. ceruloplasmin (CP, P00450), **c**. haptoglobin (HP, P00738) and **d**. immunoglobulin G1 (IgG1, P01857). The glycoproteins selected for this analysis are positive acute phase proteins and hence their serum levels and glycosylation features may be altered as a result of physiological changes. The quantitative glycoprofiling (indicated as “Rel abundance (HGI)”) was manually performed using AUC-based quantitation and compared to robust literature reporting on the relative abundance of site-specific glycoforms from the same proteins. The glycoforms have been labelled according to their generic monosaccharide composition (N, HexNAc; H, Hex; F, dHex; S, NeuAc). Cartoons illustrating likely *N*-glycan structures have been provided for the high abundance glycoforms. Low abundance glycoforms were listed according to their relative expression level (high->low, see zoom indicated with broken boxes). Black compositions indicate the glycopeptides reported in literature and found in HGI study; Blue compositions indicate glycopeptides reported only in literature; Green compositions indicate glycopeptides found only in HGI study. The relative abundance (in %) of the individual glycoforms were plotted and correlation coefficients (R^2^) generated for each *N*-glycosylation site. The consistently high correlation between the site-specific glycoprofiles generated from the HGI sample and from the literature (R^2^ = 0.85 – 1.00) validates the use of literature to score and rank the team performance in this study as used for the performance tests N2-N3 and O1-O2 (see Table 2 for details of performance tests).

**Extended Data Fig. 5:**
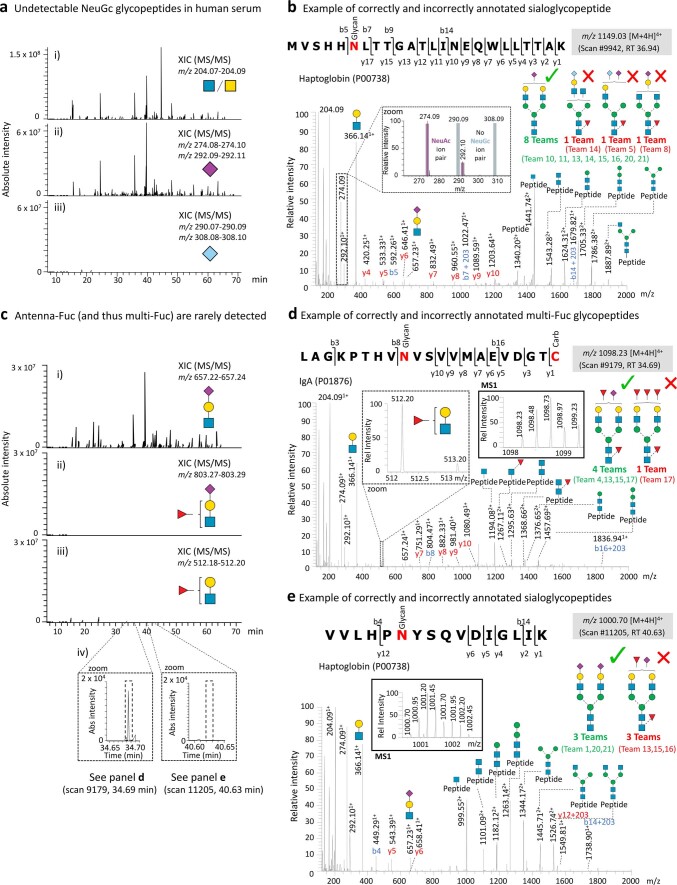
Glycopeptides carrying NeuGc and multi-Fuc signatures are undetectable or rarely detected in the human serum sample investigated in this study. **a**. Extracted ion chromatograms (XICs) were performed at the MS/MS levels for well-established diagnostic oxonium ions, including fragment ions reporting on i) HexNAc, ii) NeuAc, and iii) NeuGc. While abundant diagnostic ions as expected were observed for HexNAc and NeuAc, practically no diagnostic ions were observed for NeuGc glycopeptides. The XIC traces have been plotted on the same absolute intensity scale. All fragmentation modes (HCD, EThcD and CID) were considered for this XIC analysis. Only data from File B reported on by all teams were plotted in this figure; File A showed similar patterns (data not shown). **b**. Example of an HCD-MS/MS spectrum of a NeuAc-containing sialoglycopeptide correctly and incorrectly annotated by teams. Most teams correctly identified that this scan corresponds to a NeuAc glycopeptide as demonstrated by the presence of diagnostic oxonium and B ions for NeuAc, while two teams incorrectly identified the spectrum as a NeuGc-containing glycopeptide despite the absence of diagnostic oxonium and B ions for NeuGc (see insert) and one team incorrectly identified the spectrum as a NeuAc and Fuc containing glycopeptide due to the misidentification of Met oxidation. **c**. XICs were performed at the MS/MS level for well-established diagnostic B ions reporting on different antenna features, including i) sialyl LacNAc, ii) sialyl Lewis x/a, and iii) Lewis x/a. While abundant diagnostic ions as expected were observed for sialyl LacNAc, only very few diagnostic ions were observed for antennary fucosylation features (sialyl Lewis x/a and Lewis x/a). iv) Few diagnostic ions for antenna fucosylation could be observed at very low abundance, which indicated that antenna fucosylation (and thus by extension multi-fucosylated glycopeptides) are present but are rarely detected in the studied serum sample. The XIC traces have been plotted on the same absolute intensity scale. All fragmentation modes (HCD, EThcD and CID) were considered for this XIC analysis. Only data from File B reported on by all teams were plotted in this figure; File A showed similar patterns (data not shown). **d**. Example of an HCD-MS/MS spectrum of a multi-Fuc-containing glycopeptide correctly and incorrectly annotated by teams. Most teams correctly identified that this scan corresponds to a multi-Fuc sialoglycopeptide as indicated by the presence of diagnostic B ions for Lewis x/a (see insert, broken lines) and NeuAc oxonium ions as well as core fucosylated Y1 and Y2 ions, while one team incorrectly identified the spectrum as a tetra-fucosylated asialylated glycopeptide. Note that some teams (for example team 17) reported on several different glycopeptides from the same scan, likely due to conflicting output data from multiple searches of the same data. The monoisotopic precursor ion profile (see insert, full lines) supported that this spectrum corresponds to a difucosylated glycopeptide carrying a single NeuAc. **e**. Example of an HCD-MS/MS spectrum of a NeuAc-containing glycopeptide correctly and incorrectly annotated by teams. Three teams correctly identified that this scan corresponds to a disialylated (NeuAc) afucosylated glycopeptide as indicated by the presence of diagnostic oxonium and B ions for NeuAc, while three teams incorrectly identified the spectrum as a multi-Fuc sialoglycopeptide despite the lack of diagnostic ions for core fucosylated Y1 ions, and sialyl Lewis x/a or Lewis x/a. The monoisotopic precursor ion profile (see insert, full lines) supported that this spectrum corresponds to a disialylated NeuAc glycopeptide not carrying fucose.

The team performance was assessed using orthogonal performance tests that served to comprehensively evaluate the glycopeptide identification accuracy (specificity) and glycoproteome coverage (sensitivity), two key performance characteristics in glycoproteomics (Fig. 1d). Six (N1–N6) and five (O1–O5) performance tests were carefully designed to assess the relative performance for *N-* and *O*-glycopeptide data analysis across teams (Table 2 and Supplementary Tables 5–15). First, the ability to detect the synthetic *N*-glycopeptide in the datasets was assessed (N1). Further, the glycan compositions (N2, O1) and source glycoproteins (N3, O2) of the reported glycopeptides were compared to the established serum glycome and against known serum glycoproteins^4,23,39–42^. To validate the use of the literature to score teams, we performed manual site-specific glycoprofiling of four serum glycoproteins—α-1-antitrypsin (A1AT), ceruloplasmin (CP), haptoglobin (HP) and immunoglobulin G1 (IgG1)—and showed an excellent agreement (*R*^2^ = 0.85–0.99) with relevant literature on healthy human serum^43–46^ (Extended Data Fig. 4). The glycoproteome coverage, on the other hand, was simply the reported nonredundant glycopeptides (N4, O3). Finally, the ability to identify glycopeptides commonly reported by most teams (‘consensus glycopeptides’) (N5, O4) and glycopeptides free of NeuGc and multi-Fuc features (N6, O5) was also scored. We ensured that NeuGc and multi-Fuc glycopeptides, unexpected glycofeatures in human serum^23,47–49^, were indeed absent or rarely detected in Files A and B (discussed below) allowing these to be deemed putative false positives for the purpose of scoring teams (Extended Data Fig. 5).

**Table 2:** Overview of the performance tests applied to establish the relative team performance for glycopeptide data analysis

Performance tests		Description of scoring method
*N-*glycopeptide performance tests		
N1	Synthetic *N-*glycopeptide	Identification accuracy (specificity, %) multiplied by coverage (sensitivity, %) of a synthetic *N*-glycopeptide (Supplementary Table 5)
N2	*N-*glycan composition^a^	Pearson correlation (*R*^2^) between the expected^23^ and observed *N*-glycan distribution in human serum (Supplementary Table 6)
N3	Source *N-*glycoprotein^a^	Specificity and sensitivity of reported source *N*-glycoproteins relative to expected serum glycoproteins^23,39^^,^ (Supplementary Table 7)
N4	*N-*glycoproteome coverage	Unique *N*-glycopeptides reported (unique peptide sequence and *N*-glycan composition) (Supplementary Table 8)
N5	Commonly reported ‘consensus’ *N-*glycopeptides	Proportion of reported *N*-glycopeptides of the consensus *N*-glycopeptides commonly reported by >50% of teams (Supplementary Table 9)
N6	NeuGc and multi-Fuc *N-*glycopeptides (absence)	Proportion of reported *N*-glycoPSMs not containing NeuGc and multi-Fuc of all reported *N*-glycoPSMs. Only applicable if NeuGc/multi-Fuc glycans were included in *N*-glycan search space (Supplementary Table 10)
*O-*glycopeptide performance tests		
O1	*O-*glycan composition^a^	Pearson correlation (*R*^2^) between the expected^40^ and observed *O*-glycan distribution in human serum (Supplementary Table 11)
O2	Source *O-*glycoprotein^a^	Specificity and sensitivity of reported source *O*-glycoproteins relative to expected serum glycoproteins^4,41,42^^,^ (Supplementary Table 12)
O3	*O-*glycoproteome coverage	Unique *O*-glycopeptides reported (unique peptide sequence and *O*-glycan composition) (Supplementary Table 13)
O4	Commonly reported ‘consensus’ *O-*glycopeptides	Proportion of reported *O*-glycopeptides of the consensus *O*-glycopeptides commonly reported by >30% of teams (Supplementary Table 14)
O5	NeuGc and multi-Fuc *O-*glycopeptides (absence)	Proportion of reported *O*-glycoPSMs ﻿not containing NeuGc and multi-Fuc of all reported *O*-glycoPSMs. Only applicable if NeuGc/multi-Fuc glycans were included in *O*-glycan search space (Supplementary Table 15)

The performance tests scored each team using normalized quantitative values (range 0–1). ^a^Some tests were based on matches to data from robust literature on human serum glycosylation^4,23,39–42^. Glycopeptide data from Files A and B showed excellent agreement to the literature (Extended Data Fig. 4).

The performance tests were used to score and rank teams (Fig. 1e and Supplementary Table 16). The developer and user groups were not compared because they received different study instructions. The team scoring was validated using an independent glycoprotein-centric site-specific profiling test (Supplementary Table 17). Finally, performance data from both team-wide and search engine-centric approaches revealed performance-associated search variables and led to improved glycoproteomics search strategies (Supplementary Tables 18 and 19).

### Overview of the reported glycopeptides

**Fig. 2:**
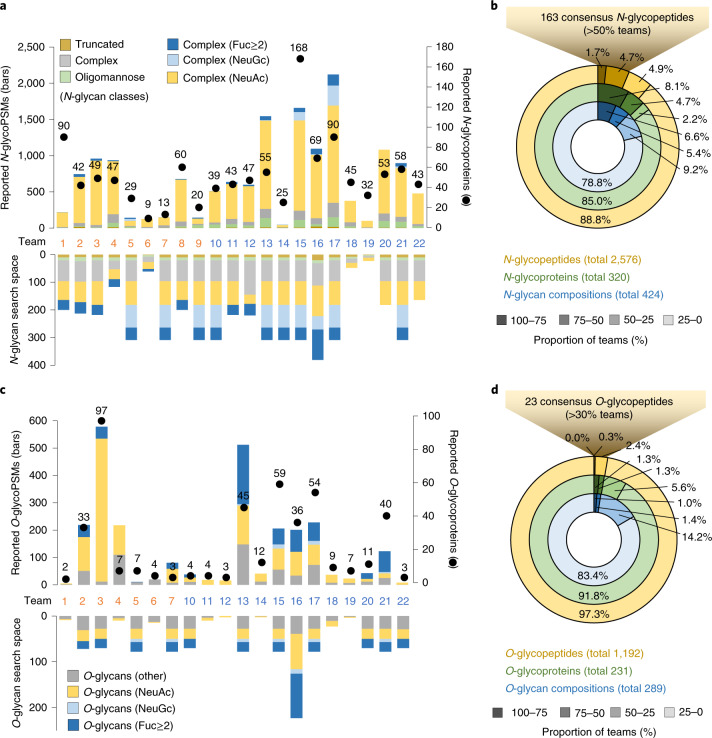
Glycopeptides reported across teams. **a**, Reported *N*-glycoPSMs (bars), unique source *N*-glycoproteins (dots) and the *N*-glycan search space applied (mirror bars) by each team. See key for *N*-glycan classification. **b**, Proportion of *N*-glycopeptides, source *N*-glycoproteins and *N*-glycan compositions commonly reported by teams. **c**, Reported *O*-glycoPSMs, unique source *O*-glycoproteins and the *O*-glycan search space applied by each team. Teams 8 and 9 did not perform *O*-glycopeptide analysis. See key for *O*-glycan classification. Multi-feature *N*- and *O*-glycans fitting into several of these classes were for this purpose classified in a prioritized order of multi-Fuc–NeuGc–NeuAc; see Supplementary Tables 2 and 3 for data. **d**, Proportion of *O*-glycopeptides, source *O*-glycoproteins and *O*-glycan compositions commonly reported by teams. The high-confidence ‘consensus’ *N*- and *O*-glycopeptides have been made publicly available (GlyConnect Reference ID 2943).

The following analyses were carried out using data reported from File B processed by all teams. The total *N-*glycoPSMs (49–2,122) and source *N*-glycoproteins (9–168) reported by the 22 teams varied dramatically (Fig. 2a and Supplementary Table 3). In line with the literature on human serum *N*-glycosylation^23,24^, the reported *N-*glycopeptides carried mainly complex-type *N-*glycans (92.6%, average across teams). Relatively few oligomannosidic (6.4%) and truncated (herein defined as Hex_<4_HexNAc_<3_Fuc_<2_ or biosynthetically unusual *N*-glycans) (1.0%) *N-*glycopeptides were reported. The applied *N-*glycan search space spanned an equally wide range (23–381 compositions) comprising mostly complex-type *N*-glycans (89.1%) and the less heterogeneous oligomannosidic (5.9%) and truncated (5.0%) *N-*glycans. No associations were found between the size of the *N-*glycan search space and reported *N-*glycoPSM counts (Pearson *R*^2^ = 0.115). Unexpected glycan compositions including NeuGc and multi-Fuc-containing complex-type *N-*glycans, which are negligible features of human serum glycoproteins^23,47–49^, were not only included in the glycan search space (up to 26.5% and 28.9%, respectively), but also reported (up to 20.6% and 5.0%) by some teams. The absence of NeuGc and the rarity of multi-Fuc glycopeptides in the shared data was supported by a lack of diagnostic fragment ions for NeuGc (*m/z* 290/308), scarcity of antenna Fuc ions (*m/z* 512/803) and the frequent mis-annotation of MS/MS spectra claimed to correspond to NeuGc and multi-Fuc glycopeptides (Extended Data Fig. 5). While only infrequently detected, multi-Fuc glycopeptides were, however, evidently present in our data as supported by manual spectral annotation (Extended Data Fig. 5d).

**Extended Data Fig. 6:**
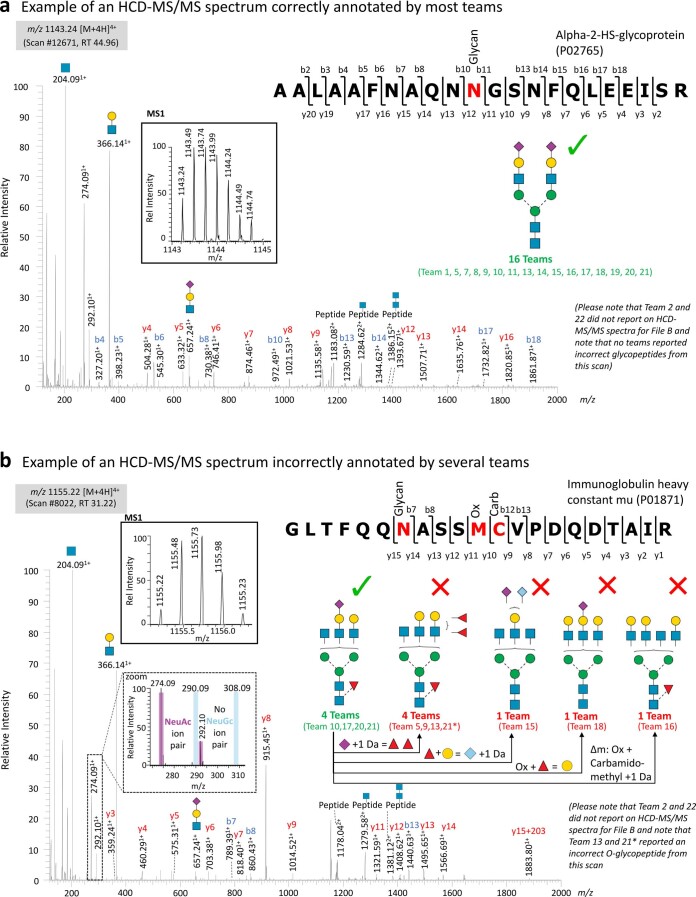
Examples of (in)correctly annotated *N*-glycopeptides. **a**. HCD-MS/MS fragment spectrum of a ‘consensus’ NeuAc-containing sialoglycopeptide correctly annotated by all 16 teams (teams 1, 5, 7, 8, 9, 10, 11, 13, 14, 15, 16, 17, 18, 19, 20, 21) reporting on this particular scan number. Manual annotation confirmed that this spectrum indeed corresponds to the indicated NeuAc-containing *N*-glycopeptide from human alpha-2-HS-glycoprotein (UniProtKB, P02765) as demonstrated by the presence of diagnostic oxonium and B ions for NeuAc and extensive b- and y-ion peptide backbone fragmentation. Further, the monoisotopic precursor ion profile (see insert) supported the annotation of this spectrum. **b**. HCD-MS/MS spectrum of a NeuAc-containing core-fucosylated glycopeptide that was incorrectly annotated by several teams. While four teams (teams 10, 17, 20, 21) correctly identified that this spectrum corresponds to an *N*-glycopeptide from human immunoglobulin heavy constant mu (P01871) carrying a single NeuAc and Fuc as indicated by the presence of diagnostic oxonium and B ions for NeuAc (see insert, broken lines), y-ions confirming Met oxidation and Cys carbamidomethylation, and correct monoisotopic precursor ion profile, four incorrect glycan structures were reported by other teams as indicated. The structural differences between the incorrectly and correctly assigned glycans have been indicated in attempts to rationalize the misidentification. All teams (except for team 1, who reported a different peptide from a different source protein with an incorrect precursor *m/z*, data not shown) identified the correct peptide sequence, although the Met oxidation and Cys carbamidomethylation were features that frequently led to incorrect glycopeptide identification. Some teams (for example team 21) reported on several glycopeptides from the same scan, likely due to conflicting output data from multiple searches of the same data. The monoisotopic precursor ion profile (see insert, full lines) and the subsequent EThcD-MS/MS scan (scan #8026, data not shown) supported that this spectrum, in fact, corresponds to the indicated *N*-glycopeptide carrying Met oxidation and Cys carbamidomethylation as well as an *N*-glycan displaying a composition corresponding to a complex *N*-glycan structure with a single NeuAc and Fuc.

Collectively, 2,556 unique *N-*glycopeptides (defined herein as unique peptide sequences and glycan compositions), covering 320 different source *N-*glycoproteins and 424 different *N-*glycan compositions, were reported across teams (Fig. 2b and Supplementary Tables 6, 7 and 9). Of these, only 43 *N*-glycopeptides (1.7%), 26 source *N*-glycoproteins (8.1%) and 28 *N*-glycan compositions (6.6%) were commonly reported by at least 75% of teams (see Extended Data Fig. 6a for an example of congruent spectral annotation across teams). Most glycopeptides, however, were commonly reported by only a few teams, probably due to frequent mis-annotation of the spectral data (Extended Data Fig. 6b).

Notably fewer, but equally discrepant, *O-*glycopeptides (5–578 *O-*glycoPSMs) were reported by participants (Fig. 2c and Extended Data Fig. 3i). As expected, most reported *O-*glycopeptides carried Hex_1_HexNAc_1_NeuAc_1-2_^40,50^. The applied *O*-glycan search space also varied dramatically (3–223 glycan compositions). Similar to the *N*-glycopeptide analysis, no association was observed between the *O-*glycan search space and reported *O-*glycoPSM counts (Pearson *R*^2^ = 0.118). Instead, many other associations were identified (discussed below). While seven teams included NeuGc in the applied *O*-glycan search space (up to 9.0%), only four teams reported NeuGc *O*-glycopeptides (up to 7.3%). In addition, 12 teams included multi-fucosylated glycans in the *O*-glycan search space (up to 43.5%); 11 of those teams reported multi-fucosylated *O*-glycopeptides (average of 28.6%, up to 61.8%). Both NeuGc and multi-fucosylated *O*-glycans are negligible features of human serum *O*-glycoproteins as supported by the literature^40,50^ and our own analyses (above). The reported multi-fucosylated *O*-glycan compositions could, in principle, in some cases arise from multiple discrete *O*-glycans residing on the same peptide. As *O*-glycosylation sites were inconsistently and/or ambiguously reported by most teams (below) we were not able to assess this aspect further.

Collectively, 1,192 unique *O-*glycopeptides covering 231 different source *O-*glycoproteins and 288 different *O-*glycan compositions were identified, but surprisingly few *O-*glycopeptides were commonly reported across teams. Only three *O-*glycopeptides (0.3%), six source *O-*glycoproteins (2.6%) and seven *O-*glycan compositions (2.4%) were commonly reported by at least half the teams (Fig. 2c). Most *O*-glycopeptides were reported by a single or few teams.

**Extended Data Fig. 7:**
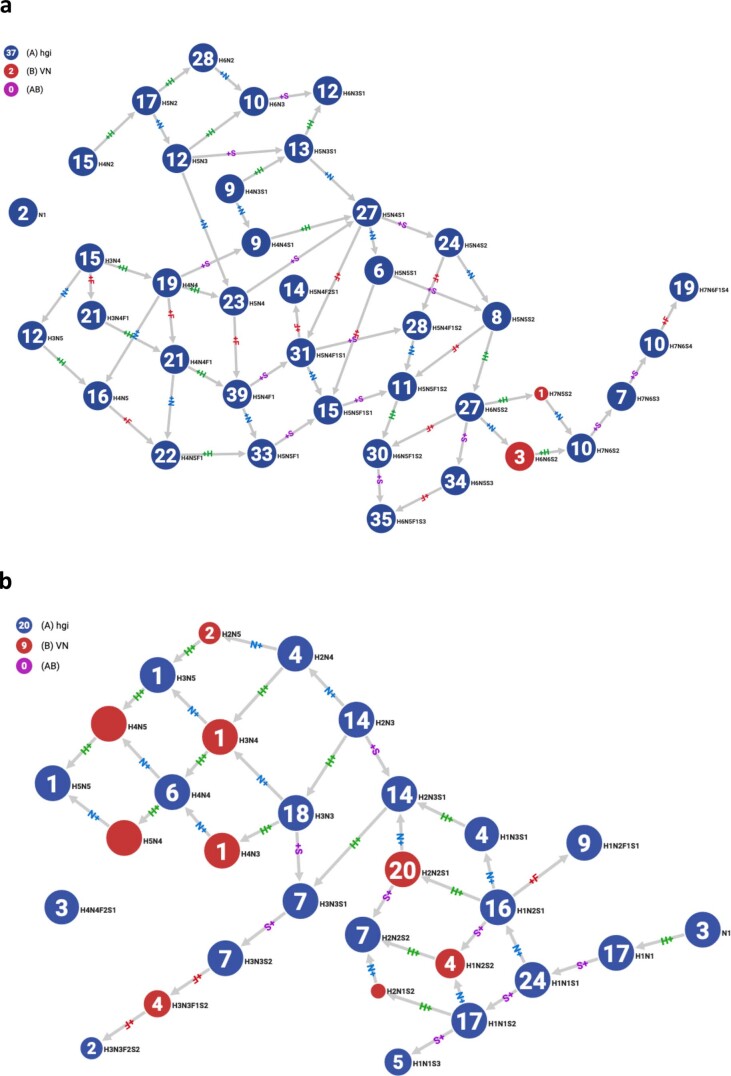
Biosynthesis-centric network analysis of the *N*- and *O*-glycan compositions of the consensus glycopeptides. Biosynthesis-centric network analysis of the *N*- and *O*-glycan compositions carried by the **a**. 163 consensus *N*-glycopeptides and **b**. 23 consensus *O*-glycopeptides using Glyconnect Compozitor v1.0.0. Each node corresponds to a glycan composition either reported within the consensus list of glycopeptides arising from this study (blue circles) or manually added to biosynthetically connect the glycan compositions by a single glycan processing step (red circles). Both networks showed close biosynthetic relationship between the consensus *N*- and *O*-glycan structures reported in this study supporting the correctness of their identification.

Despite the discrepant reporting, high-confidence lists spanning 163 *N-* and 23 *O*-glycopeptides commonly reported by teams could be generated. Importantly, these consensus glycopeptides mapped to expected serum glycoproteins; for example, α-2-macroglobulin (UniProtKB, P01023) and haptoglobin (P00738) and carried expected serum *N*-glycans; for example, Hex_5_HexNAc_4_Fuc_0-1_NeuAc_2_ (GlyTouCan IDs, G09675DY/G22754FQ) and *O*-glycans; for example, Hex_1_HexNAc_1_NeuAc_1-2_ (G65285QO/G84906ML) that were biosynthetically related (Extended Data Fig. 7), devoid of NeuGc and poor in multi-Fuc, further supporting their correct identification. These high-confidence glycopeptides form an important reference to future studies of the human serum glycoproteome and have therefore been made publicly available (GlyConnect Reference ID 2943).

### High-performance informatics solutions for *N*-glycoproteomics

**Extended Data Fig. 8:**
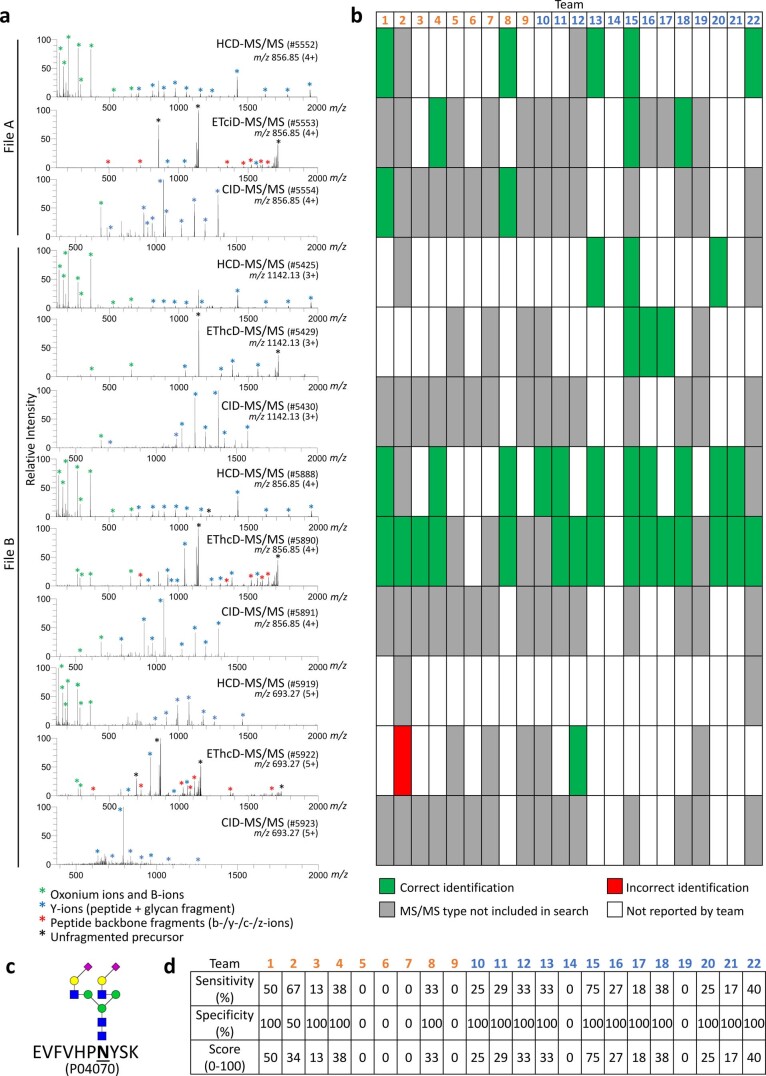
Data underpinning the synthetic *N*-glycopeptide performance test (N1). **a**. MS/MS spectra corresponding to the non-adducted synthetic *N-*glycopeptide (EVFVHPNYSK, Hex_5_HexNAc_4_NeuAc_2_, UniProtKB, P04070) in charge state 3^+^ and 4^+^ (9 top spectra) and the K^+^-adducted synthetic *N-*glycopeptide in charge state 5^+^ (three bottom spectra) arising from the four fragmentation modes (HCD-, ETciD-, EThcD- and CID-MS/MS) used to generate File A and B. Green asterisks: Oxonium ions and non-reducing end glycan fragments (B-ions). Blue asterisks: Y-ion series (peptide conjugated with glycan fragment). Red asterisks: Peptide backbone b-/y-/c-/z-ions. Black asterisks: Unfragmented peptide without glycan, unfragmented precursor (peptide with glycan) and charge-reduced precursor. **b**. Overview of the 12 MS/MS spectra of the synthetic *N-*glycopeptide (from panel a) that were either correctly identified (green), incorrectly identified (red), or not reported by each team (white). Spectra arising from fragmentation mode(s) not included in the search strategy chosen by each team were not included in the assessment (indicated in grey). **c**. Structure of the synthetic *N*-glycopeptide spiked into the human serum sample. **d**. Performance scores arising from the test determined for each team based on the sensitivity and specificity of the identification of the 12 MS/MS spectra corresponding to the synthetic *N-*glycopeptide.

The relative team performance for *N*-glycoproteomics was comprehensively assessed using six independent performance tests (N1–N6) (Table 2 and Supplementary Tables 5–10). Among these performance tests, N1 scored the ability to accurately identify a synthetic *N*-glycopeptide in the sample (Extended Data Fig. 8). Similar to the other performance tests, N1 was used to establish the relative team performance. Founded on a ‘ground truth’, the N1 data including the 12 manually annotated spectra all corresponding to the synthetic *N*-glycopeptide are particularly informative and may aid developers train algorithms and improve software to annotate *N*-glycopeptide spectral data better. The N1 data also supported observations made across the entire dataset (Extended Data Fig. 2) confirming that glycopeptides were preferentially identified in charge state 4^+^ using HCD- and EThcD-MS/MS even when high-quality MS/MS data from other charge states and fragmentation modes were available.

**Extended Data Fig. 9:**
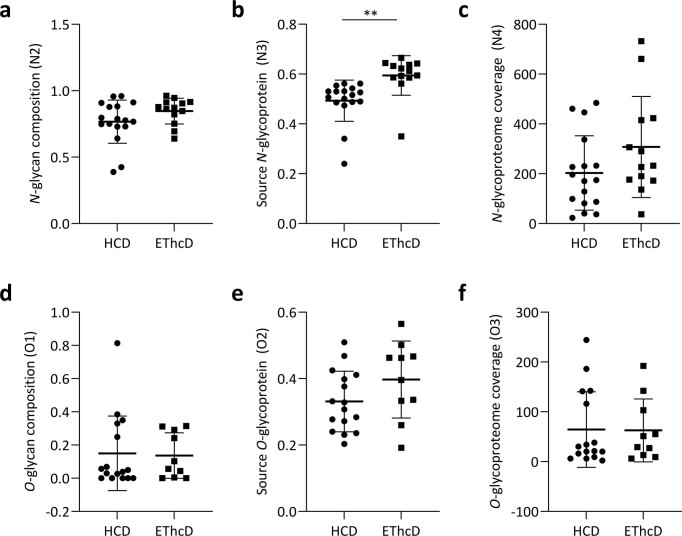
Comparison of the raw (before normalization) performance scores arising from the glycopeptide identifications based on HCD- or EThcD-MS/MS data. Only glycopeptides unambiguously reported by either HCD- or EThcD-MS/MS data were included in this analysis. **a**. *N-*glycan composition (N2), **b**. source *N*-glycoprotein (N3), and **c**. *N*-glycoproteome coverage (N4) were calculated using HCD-MS/MS glycoPSMs reported by 17 teams and EThcD-MS/MS glycoPSMs reported by 13 teams. **d**. *O*-glycan composition (O2), **e**. source *O*-glycoprotein (O2) and **f**. *O*-glycoproteome coverage (O3) were calculated using HCD-MS/MS glycoPSMs reported by 16 teams and EThcD-MS/MS glycoPSMs reported by 10 teams. Significance was tested between the HCD- and EThcD-MS/MS data for all performance scores using unpaired two-sided t-tests where ** indicates *P* = 0.0021.

In line with the literature^2,8^, most teams employed HCD- and/or EThcD-MS/MS for glycopeptide identification. While these two fragmentation modes displayed similar performance in tests scoring the glycan composition (N2, O1) and glycoproteome coverage (N4, O3), higher scores were achieved for EThcD-based relative to HCD-based identifications in the source glycoprotein tests (N3, O2) (Extended Data Fig. 9). Importantly, accurate glycosylation site localization, not tested with this study (discussed below), is a recognized strength of EThcD-MS/MS data^5,12^.

**Fig. 3:**
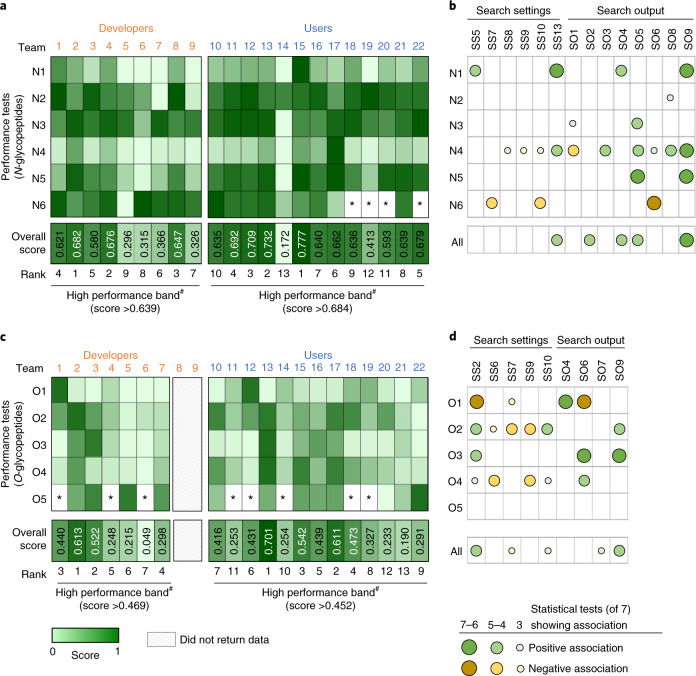
Team scoring/ranking and identification of performance-associated variables. **a**, Heatmap representation of normalized scores (range 0–1) from the *N*-glycopeptide performance tests (N1–N6, Table 2). See Supplementary Tables 5–16 for performance data. ^#^The top third performing teams (white font) were placed in a high-performance band. The team scoring was later validated (Extended Data Fig. 10). *Performance could not be determined. **b**, Many variables (search settings, search output) showed associations (negative or positive) with *N*-glycopeptide performance. See Table 1 for variables. See Supplementary Table 18 for statistics. See **d** for key to symbols. **c**, Scores from the *O*-glycopeptide performance tests (O1–O5). Teams 8 and 9 did not return *O*-glycopeptide data. **d**, Many associations between the search variables and *O*-glycopeptide performance were observed.

The performance tests were used to score and rank developers and users (Fig. 3a). At a glance, the scorecard pointed to considerable team-to-team variations in the performance profiles suggesting that the applied software and search strategies exhibit markedly different strengths and weaknesses for *N*-glycoproteomics. As an example, IQ-GPA (team 1) and GlycoPAT (team 8) performed well (relative to other developers) in the *N-*glycan composition test (N2), while Protein Prospector (team 2) and Byonic (team 4) performed well in tests scoring the source *N-*glycoproteins (N3) and *N-*glycoproteome coverage (N4).

Overall, Protein Prospector (team 2, overall score 0.682), Byonic (team 4, 0.676) and GlycoPAT (team 8, 0.647) were found to be high-performance software solutions for *N*-glycoproteomics. Notably, our scoring did not separate these three developers by any substantial margin, but their overall performance was slightly higher than that of IQ-GPA (team 1, 0.621) and glyXtool^MS^ (team 3, 0.580) and substantially higher than that of the other software (score range 0.296–0.366).

**Extended Data Fig. 10:**
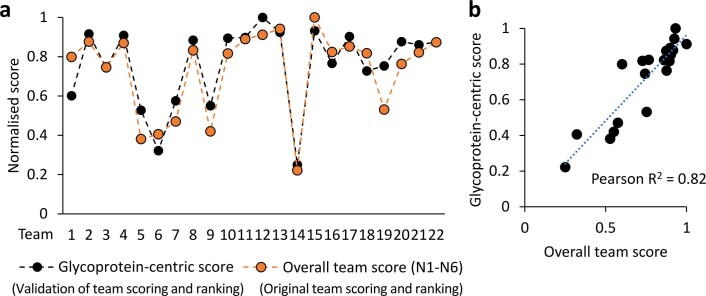
Orthogonal glycoprotein-based scoring to validate the team scoring and ranking. **a**. The overall team scores (best performer normalized to 1) from multiple performance tests (N1-N6, orange dots) and the independent glycoprotein-centric scores (black dots, normalized) showed high similarity across teams. **b**. Pearson correlation analysis confirmed that the overall team scores and the glycoprotein-centric scores correlated across the 22 teams thereby validating the team scoring and ranking (see scorecard, Fig. 3 and Supplementary Table 17 for data).

Supporting our scoring method, an independent assessment method based on the match between reported and actual site-specific *N*-glycoforms of four serum glycoproteins (A1AT, CP, HP, IgG1, thus founded on a ‘ground truth’) recapitulated the scoring profile across teams (*R*^2^ = 0.82) (Extended Data Fig. 10). Further supporting the top ranking of Byonic and Protein Prospector, the best performing user teams employed Byonic (teams 11, 13, 15, score range 0.687–0.777) and Protein Prospector (team 12, 0.709). Their overall performance scores were marginally higher than seven other Byonic users (teams 10, 16–18, 20–22, 0.593–0.679), but markedly higher than teams using SugarQb (team 14, 0.172) and Mascot (team 19, 0.413). Despite the similar overall performance among most user teams, not least the ten Byonic users, their performance profiles differed markedly across the six performance tests.

We then explored the scorecard for software-independent performance-associated variables including the search settings (SS1–SS13) and search output (SO1–9) using seven different statistical methods (Fig. 3b). Many statistically strong relationships were found revealing key performance-associated variables that either positively or negatively correlated with the glycopeptide identification efficiency. As an example, the use of decoy/contaminant databases (SS13) showed associations with performance in the synthetic *N*-glycopeptide test (N1) and high *N-*glycoproteome coverage (N4). Search strategies that allowed for a relatively high diversity and number of nonglycan variable peptide modifications (SS8, SS10) and few glycans per peptide (SS9) were also associated with high *N-*glycoproteome coverage (N4). As expected, allowing multiple missed peptide cleavages (SS7) and variable nonglycan modifications (SS10) in the search strategy correlated with higher glycopeptide FDRs as indicated by higher rates of NeuGc and multi-Fuc identifications (low N6 scores) (Supplementary Table 18).

The association analyses also identified many interesting relationships between the search output and performance (Fig. 3b). Intuitively, teams that reported many *N*-glycoPSMs (SO9) performed well in the synthetic *N-*glycopeptide test (N1), had a higher *N-*glycoproteome coverage (N4) and identified more consensus *N-*glycopeptides (N5). Further, teams that reported glycopeptides featuring a relatively high glycan mass (SO8) more often identified the correct glycan composition (N2), while teams that reported glycopeptides exhibiting relatively high molecular masses (SO5) more often identified the correct source *N-*glycoproteins (N3). Glycopeptides displaying relatively high molecular masses (large glycans and/or peptides) are less likely to be incorrectly identified due to fewer theoretical glycopeptide candidates (fewer potential false positives) in the higher mass range. In addition, early LC retention time (SO1), high charge (SO3), high glycopeptide mass (SO5), high actual mass error (low mass accuracy, SO6) and high glycan mass (SO8) were search output linked to high *N-*glycoproteome coverage (N4). Teams reporting *N*-glycopeptides with high molecular masses (SO5) more often identified consensus glycopeptides (N5). Finally, low actual mass error (SO6) was, as expected, associated with better identification accuracy.

### High-performance informatics solutions for *O*-glycoproteomics

Protein Prospector (team 2) displayed the highest performance in tests scoring the source *O-*glycoproteins (O2) and consensus *O-*glycopeptides (O4) (Fig. 3c and Supplementary Table 16). Conversely, IQ-GPA (team 1) and glyXtool^MS^ (team 3) were the best performing software in tests scoring the *O-*glycan compositions (O1) and *O-*glycoproteome coverage (O3), respectively. Overall, Protein Prospector (team 2, overall score 0.613) and glyXtool^MS^ (team 3, 0.522) were found to be high-performance software for *O*-glycoproteomics. Among the users, four Byonic teams (teams 13, 15, 17, 18, overall score range 0.473–0.701) were ranked in the high-performance band.

Correlation analyses showed that accurate identification of the *O*-glycan compositions (O1) associated with approaches using a focused (narrow) *O-*glycan search space (SS2) and permitting only few missed peptide cleavages (SS7) (Fig. 3d and Supplementary Table 18). In addition, search strategies permitting incorrect precursor selection (SO4) were commonly used by teams scoring well in the *O*-glycan composition test (O1). Interestingly, employing a broad *O*-glycan search space (SS2) was associated with accurate identification of source *O-*glycoproteins (O2), high *O-*glycoproteome coverage (O3) and better identification of consensus *O-*glycopeptides (O4). Further, teams reporting identifications with low mass error (SO6) scored well in the *O-*glycan composition test (O1), but, notably, at the cost of lower *O-*glycoproteome coverage (O3) and fewer consensus *O-*glycopeptides (O4).

### Search engine-centric analysis

**Fig. 4:**
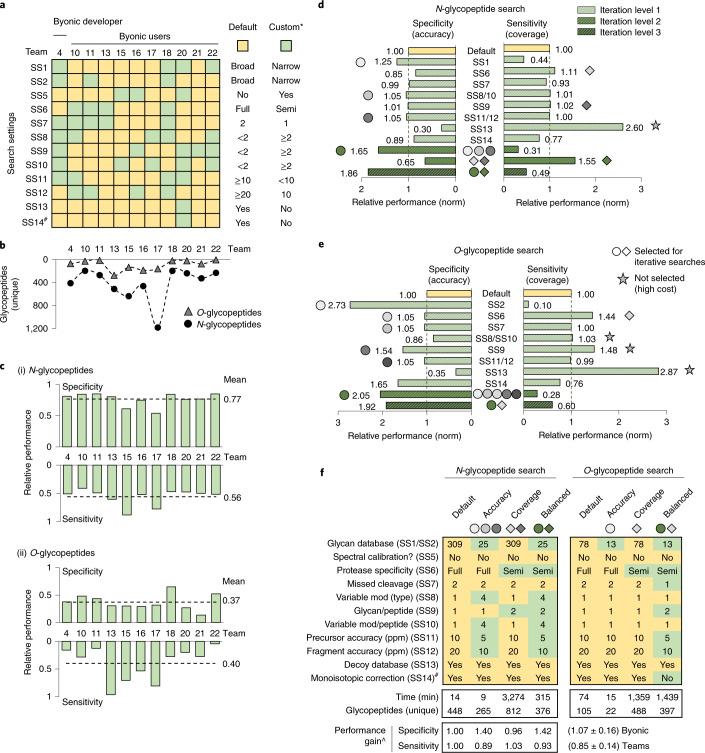
Search engine-centric (Byonic-focused) analysis of search strategies for high-performance glycoproteomics data analysis. **a**, Overview of the search settings employed by Byonic teams. Default: search strategy used by most teams (yellow). Custom: variations from the default search strategy (green). ^#^Data for SS14, a setting not included in the team reports, were adopted from SO4 data. **b**, The glycoproteome coverage (unique glycopeptides, File B) varied among Byonic teams. **c**, Specificity (accuracy) and sensitivity (coverage) scores for (i) *N*-glycopeptides and (ii) *O*-glycopeptides for Byonic teams. **d**, Controlled (in-house) searches for *N-*glycopeptides using Byonic (File B). Individual search settings were systematically varied (iteration level 1) and output assessed for performance gains (specificity, sensitivity). Search settings showing performance gains (shaded circles/diamonds) without unacceptable costs in specificity (SS13) or search time (SS8/SS10, SS9; see examples in **e**) (gray stars) were collectively tested for synergistic performance gains (iteration levels 2 and 3, dark green). See **e** for shared symbol key. **e**, Byonic-centric *O*-glycopeptide searches. See **d** for details. **f**, Recommended Byonic-centric search strategies for ‘high accuracy’, ‘high coverage’ and ‘balanced’ (between accuracy and coverage) glycoproteomics data analysis. ^The recommended search strategies showed relative performance gains as determined using an independent glycoprotein-centric score (Supplementary Table 19b). Search time and glycoproteome coverage (unique glycopeptides) are also indicated.

We then explored the impact of different search strategies on the glycoproteomics data output for the popular Byonic search engine used by 11 teams. The Byonic teams employed highly diverse search strategies; except for the common use of decoy/contaminant databases (SS13) and monoisotopic correction (SS14), the search settings varied considerably across these teams (Fig. 4a). Undoubtedly, this search diversity and different output filtering methods used by the Byonic teams (e.g., Byonic score >100, PEP-2D <0.001, FDR <1%) contributed to the dramatic variation in reported glycopeptides (Fig. 4b and Supplementary Table 1). Unsurprisingly, therefore, the relative specificity (accuracy) and sensitivity (coverage) scores (established from N1–N6/O1–O5) showed different performance profiles of the Byonic teams particularly for the *O*-glycopeptide analysis (Fig. 4c and Supplementary Table 16). Teams achieving better than average sensitivity scores (e.g., teams 15 and 17), typically under-performed with respect to specificity. Other teams achieved higher than average specificity scores at the cost of sensitivity (e.g., teams 18 and 22), confirming the intuitive reciprocal relationship between these performance metrics.

The individual search variables were then investigated through a series of controlled (in-house) searches using Byonic. For this purpose, the search settings were systematically varied from the ‘default’ search strategy used by most teams while keeping other parameters constant. Several search settings showed performance gains in terms of improved specificity (e.g., literature-guided narrow glycan search space, SS1–SS2) or sensitivity (e.g., decoy database disabled, SS13) but often at the expense of other performance characteristics (Fig. 4d,e and Supplementary Table 19a). While reduced sensitivity (glycoproteome coverage) may be an acceptable compromise for higher specificity (identification accuracy), the opposite arguably does not hold true. Thus, the considerable sensitivity gains and concomitant loss in specificity achieved by disabling the decoy database (SS13) did not benefit the data analysis. Instead, increasing the permitted glycans per peptide (SS9), tightening the allowed mass error (SS11–SS12) and relaxing the protease specificity benefitted both specificity and sensitivity. Search settings that showed cost-less performance gains were combined for subsequent rounds of iterative searches. Importantly, these efforts led to improved ‘high accuracy’, ‘high coverage’ and ‘balanced’ (accuracy >< coverage) search strategies for *N-* and *O*-glycoproteomics (Fig. 4f). None of the Byonic teams had utilized these combinations of search settings. When assessed using the independent glycoprotein-centric scoring method, these three recommended search strategies showed improved performance (specificity, sensitivity) relatively to the default strategy and strategies used by Byonic teams (Supplementary Table 19b). Notably, the high-coverage searches dramatically expanded the search time, a metric here not considered beyond logistic constraints.

Finally, we explored the performance of different fragmentation methods by systematically varying the spectral input (HCD/EThcD/CID) in Byonic while keeping search settings and output filtering constant. The highest performance was achieved when HCD and EThcD were jointly searched (Supplementary Table 19c). Our analysis also suggested that low-resolution CID data do not benefit the Byonic search performance when used alone or with HCD/EThcD data, an observation supported by the Byonic team comparison (Supplementary Table 19d).

## Discussion

This community study has objectively discerned the performance of current informatics solutions for glycoproteomics data analysis. Excitingly, several high-performance glycoproteomics software and search strategies were identified. Among the nine developer teams, Protein Prospector (team 2) was identified as the top performing software for both *N-* and *O*-glycoproteomics. Byonic (team 4) also displayed high performance for *N*-glycopeptide data analysis, and while this developer only demonstrated moderate performance for *O*-glycoproteomics, four Byonic user teams (teams 13, 15, 17 and 18) displayed the highest performance for *O*-glycopeptide data analysis. Protein Prospector^51^ and Byonic^33^, developed 10–20 years ago, have pioneered the glycopeptide informatics field and are search engines already commonly used in glycoproteomics^8,31,33^.

Protein Prospector is an academic (free) tool recognized for its ability to identify modified peptides and modification site(s) from LC-MS/MS data using a probability–difference-based scoring system^31^. Protein Prospector is often a preferred search engine in studies addressing the challenging site annotation of *O*-glycopeptides, in particular when ET(hc)D-MS/MS data are available^5^. However, Protein Prospector does not estimate the FDR of the glycan components of glycopeptides, which it regards as nondescript post-translation modifications with an exact mass, and the software may appear less user-friendly than competing tools.

Facilitated by a user-friendly interface, precise spectral annotation and useful output reports of identified glycopeptides/proteins, the commercial Byonic search engine has gained considerable popularity (as illustrated herein) as it enables relatively straightforward identification of peptides with known and unknown modifications including glycosylation from different MS/MS data. Byonic features useful fine control options that enable tailored glycopeptide searches and postsearch filtering of output based on prior knowledge. Byonic scores and annotates multiple types of glycopeptide fragments to deduce the peptide carrier, glycan and modification site, but the FDRs, calculated identically for nonglycosylated peptides and glycopeptides, primarily address the correctness of the peptide rather than the glycan and the site localization^33^.

Notably, GlycoPAT^37^ (team 8) and glyXtool^MS^^32^ (team 3) were in our study also identified as high-performance *N-* and *O*-glycoproteomics software, respectively. Furthermore, IQ-GPA^30^ (team 1) demonstrated merit for both *N-* and *O*-glycopeptide data analysis. While all three software packages handle high-resolution HCD-, ETciD- and EThcD-MS/MS data, IQ-GPA and GlycoPAT also identify glycopeptides based on high-resolution CID-MS/MS data and apply postsearch filtering based on advanced peptide and glycan decoy methods to estimate both peptide and glycan FDRs of glycopeptide candidates. The software glyXtool^MS^ instead uses oxonium ions, Y_1_-ions (peptide-HexNAc), other glycopeptide-specific fragments and peptide-specific b-/y-ions to control FDR. These three academic tools were recently developed (<5 years ago) and, thus, hold a considerable potential in the field.

We used both team-wide and search engine-centric approaches to uncover performance-associated search variables for glycoproteomics data analysis. The team-wide correlation analyses revealed many search settings and search output linked to performance. Backed by robust statistics, these ‘universal’ relationships existing across search engines will widely benefit glycoproteomics software developers and users aiming to improve *N-* and *O*-glycopeptide data analysis (Table 3). This knowledge may aid tackling existing challenges in glycoproteomics, among the most critical, reducing the FDR of glycopeptide candidates carrying glycans with similar (e.g., NeuAc-R versus Fuc_2_-R, Δm = 1.0204 Da) or identical (e.g., NeuAc_1_Hex_1_-R versus NeuGc_1_Fuc_1_-R, Δm = 0 Da) masses. Our study indeed confirms that glycopeptides displaying such ‘difficult-to-identify’ features (NeuAc, NeuGc, multi-Fuc, Met oxidation, Cys carbamidomethylation) are frequently mis-annotated with current search engines (Extended Data Fig. 5). We therefore recommend that efforts should be invested in improving tools to allow for accurate identification of such challenging glycopeptides.

**Table 3:** Overview of software-independent search variables important for high-performance glycoproteomics data analysis (see Fig. 3b,d, Tables 1 and 2 and Supplementary Table 18 for study variables and associations)

**Performance area**	**Related test**	**High-performance search settings**^**a**^	**High-performance search output (expected)**^**b**^	**Strategy may compromise**
Efficient *N-*glycopeptide analysis (all-round performance)	Overall score (N1–N6)	• Use decoy/contaminant protein database to establish peptide/protein FDR (SS13)	• High *m/z* (SO2) • High monoisotopic correction (SO4) • High glycopeptide mass (SO5) • High glycoPSM count (SO9)	NA
Accurate *N-*glycan identification	N2	NA	• High glycan mass (SO8)	*N-*glycoproteome coverage
Accurate source *N-*glycoprotein identification	N3	NA	• Late LC retention time (SO1) • High glycopeptide mass (SO5)	*N-*glycoproteome coverage
High *N-*glycoproteome coverage	N4	• Allow diversity of variable nonglycan peptide modifications (SS8) • Allow few glycans per peptide (SS9) • Allow multiple variable nonglycan modifications per peptide (SS10) • Use decoy/contaminant protein database to establish peptide/protein FDR (SS13)	• Late LC retention time (SO1) • High charge stage (SO3) • High glycopeptide mass (SO5) • High actual mass error (SO6) • High glycan mass (SO8) • High glycoPSM count (SO9)	*N-*glycopeptide identification accuracy, search time
Reduced NeuGc and multi-Fuc FDR	N6	• Allow few missed peptide cleavages (SS7) • Allow few variable nonglycan modifications per peptide (SS10)	• Low actual mass error (SO6)	*N-*glycoproteome coverage
Efficient *O-*glycopeptide analysis (all-round performance)	Overall score (O1–O5)	• Use broad glycan database (SS2) • Allow few missed peptide cleavages (SS7) • Allow multiple variable nonglycan modifications per peptide (SS10)	• Large peptides (SO7) • High glycoPSM count (SO9)	Search time
Accurate *O-*glycan identification	O1	• Use focused (narrow) glycan database (SS2) • Allow few missed peptide cleavages (SS7)	• High monoisotopic correction (SO4) • Low actual mass error (SO6)	*O-*glycoproteome coverage
Accurate source *O-*glycoprotein identification	O2	• Use broad glycan database (SS2) • Use full trypsin specificity (SS6) • Allow few missed cleavages (SS7) • Allow few glycans per peptide (SS9) • Allow multiple variable nonglycan modifications per peptide (SS10)	• High glycoPSM count (SO9)	*O-*glycoproteome coverage, search time
High *O-*glycoproteome coverage	O3	• Use broad glycan database (SS2)	• High actual mass error (SO6) • High glycoPSM count (SO9)	*O-*glycopeptide identification accuracy, search time

Only search variables closely associated with high performance (≥3 statistical tests) have been included. ^a^Software-independent search settings that may guide improved glycoproteomics search strategies. ^b^Search output expected from high-performance glycoproteomics data analysis. This information may also aid postsearch filtering of glycopeptide data. The possible compromise of selected search strategies on the overall glycoproteomics performance is indicated. NA, not applicable.

Meanwhile, our search engine-centric approach involving systematic Byonic searches revealed search settings impacting the performance of this widely used search engine and highlighted that specificity (accuracy) and sensitivity (coverage) are competing performance characteristics challenging to achieve in a single search. This suggests that glycoproteomics data may benefit from being interrogated using multiple orthogonal search strategies that are subsequently combined or by approaches that strike a balance between accuracy and coverage. To this end, we here recommend a set of improved ‘high accuracy’, ‘high coverage’ and ‘balanced’ search strategies that should be selected (and further tailored/optimized) according to the sample and research question being investigated (Fig. 4f).

Although not a focus here, our study also showed that the search strategy dramatically impacts the search time. While the spectral input type and data output filtering represent other critical variables that also need further exploration, our study indicates that HCD and EThcD are currently the most informative spectral types in glycoproteomics, and that knowledge-guided filtering and curation of data output is critically required to lower FDRs.

The study also highlighted informatics challenges still associated with large-scale glycopeptide data analysis, as illustrated by the discrepant reporting of glycopeptides across teams. Notably, high discordance of reported glycopeptides was even found between participants using the same software, confirming that search variables other than the search engine also substantially impact the glycopeptide data analysis. While the ten Byonic user teams reported a marginally higher rate of consensus glycopeptides, their spread in terms of search output data, reported glycoPSMs and overall performance scores was of similar magnitude as the variance observed among other user teams. While the (self-reported) team experience in glycoproteomics was not found to be an accurate predictor of team scoring and ranking, the variability in the spectral data input, search settings and, importantly, at the postsearch filtering stage were identified as key factors contributing to the discrepant reports. Concertedly, these observations point to the importance of using both the most informative spectral data, powerful search engines, tailored search settings and knowledge-driven postsearch filtering to achieve high-performance glycoproteomics data analysis.

Despite the considerable team-to-team variation, this study produced consensus lists of 163 *N-*glycopeptides and 23 *O*-glycopeptides from serum glycoproteins commonly reported by teams. Importantly, these high-confidence glycopeptides carried biosynthetically related glycans that were devoid of NeuGc and poor in multi-Fuc features in line with the literature^23,40^ and mapped to known high-abundance serum proteins^4,23,39,41,42^. The consensus lists have been made publicly available (GlyConnect ID 2943) as they form an important reference for future studies of the human serum glycoproteome.

The study design including the sample type/preparation and data collection method was chosen to mimic conditions typically encountered in glycoproteomics while also aiming to accommodate most informatic solutions and appeal to users in the field. Multiple orthogonal performance tests and separate validation were applied to ensure a fair and holistic scoring of search engines and teams. Despite these efforts, it cannot be ruled out that some software or users may have been unintentionally disadvantaged and/or excluded by the chosen experimental design and scoring system. The team scoring and ranking should be viewed in light of these constraints and limitations common to most community-based comparison studies founded on communal data.

In addition to reporting on the peptide and glycan components of identified glycopeptides, teams were requested to report on site(s) of modification where possible. As most tryptic *N*-glycopeptides only comprise a single sequon, site localization is primarily a challenge related to *O*-glycoproteomics^5,16^. Most teams indeed returned data of the *O*-glycosylation site(s), but due to highly discrepant and often inconclusive reporting of sites and a paucity of literature on serum *O*-glycosylation sites, we were unable to score glycosylation site localization.

Most software currently available for glycoproteomics data analysis participated in this study. However, several glycopeptide search engines; for example, pGlyco^52^, MSFragger-Glyco^53^, O-Pair Search^54^ and StrucGP^55^, were unfortunately not represented due to LC-MS/MS data incompatibility or due to their development after the study period. Thus, this study is essentially a snapshot of the performance of software available at the time the data analysis was performed. Highlighting the rapid progress in glycoproteome informatics, most of the software solutions participating in this study have been improved and new versions released after the evaluation period. For example, GPQuest v2.0, GlycoPAT v1.0 and Protein Prospector v5.20.23 tested herein have been superseded by more recent versions: namely, GPQuest v2.1, GlycoPAT v2.0 and Protein Prospector v.6.2.2. Thus, a limitation of this study is that newer tools are available at the time of publication that were not compared in our analysis. Follow-up studies comparing the performance of these latest glycoproteomics software upgrades and informatics solutions not included in this study are therefore warranted. Beyond testing the ability of participants to identify the peptide and glycan components of glycopeptides from glycoproteomics data, such future comparative studies should ideally also test the ability to accurately quantify (relative, absolute) and report on modification sites of identified glycopeptides and could explore other relevant parameters not addressed herein including the use of alternative proteases, tandem mass tag-labeling and stepped-HCD-MS/MS data among other experimental conditions gaining popularity in glycoproteomics.

In summary, this community study has documented that the field has several high-performance informatics solutions available for glycoproteomics data analysis and has elucidated key performance-associated search strategies that will serve to guide developers and users of glycoproteomics software.

## Methods

### Study design and participants

Calls to join this study as a developer (academic/commercial) or user of glycoproteomics software were made widely across the proteomics and glycomics community. In total, 25 teams signed up for the study, out of which 22 teams comprising nine developer and 13 user teams completed the study. All teams identified *N-* and *O*-glycopeptides from two communal glycoproteomics LC-MS/MS data files (Files A and B), and reported their findings using a common reporting template (PXD024101, PRIDE repository). While the user teams were guaranteed anonymity, the developers were informed that their software (hence, potentially their identity) would be disclosed on publication. The user teams were free to use any search engine(s) at their disposal including manual annotation/filtering of search output. Developers returned the identified glycopeptides directly from their own software without manual postsearch filtering. The developers employed the following search engines: team 1: IQ-GPA v2.5^30^; team 2: Protein Prospector v5.20.23^31^; team 3: glyXtool^MS^ v0.1.4^32^; team 4: Byonic v2.16.16^33^; team 5: Sugar Qb^34^; team 6: Glycopeptide Search v2.0alpha^35^; team 7: GlycopeptideGraphMS v1.0/Byonic^36^; team 8: GlycoPAT v2.0^37^ and team 9: GPQuest v2.0^38^ (see Supplementary Table 1 and below for overview of software and pre- and postprocessing tools used by all participants). The relative team performance was compared within (not between) the developer and user groups as these two groups were given slightly different instructions (above).

### Synthetic *N*-glycopeptide

An Asn building block carrying a disialylated, biantennary *N*-glycan (Hex_5_HexNAc_4_NeuAc_2_) was purified from chicken egg yolk powder. Previous studies have confirmed that a disialylated, biantennary *N*-glycan carrying only α2,6-linked NeuAc residues is the major component of the chicken egg yolk hexapeptide^56,57^. In short, this glycosylated hexapeptide was subjected to extensive proteolysis to generate a glycosylated Asn, which was then converted into a fluorenylmethoxycarbonyl (Fmoc) protected building block as described earlier^57,58^. Using this glycosylated Asn building block, a synthetic glycopeptide carrying a homogenous *N*-glycan (Hex_5_HexNAc_4_NeuAc_2_) was generated using an established method for solid phase peptide synthesis^58–60^. The synthetic peptide sequence mimicked a tryptic *N*-glycopeptide from human vitamin-K-dependent protein C present in human serum (UniProtKB, P04070, ^284^EVFVHPNYSK^293^). The structure, purity and integrity after deprotection and purification were confirmed using reversed-phase LC-MS/MS as described earlier^59^.

### Study sample

Human serum from a commercial source was used for this study (product no. 31876, Thermo Fisher Scientific). As a positive control, 52 fmol of the synthetic *N*-glycopeptide from human vitamin-K-dependent protein C (see details above) was spiked into 5 µg human serum before digestion. Proteins were cysteine reduced and alkylated before protein digestion using 1:100 (w/w, enzyme:protein substrate) sequence-grade trypsin for 16 h, 37°C in 20 mM aqueous ammonium bicarbonate, pH 8.0. Undigested protein material and large peptides were removed by filtration using a 30 kDa molecular weight cut-off membrane (product no. 88502, Thermo Fisher Scientific). The membrane was washed using 30% (v/v) methanol in 0.1% (v/v) aqueous trifluoroacetic acid (TFA). The flow-through fraction was collected, evaporated using a SpeedVac, and then resuspended in 200 μL 50% (v/v) acetonitrile (ACN) in 0.1% (v/v) aqueous TFA. Glycopeptide enrichment was performed using Hypersep Retain AX columns (product no. 60107-403, Thermo Fisher Scientific). The columns were prepared according to the manufacturer’s instructions and were additionally washed with 100 mM aqueous triethylammonium acetate before equilibration with 95% (v/v) ACN in 1% (v/v) aqueous TFA. The sample was diluted in 3 mL 95% (v/v) ACN in 1% (v/v) aqueous TFA, applied to the columns, and then washed with an additional 3 mL 95% (v/v) ACN in 1% (v/v) aqueous TFA before the glycopeptides were eluted with 1 mL 50% (v/v) ACN in 0.5% (v/v) aqueous TFA. The enriched glycopeptide mixtures were dried using a SpeedVac and resuspended in 0.1% (v/v) aqueous TFA for LC-MS/MS analysis.

### Mass spectrometry

The glycopeptides were separated by reversed-phase nanoLC using a Thermo Scientific EASY-nLC 1200 UPLC system connected to a C_18_ LC column (50 cm length × 75 µm inner diameter, Thermo Scientific EASY-Spray). Separation was achieved using a 75 min 6–45% (v/v) and 3 min 45–95% (v/v) gradient of solvent B consisting of 80% (v/v) ACN in 0.1% (v/v) aqueous formic acid in solvent A consisting of 0.1% (v/v) aqueous formic acid at a 300 nL/min flow rate. The separated glycopeptides were detected using a Thermo Scientific Orbitrap Fusion Lumos Tribrid mass spectrometer connected directly to the LC. Approximately 1 μg peptide material was injected on the LC column per run. The same glycopeptide sample was analyzed twice using two slightly different acquisition methods producing two related data files (Files A and B).

For both methods, MS1 scans were acquired from *m/z* 350–1,800 in the Orbitrap at a resolution of 120,000 and with an automatic gain control (AGC) of 4 × 10^5^ and an injection time of 50 ms. Data-dependent HCD-MS/MS was performed for the ten most intense precursor ions selecting the highest charge state and the lowest *m/z* in each MS1 full scan. The HCD-MS/MS fragment ions were recorded in the Orbitrap at a resolution of 30,000 and with an AGC of 5 × 10^4^, injection time of 60 ms, normalized collision energy (NCE) of 28% and a quadrupole isolation width of 2 Th. Already selected precursors were dynamically excluded for 45 s. Product-dependent ion triggered re-isolation and fragmentation of precursor ions were enabled on detection of at least one of three selected glycan oxonium ions (*m/z* 138.0545, 204.0867 and 366.1396) if the diagnostic ion(s) was among the top 20 fragment ions within each HCD-MS/MS spectrum. For File A, product-dependent-triggered ETciD- and CID-MS/MS events were scheduled. The ETciD-MS/MS fragments were detected in the Orbitrap at a resolution of 60,000 with an AGC of 4 × 10^5^, injection time of 250 ms, CID NCE of 15% and a quadrupole isolation width of 1.6 Th. Charge-dependent ETD calibration was enabled. The CID-MS/MS fragments were detected in the Orbitrap at a resolution of 30,000 with an AGC of 5 × 10^4^, NCE of 30%, injection time of 54 ms and a quadrupole isolation width of 1.6 Th. For File B, product-dependent-triggered EThcD- and CID-MS/MS events were scheduled. The EThcD-MS/MS fragments were detected in the Orbitrap at a resolution of 60,000 with an AGC of 4 × 10^5^, injection time of 250 ms, HCD NCE of 15% and a quadrupole isolation width of 1.6 Th. Charge-dependent ETD calibration was enabled. The CID-MS/MS fragments were detected in the ion trap at unit resolution using a rapid scan method with an AGC of 1 × 10^4^, injection time of 70 ms, NCE of 30% and a quadrupole isolation width of 1.6 Th. Files A and B were provided to all participants as .raw data files (File A: 684 MB, File B: 811 MB) or as three separate .mgf files containing peak lists of the fragment spectra from the three different fragmentation modes used for Files A and B (23.9 MB–65.6 MB). Conversion to .mgf was performed using ProteoWizard^61^.

### Search instructions and reporting template

The participants were requested to use a protein search space provided by the study organizers comprising the entire human proteome (20,201 UniProtKB reviewed sequences, downloaded July 2017) for their search. In contrast to the fixed protein search space, the participants were free to choose the *N-* and *O-*glycan search space. To limit the number of study variables, participants were asked not to include xylose and any glycan substitutions (e.g., phosphate, sulfate and acetylation) in the glycan search space. The participants were requested to report their team details, identification strategy and the identified glycopeptides in a common reporting template organized as five separate sheets in an Excel file comprising the following categories of information: (1) Team and contact details; (2) Identification strategy and other study information; (3) *N-* and *O-*glycan search space; (4) List of identified *N-* and *O-*glycopeptides and (5) Summary of identified glycopeptides and glycoproteins. The returned reports were carefully checked for compliance with the study guideline. See PXD024101 via the PRIDE repository^62^ for the common reporting template and the deidentified reports from all participants forming the foundation of this study.

### Search engines and pre- and postprocessing tools used for the glycopeptide identification

A total of 13 search engines was used for glycopeptide identification: IQ-GPA v2.5^30^, Protein Prospector v5.20.23^31^, glyXtool^MS^ v0.1.4^32^, Byonic v2.16.16^33^, Sugar Qb^34^, Glycopeptide Search v2.0alpha^35^, GlycopeptideGraphMS v1.0/Byonic^36^, GlycoPAT v2.0^37^, GPQuest v2.0^38^, Mascot v2.5.1^63^ or v2.2.07, MS Amanda v1.4.14.8243^64^ and Sequest-HT (in Proteome Discoverer v2.2) (Extended Data Fig. 1h). These tools were used as stand-alone tools or in combinations. Some of the search engines were applied with pre- or postprocessing tools, including OMSSA v2.1.8, Preview v2.13.2, Protein Prospector MS-filter, MS-GF + /PepArML and pParse v.2.0 (Extended Data Fig. 1i).

### Compilation and comparison of participant reports

Information of the participating teams was compiled from the returned reports (Supplementary Table 1–2). The lists of intact *N-* and *O-*glycopeptides reported by the 22 teams were compiled into a single table with a unique header (Supplementary Table 3). Additional columns were manually added to the compiled table with the purpose of standardizing some of the reported text variables and generating unique identifiers (IDs) for the reported glycopeptides and their glycan compositions and source glycoproteins. The glycan composition ID was written as the generic monosaccharide composition as Hex*HexNAc*Fuc*NeuAc*, where * represents the number of the individual monosaccharide residues. Glycopeptides adducted with Na^+^ and K^+^ were considered and reported by some teams. The adducted glycopeptides were combined with the corresponding nonadducted monosaccharide compositions. UniProtKB IDs were used as the source protein IDs. The glycopeptide IDs were written as the peptide sequence followed by the generic glycan composition.

The comparisons between the generic glycan compositions, source proteins and glycopeptide IDs reported by the 22 teams were performed using the pivot table tool available in Excel, where the ID type was placed in ‘rows’, and the team ID in ‘columns’. The variables from each ID type were compared as summed counts across the 22 teams.

### Performance testing of teams and software

The relative team and software performance for glycopeptide data analysis was in this study determined via three different methods as detailed below. In short, all teams were first scored and ranked based on a comprehensive assessment method involving multiple complementary performance tests (1). Subsequently, the scoring of teams was validated using an independent glycoprotein-based assessment score (2). Finally, for the search engine-centric analysis and optimization of the search strategies for Byonic, the relative performance was evaluated based on a scoring method that produced relative specificity and sensitive scores (3).

#### Scoring and ranking of teams via multiple performance tests (N1–N6 and O1–O5)

The relative team performance was assessed using a scoring system composed of multiple independent tests designed to score the accuracy (specificity) and coverage (sensitivity) of the reported *N-* and *O-*glycopeptides in orthogonal ways. The raw scores from the individual tests (N1–N6 and O1–O5, described below) were normalized within the range 0–1. These normalized scores were used to establish an overall performance score (range 0–1), measuring the ability to perform accurate and comprehensive *N-* and *O*-glycopeptide analysis. The overall performance score was utilized to separately rank the developer and user teams.

- The synthetic *N-*glycopeptide test (N1): All MS/MS spectra corresponding to the synthetic *N-*glycopeptide from human vitamin K-dependent protein C (peptide sequence: EVFVHPNYSK, glycan composition: HexNAc_4_Hex_5_NeuAc_2_) were manually retrieved and annotated from Files A- and B. In total, nine MS/MS spectra corresponded to the nonadducted synthetic *N-*glycopeptide in charge state 3^+^ and 4^+^ spanning the four applied fragmentation modes (HCD-, ETciD-, EThcD- and CID-MS/MS) (Extended Data Fig. 8a,b). A further three MS/MS spectra (HCD-, EThcD- and CID-MS/MS) corresponded to the K^+^-adducted synthetic *N*-glycopeptide in charge state 5^+^. The sensitivity of the test was determined as the proportion of the 12 MS/MS spectra mapping to the synthetic *N-*glycopeptide that was reported by each team adjusting for the type of fragmentation mode(s) included in their respective search strategies. The specificity was calculated by the proportion of correctly reported glycoPSMs corresponding to the synthetic glycopeptide that matched the 12 annotated MS/MS spectra, again adjusting for the type of fragmentation mode(s) included in the applied search strategies. The test score was calculated by multiplying the sensitivity and specificity (Extended Data Fig. 8c,d and Supplementary Table 5).
- The glycan composition test (N2 and O1). The *N-*glycan composition score was calculated based on the Pearson correlation (*R*^2^) between the expected distribution of *N-*glycans carried by human serum glycoproteins as reported by Clerc et al^23^ and the observed *N-*glycan distribution reported by each team. The *O-*glycan composition score was calculated based on the Pearson correlation (*R*^2^) between the expected distribution of *O-*glycans carried by human serum glycoproteins as reported by Yabu et al^40^ and the observed *O-*glycan distribution reported by each team. The distribution of the *N-* and *O-*glycan compositions was calculated based on the glycoPSM count of each unique glycan ID relative to the total glycoPSM count reported by each team.
- The source glycoprotein test (N3 and O2). The source glycoprotein score was determined from the accuracy (specificity) and coverage (sensitivity) of the reported source glycoproteins relative to the glycoproteins expected in human serum. Reported *N-*glycoproteins previously identified in human serum by both Clerc et al^23^ and Sun et al^39^ received a score of 2, whereas *N-*glycoproteins only identified by Sun et al received a score of 1. Source glycoproteins not identified by any of the two studies received no score. Further, reported *O*-glycoproteins previously identified in human serum by Darula et al^41^, Yang et al^42^ and Ye et al^4^ received a score of 3, 2 or 1 according to the number of papers identifying the specific *O*-glycoprotein. The source glycoproteins not reported by any of these three studies received no score. For both the serum *N-* and *O*-glycoproteins, the number of glycoPSMs reported by each team was multiplied by the respective source glycoprotein score for each unique glycoprotein ID. The specificity of the test was calculated based on the summed glycoprotein score divided by the highest possible total score (number of unique glycoproteins reported by each team multiplied by the highest theoretical glycoprotein score). The sensitivity of the test was calculated based on the summed number of glycoproteins with score >0 divided by the number of unique source glycoproteins reported in the selected literature.
- The glycoproteome coverage test (N4 and O3): The *N-* and *O-*glycoproteome coverage was calculated based on the number of unique glycopeptides (unique peptide sequence and glycan composition) reported by each team.
- The commonly reported (‘consensus’) glycopeptide test (N5 and O4): The consensus *N-*glycopeptide score was calculated based on the proportion of glycopeptides commonly reported by at least 50% of the 22 teams returning *N-*glycopeptide data. The consensus *O-*glycopeptide score was calculated based on the number of glycopeptides commonly reported by at least 30% of the 20 teams returning *O-*glycopeptide data.
- The NeuGc and multi-Fuc glycopeptide test (N6 and O5). The number of reported *N-* and *O*-glycoPSMs corresponding to NeuGc and multi-Fuc (Fuc ≥2) containing glycopeptides was normalized to the total glycoPSMs reported by each team. Separate *N-* and *O-*glycopeptide scores were then calculated based on the average of non-NeuGc and non-Fuc ≥2 containing glycoPSMs for teams that included NeuGc and multi-Fuc containing glycan compositions in their glycan search space.

The overall performance scores for *N-* and *O-*glycopeptide analysis were established separately by averaging the scores of the individual performance tests (N1–N6 and O1–O5, respectively).

#### Orthogonal glycoprotein-based scoring to validate the team scoring and ranking

To validate the scoring and ranking of teams based on the multiple performance tests described above (“Scoring and ranking of teams via multiple performance tests (N1–N6 and O1–O5)”), an orthogonal glycoprotein-centric scoring method was devised. The method, founded on a ‘ground truth’ as opposed to inference from the literature, evaluated the quantitative match of the glycoPSMs reported by the teams to the actual site-specific *N*-glycosylation of selected high-abundance glycoproteins including A1AT, CP, HP and IgG1. For this purpose, two metrics were developed (specificity and sensitivity) to score the match to the actual site-specific glycoform distribution. First, the site-specific distribution of *N*-glycans covering Asn70, Asn107 and Asn271 from A1T1, Asn138, Asn358 and Asn762 from CP, Asn184 and Asn241 from HP and Asn180 from IgG1 was manually determined using area-under-the-curve (AUC)-based glycopeptide quantitation (see below for details) and also determined for each team based on spectral counting of reported glycoPSMs. The specificity score was then calculated by multiplying the site-specific glycoform distributions reported by teams by the relative abundance of the actual site-specific glycoforms. The site-glycoform specificity scores were summed within each protein and normalized across the teams (best coverage set to 1). The ‘overall specificity score’ was calculated by averaging the normalized scores from A1AT, CP, HP and IgG1. The sensitivity score was calculated by the proportion of reported nonredundant (unique) glycoforms covering the expected site-specific glycoforms of the four glycoproteins based on robust literature^23,43–46^. The site-glycoform sensitivity scores were summed within each protein and normalized across the teams (best coverage set to 1). The ‘overall sensitivity score’ was determined by averaging the normalized scores from A1AT, CP, HP and IgG1. Combined scores (‘glycoprotein-centric score’) were established by averaging the overall specificity scores and the overall sensitivity scores. The combined scores were then compared to the overall *N*-glycopeptide scores generated from the performance tests N1–N6 (see “Scoring and ranking of teams via multiple performance tests (N1–N6 and O1–O5)” above) using Pearson correlation (*R*^2^). The data underpinning this scoring method can be found in Supplementary Table 17.

#### Search engine-centric scoring of the sensitivity and specificity of Byonic search strategies

To establish the performance of various Byonic search strategies, a scoring method that assessed the relative sensitivity (coverage) and specificity (accuracy) of the search engine was devised. For this purpose, the multiple performance tests already established for the scoring and ranking of teams (N1–N6 and O1–O5; see “Scoring and ranking of teams via multiple performance tests (N1–N6 and O1–O5)”) were used, but in a slightly different manner. The individual sensitivity and specificity scores from the synthetic *N*-glycopeptide test (N1) and source glycoprotein test (N3 and O2) were namely separately considered and grouped with several sensitivity and specificity-centric performance tests to establish ‘global sensitivity scores’ and ‘global specificity scores’ that could be compared between searches. The global sensitivity score for *N*-glycopeptides was determined by averaging the normalized sensitivity scores from the synthetic glycopeptide (N1), source *N*-glycoprotein (N3), *N*-glycoproteome coverage (N4) and commonly reported (‘consensus’) *N*-glycopeptide (N5) tests. The global specificity for *N*-glycopeptides was determined by averaging the normalized specificity score from the synthetic *N*-glycopeptide (N1), glycan composition (N2), source *N*-glycoprotein (N3) and non-NeuGc/multi-Fuc glycopeptide (N6) tests. The global sensitivity score for *O*-glycopeptides was determined by averaging the normalized sensitivity score from source *O*-glycoprotein (O2), *O*-glycoproteome coverage (O3) and commonly reported (‘consensus’) *O*-glycopeptide (O4) tests. The global specificity for *O*-glycopeptides was determined by averaging the normalized specificity score from the *O*-glycan composition (O1), source *O*-glycoprotein (O2) and non-NeuGc/multi-Fuc glycopeptide (O5) tests.

### Manual quantitative glycoprofiling of select serum *N*-glycoproteins

A comprehensive quantitative site-specific analysis of the *N*-glycosylation of four high-abundance serum *N*-glycoproteins including α-1-antitrypsin (A1AT, UniProtKB, P01009, three *N*-glycosylation sites: Asn70, Asn107 and Asn271), ceruloplasmin (CP, P00450, Asn138, Asn358 and Asn762), haptoglobin (HP, P00738, Asn184 and Asn241) and immunoglobulin G1 (IgG1, P01857, Asn180) was manually performed to allow for a quantitative comparison of the studied glycoprotein sample to glycoprofiling data in the literature^43–46^, and thus validate the literature-based performance tests (N2–N3 and O1–O2) used to score teams (above). The site-specific *N*-glycoprofiling data were also used as a ‘ground truth’ to validate the scoring and ranking of teams in an orthogonal manner (see above for details).

For the quantitative site-specific glycoprofiling, the HCD- and EThcD-MS/MS data from File B were first searched using Byos v3.9–7 (Protein Metrics Inc.)^33,65^. The ‘default’ search strategy for *N*-glycopeptides commonly used by teams in this study was employed for the Byos search (see details below). The Byos-identified *N*-glycopeptides (PEP-2D <0.001 was used as a general confidence threshold) were manually confirmed, and the Byos output and the LC-MS/MS raw data were carefully inspected for any additional *N*-glycoforms expected based on the literature of the selected glycoproteins^43–46^, or based on known biosynthetic rules using Xcalibur v3.0.63 (Thermo Fisher Scientific) and with support from protein sequence handling software GPMAW v9.51 (Lighthouse)^66^. This comprehensive approach ensured that all relevant *N*-glycopeptides belonging to these four source glycoproteins were included in the quantitative analysis. The relative abundance of all observed *N*-glycopeptides from the four selected source glycoproteins was manually determined using EIC-based area-under-the-curve measurements of all observed charge states of the monoisotopic precursor ions using Xcalibur v3.0.63 (Thermo Fisher Scientific). The relative abundance of each glycoform was determined as the percentage of the peak intensity of the individual glycopeptide forms relative to the peak intensity of all glycopeptides spanning each glycosylation site, an approach commonly employed in quantitative glycopeptide analysis^67–69^.

### Analysis and optimization of the search strategies used for the Byonic search engine

A Byonic-centric analysis and optimization of the search strategies were performed through a series of controlled in-house searches in which the search settings were systematically varied and the output assessed for performance. For this purpose, only the HCD- and EThcD-MS/MS data from File B were used and searched on an ordinary desktop computer (Windows 10, 64-bit, 16 GB RAM, Intel Core i7-8700 at 3.20 GHz). The ‘heavy’ multicore parameter option was selected for all searches. Fragment spectra from these two dissociation methods were searched in concert (‘HCD/EThcD’ setting enabled) using Byonic v3.9.4 (Protein Metrics Inc.) using a series of search strategies in which the diverse search settings were sequentially changed. The search strategy used by most teams employing Byonic in this study is herein referred to as the ‘default’ search strategy. The default search strategy employed a predefined glycan database containing either 309 mammalian *N*-glycans or 78 mammalian *O*-glycans available within Byonic, allowed up to one glycan per peptide as a ‘rare’ variable modification, considered only peptides with tryptic cleavage patterns with a maximum of two missed tryptic cleavages per peptide, allowed up to 10/20 ppm deviation of the observed precursor/product ion masses from the expected values, considered up to one Met oxidation (+15.994 Da) per peptide (variable ‘common’ modification), used monoisotopic correction (error check equals ± floor (mass in Da/4,000)) and employed a decoy and contaminant database available in Byonic. One or more of the search settings (SS1–SS2 and SS6–SS14) used for the default search strategy were then systematically changed; these alternative settings were selected based on the literature and by taking inspiration from search strategies used by the high-performance teams. For the *N-* and *O*-glycan databases (SS1–SS2), customized glycan databases of 25 *N*-glycans expected in human serum (Clerc et al^23^) or 13 *O*-glycans expected in human serum (Yabu et al^40^) were used. The systematic searches also explored the output when allowing up to two glycans per peptide as a ‘rare’ variable modification (SS9), when considering semispecific trypsin cleavages (SS6) with a maximum of one missed cleavage per peptide (SS7), when allowing 5/10 ppm deviation of the observed precursor/product ion masses to their expected values (SS11/SS12), when considering up to four variable ‘common 1’ modifications per peptide, including Met oxidation (+15.994 Da), Asn/Gln deamidation (+0.9840 Da), Gln → pyro Glu (−17.0265 Da) (SS8 and SS10) and, finally, also when no error check for monoisotopic correction (SS14) and no decoy/contaminant database (SS13) were employed. Cys carbamidomethylation (+57.021 Da) (fixed modification) and the protein search space (20,201 UniProtKB reviewed sequences, downloaded July 2017) remained constant across all searches and none of the searches employed spectral recalibration. Glycopeptides were filtered to 0% FDR at the peptide level by manually removing glycopeptides identified in the decoy or contaminant database after a general confidence score threshold was applied to the data output (Byonic score >100). The resulting lists of glycopeptides identified from each of these Byonic-centric searches were subjected to the devised performance tests for *N-* and *O*-glycopeptides (N1–N6 and O1–O5, respectively) and the relative sensitivity and specificity scores were determined as described above. All sensitivity and specificity scores were normalized to the scores arising from the default search strategy (set to 1). A detailed summary of the search settings and performance scores generated from these systematic searches can be found in Supplementary Table 19a.

In addition to the analysis and optimization of the search settings used for Byonic, we analyzed the impact of the data input on the performance of this search engine. For this purpose, series of controlled searches were carried out by systematically changing the fragmentation type considered for the searches while keeping the search settings and data output filtering constant. The File B raw data file was used as input for all searches and the fragmentation type for each search was specified within the Byonic interface. The ‘default’ search strategy for *N*-glycopeptides was used for all searches (see above for details). Fragment mass tolerance of 0.5 Da and 20 ppm were considered for CID- and HCD/EThcD-MS/MS data, respectively. The following fragmentation types and combinations thereof were tested in individual searches: CID only, HCD only, EThcD only, HCD/EThcD (in concert), HCD/CID (in concert) and HCD/EThcD/CID (in concert). Glycopeptides were filtered using the same criteria described above. Sensitivity and specificity scores were determined for the identified *N*-glycopeptides from each of the searches. Data from these additional searches can be found in Supplementary Table 19c.

### Statistical analysis

Scores from each performance test (N1–N6 and O1–O5) and the overall team performance scores were tested for associations with the search settings (SS1–SS13) and search outputs (SO1–SO9) (average of selected variables). Specifically, seven statistical methods were applied to identify search settings and search output characteristics that were associated with high performance scores including: (1) a multiple linear regression model applied with a significance threshold of *P* < 0.05 to identify association between search variables (predictors) and performances scores (response variable); (2) a ridge linear regression model applied using an induced smoothing paradigm for hypothesis testing^70^; (3) a Lasso linear model for variable selection^71^; (4) a least angle regression exploiting exact postselection inference to identify associations^72,73^; (5) a forward stepwise linear regression applied using selective inference to identify association^74^; (6) a random forest algorithm (an ensemble learning model for regression) applied using a variable of importance score to identify association^75^ (a permutation strategy on augmented set of noise variables was exploited to define the variable importance cut-off); and (7) a gradient boosting tree algorithm (an ensemble of decision trees for prediction) applied using a similar strategy as the random forest algorithm to select important associations^76,77^. R packages v.1.2, 1.2.5 and 2.1.8 were used for these analyses. Only associations commonly observed across a minimum of three different statistical methods were considered in this study.

Unpaired two-sided *t*-tests were applied to compare *N*-glycopeptide (*n* = 22) against *O*-glycopeptide (*n* = 20) search output data from all teams (Extended Data Fig. 3) and to compare the performance scores based on HCD-MS/MS data (*n* = 17 or *n* = 16) against EThcD-MS/MS data (*n* = 13 or *n* = 10) (Extended Data Fig. 9). The confidence interval was set to 95% and statistical significance was indicated as *P < 0.05, **P < 0.01 and ***P < 0.001.

Pearson correlations (*R*^2^) were used to determine (1) the quantitative match between the observed site-specific *N*-glycan distribution of four selected glycoproteins in the investigated sample and the site-specific glycoform distribution reported by the literature and (2) the similarity between the overall team scores and the glycoprotein-based scores for all 22 teams.

### Reporting Summary

Further information on research design is available in the Nature Research Reporting Summary linked to this article.

## Online content

Any methods, additional references, *Nature Research* reporting summaries, source data, extended data, supplementary information, acknowledgements, peer review information; details of author contributions and competing interests; and statements of data and code availability are available at 10.1038/s41592-021-01309-x.

## Supplementary information

Reporting Summary

Supplementary Tables 1–19 Supplementary Table 1: Overview of the study participants and their reported data. Supplementary Table 2: The *N-* and *O*-glycan search space applied by teams. Supplementary Table 3: Identified *N-* and *O*-glycopeptides and other search output. Supplementary Table 4: Overview of quantitative search output reported by teams. Supplementary Table 5: The synthetic *N*-glycopeptide performance test (N1). Supplementary Table 6: The *N*-glycan composition performance test (N2). Supplementary Table 7: The source *N*-glycoprotein performance test (N3). Supplementary Table 8: The *N*-glycoproteome coverage performance test (N4). Supplementary Table 9: The commonly reported (consensus) *N*-glycopeptide performance test (N5). Supplementary Table 10: The NeuGc/multi-Fuc *N*-glycopeptide performance test (N6). Supplementary Table 11: The *O*-glycan composition performance test (O1). Supplementary Table 12: The source *O*-protein performance test (O2). Supplementary Table 13: The *O*-glycoproteome coverage performance test (O3). Supplementary Table 14: The commonly reported (consensus) *O*-glycopeptide performance test (O4). Supplementary Table 15: The NeuGc/multi-Fuc *O*-glycopeptide performance test (O5). Supplementary Table 16: Summary of team performance, search settings and search output. Supplementary Table 17: Glycoprotein-based scoring to validate team scoring and ranking. Supplementary Table 18: Performance-associated search variables (team-centric analysis). Supplementary Table 19: Search engine-centric analysis of Byonic performance.

Peer review information

## Data Availability

Figures 1–4, Tables 1–3 and Extended Data Figs. 1–4, 8–10 have associated raw data. The supporting information includes Extended Data Figs. 1–10 and Supplementary Tables 1–19 (Microsoft Excel). The LC-MS/MS raw data (Files A and B), reporting template and deidentified but otherwise unredacted team reports are available via ProteomeXchange (PXD024101). The consensus glycopeptides are available via the GlyConnect resource of the Glycomics@ExPASy collection hosted at SIB (the Swiss Institute of Bioinformatics) (GlyConnect Reference ID 2943).

## Extended data

are available for this paper at 10.1038/s41592-021-01309-x.

## Code availability

Developers (teams 1–9) used their own software to complete this study. All participants including the developers detailed how software were handled and how data were generated. Additionally, developers detailed in their reports how their software could be tested and data validated by the study committee. While the codes for the developer software (commercial/academic origins) have not been released as part of this work, all team reports underpinning this study have been released (Data Availability Statement).
