# Author Correction: Community evaluation of glycoproteomics informatics solutions reveals high-performance search strategies for serum glycopeptide analysis

**DOI:** 10.1038/s41592-021-01368-0

**Published:** 2021-12-10

**Authors:** Rebeca Kawahara, Anastasia Chernykh, Kathirvel Alagesan, Marshall Bern, Weiqian Cao, Robert J. Chalkley, Kai Cheng, Matthew S. Choo, Nathan Edwards, Radoslav Goldman, Marcus Hoffmann, Yingwei Hu, Yifan Huang, Jin Young Kim, Doron Kletter, Benoit Liquet, Mingqi Liu, Yehia Mechref, Bo Meng, Sriram Neelamegham, Terry Nguyen-Khuong, Jonas Nilsson, Adam Pap, Gun Wook Park, Benjamin L. Parker, Cassandra L. Pegg, Josef M. Penninger, Toan K. Phung, Markus Pioch, Erdmann Rapp, Enes Sakalli, Miloslav Sanda, Benjamin L. Schulz, Nichollas E. Scott, Georgy Sofronov, Johannes Stadlmann, Sergey Y. Vakhrushev, Christina M. Woo, Hung-Yi Wu, Pengyuan Yang, Wantao Ying, Hui Zhang, Yong Zhang, Jingfu Zhao, Joseph Zaia, Stuart M. Haslam, Giuseppe Palmisano, Jong Shin Yoo, Göran Larson, Kai-Hooi Khoo, Katalin F. Medzihradszky, Daniel Kolarich, Nicolle H. Packer, Morten Thaysen-Andersen

**Affiliations:** 1 Department of Molecular Sciences, Macquarie University, Sydney, NSW Australia; 2 Institute for Glycomics, Griffith University Gold Coast Campus, Southport, QLD Australia; 3 Protein Metrics Inc., Cupertino, CA USA; 4 Institutes of Biomedical Sciences, and the NHC Key Laboratory of Glycoconjugates Research, Fudan University, Shanghai, China; 5 UCSF, School of Pharmacy, Department of Pharmaceutical Chemistry, San Francisco, CA USA; 6 State University of New York, Buffalo, NY USA; 7 Analytics Group, Bioprocessing Technology Institute, Agency for Science, Technology and Research, Singapore, Singapore; 8 Clinical and Translational Glycoscience Research Center (CTGRC), Georgetown University, Washington, DC USA; 9 Department of Biochemistry and Molecular & Cellular Biology, Georgetown University, Washington, DC USA; 10 Department of Oncology, Georgetown University, Washington, DC USA; 11 Max Planck Institute for Dynamics of Complex Technical Systems, Bioprocess Engineering, Magdeburg, Germany; 12 Department of Pathology, The Johns Hopkins University, Baltimore, MD USA; 13 Department of Chemistry and Biochemistry, Texas Tech University, Lubbock, TX USA; 14 Research Center of Bioconvergence Analysis, Korea Basic Science Institute, Daejeon, Republic of Korea; 15 Department of Mathematics and Statistics, Macquarie University, Sydney, NSW Australia; 16 CNRS, Laboratoire de Mathématiques et de leurs Applications de PAU, E2S-UPPA, Pau, France; 17 State Key Laboratory of Proteomics, Beijing Institute of Lifeomics, Beijing Proteome Research Center, National Center for Protein Sciences (Beijing), Beijing, China; 18 Proteomics Core Facility, Sahlgrenska academy, University of Gothenburg, Gothenburg, Sweden; 19 BRC, Laboratory of Proteomics Research, Szeged, Hungary; 20 Doctoral School in Biology, Faculty of Science and Informatics, University of Szeged, Szeged, Hungary; 21 Department of Anatomy and Physiology, University of Melbourne, Melbourne, VIC Australia; 22 School of Chemistry and Molecular Biosciences, University of Queensland, Queensland, QLD Australia; 23 IMBA, Institute of Molecular Biotechnology of the Austrian Academy of Sciences, Vienna, Austria; 24 Department of Medical Genetics, Life Sciences Institute, University of British Columbia, Vancouver, BC Canada; 25 glyXera GmbH, Magdeburg, Germany; 26 Deparment of Microbiology and Immunology, University of Melbourne, Melbourne, VIC Australia; 27 Copenhagen Center for Glycomics, Department of Cellular and Molecular Medicine, University of Copenhagen, Copenhagen, Denmark; 28 Department of Chemistry and Chemical Biology, Harvard University, Cambridge, MA USA; 29 Department of Biochemistry, Boston University Medical Campus, Boston, MA USA; 30 Department of Life Sciences, Imperial College London, London, UK; 31 Instituto de Ciências Biomédicas, Departamento de Parasitologia, Universidade de São Paulo, São Paulo, SP Brazil; 32 Graduate School of Analytical Science and Technology, Chungnam National University, Daejeon, Republic of Korea; 33 Department of Laboratory Medicine, Sahlgrenska Academy, University of Gothenburg, Gothenburg, Sweden; 34 Institute of Biological Chemistry, Academia Sinica, Taipei, Taiwan; 35 Biomolecular Discovery Research Centre, Macquarie University, Sydney, NSW Australia

**Keywords:** Computational platforms and environments, Research data, Software, Glycobiology

Correction to: *Nature Methods* 10.1038/s41592-021-01309-x, published online 1 November 2021.

In the version of this article initially published, extraneous references 29, 44–47, 19–22 were listed in Extended Data Fig. 4 caption and ref. 48 was listed in Extended Data Fig. 7 caption. The references have been removed from the HTML and PDF versions of the article.

