# Supplementary material for: Community evaluation of glycoproteomics informatics solutions reveals high-performance search strategies for serum glycopeptide analysis
Source: Nat Methods. 2021 Nov 1;18(11):1304–16. doi: 10.1038/s41592-021-01309-x (PMC8566223; doi:10.1038/s41592-021-01309-x)
Supplement: Supplementary file 1 — Reporting Summary [file 41592_2021_1309_MOESM1_ESM.pdf]

nature research

Corresponding author(s): Dr Morten Thaysen-Andersen

Last updated by author(s): Sep 19, 2021

Reporting Summary

Nature Research wishes to improve the reproducibility of the work that we publish. This form provides structure for consistency and transparency in reporting. For further information on Nature Research policies, see our Editorial Policies and the Editorial Policy Checklist.

Statistics

For all statistical analyses, confirm that the following items are present in the figure legend, table legend, main text, or Methods section.

n/a	Confirmed	
☐	☒	The exact sample size (*n*) for each experimental group/condition, given as a discrete number and unit of measurement
☐	☒	A statement on whether measurements were taken from distinct samples or whether the same sample was measured repeatedly
☐	☒	The statistical test(s) used AND whether they are one- or two-sided<br>*Only common tests should be described solely by name; describe more complex techniques in the Methods section.*
☐	☒	A description of all covariates tested
☐	☒	A description of any assumptions or corrections, such as tests of normality and adjustment for multiple comparisons
☐	☒	A full description of the statistical parameters including central tendency (e.g. means) or other basic estimates (e.g. regression coefficient) AND variation (e.g. standard deviation) or associated estimates of uncertainty (e.g. confidence intervals)
☐	☒	For null hypothesis testing, the test statistic (e.g. *F*, *t*, *r*) with confidence intervals, effect sizes, degrees of freedom and *P* value noted<br>*Give P values as exact values whenever suitable.*
☒	☐	For Bayesian analysis, information on the choice of priors and Markov chain Monte Carlo settings
☒	☐	For hierarchical and complex designs, identification of the appropriate level for tests and full reporting of outcomes
☐	☒	Estimates of effect sizes (e.g. Cohen's *d*, Pearson's *r*), indicating how they were calculated

Our web collection on statistics for biologists contains articles on many of the points above.

Software and code

Policy information about availability of computer code

Data collection

As outlined in the manuscript (see for example "study design and overview" pp.7-12), our study relied on data collected by many study participants using shared LC-MS/MS data files. The participants were free to use any bioinformatics solutions available to them (see overview of the used software below; this information has been provided in the Online Methods in the manuscript p.37). All participants provided a detailed report of how the software was handled and how the reported data were generated. In addition, the software developers were asked to explain in their reports how their software could be tested and data validated by the study committee. While the codes for the developer software (commercial and academic origin) have not been released as part of this work, all the team reports forming the foundation of this comparison study have been made publicly available. This information has been provided in a "Code Availability Statement" in the manuscript pp.52-53.

-IQ-GPA v2.5 (type: in-house, available in https://www.igpa.kr/)
-Protein Prospector v5.20.233 (type: academic, open source, available in https://prospector.ucsf.edu/prospector/mshome.htm)
-glyXtoolMS v0.1.4 (type: open source, available in https://github.com/glyXera/glyXtoolMS)
-Byonic v2.16.16 (type: commercial, available in https://proteinmetrics.com/)
-Sugar Qb (v. 20/09/2017 for N-glycans, type: in-house, available in www.imba.oeaw.ac.at/sugarqb)
-Glycopeptide Search v2.0alpha (type: academic/in-house, available in http://edwardslab.bmcb.georgetown.edu/GPS)
-GlycopeptideGraphMS v1.0 (type: academic, available in https://bitbucket.org/glycoaddict/glycopeptidegraphms/src/master/
-GlycoPAT v2.0 (type: academic, available in https://virtualglycome.org/glycopat)
-GPQuest v2.0 (type: academic)
-Mascot v2.5.1 (type: commercial, available in https://www.matrixscience.com/)
-Sequest HT/Proteome discoverer v.2.2 (type: commercial, available in https://www.thermofisher.com/au/en/home.html)

Other software/algorithms/packages/versions used in this study:
Microsoft excel version 2107
Byos v3.9-7 (Protein Metrics Inc., CA, USA)

GPMAW v9.51 (Lighthouse, Odense, Denmark)
Byonic v3.9.4
Xcalibur v3.0.63 (Thermo Fisher Scientific)
Random Forest algorithm
Gradient boosting tree algorithm
R package v1.2, 1.2.5 and 2.1.8

Other software versions mentioned in the manuscript but not used in the analysis:
GPQuest v2.1
GlycoPAT v2.0
Protein Prospector v.6.2.2

Data analysis

An independent study committee performed all the analysis of data collected and reported by the study participants using Microsoft Excel and statistical tools as described in the Online Methods (p.51).

For manuscripts utilizing custom algorithms or software that are central to the research but not yet described in published literature, software must be made available to editors and reviewers. We strongly encourage code deposition in a community repository (e.g. GitHub). See the Nature Research guidelines for submitting code & software for further information.

Data

Policy information about availability of data

All manuscripts must include a data availability statement. This statement should provide the following information, where applicable:

- Accession codes, unique identifiers, or web links for publicly available datasets
- A list of figures that have associated raw data
- A description of any restrictions on data availability

We have included a "Data Availability Statement" section that as outlined below describes the data availability (p.52).

"This study contains extended data figures and other supplementary information. Multiple figures/tables have associated raw data (Figure 1-4, Table 1-3, Extended Data Figure 1-4, 8-10). The supporting information includes: 1) Extended Data Figure 1-10, and 2) Supplementary Table 1–19 (Microsoft Excel). Further, the LC-MS/MS raw data (File A-B), reporting template, and deidentified but otherwise unredacted team reports are available via ProteomeXchange (PXD024101). Username: reviewer_pxd024101@ebi.ac.uk, Password: YLk2wW1P. The consensus glycopeptides are available via the GlyConnect resource of the Glycomics@ExPASy collection hosted at SIB - Swiss Institute of Bioinformatics (GlyConnect Reference ID 2943)."

Field-specific reporting

Please select the one below that is the best fit for your research. If you are not sure, read the appropriate sections before making your selection.

☒ Life sciences ☐ Behavioural & social sciences ☐ Ecological, evolutionary & environmental sciences

For a reference copy of the document with all sections, see nature.com/documents/nr-reporting-summary-flat.pdf

Life sciences study design

All studies must disclose on these points even when the disclosure is negative.

Sample size

The sample size of this inter-laboratory comparison study could be considered as the number of teams (n = 22) completing the study. The sample size of developer teams was n = 9 and expert user teams was n = 13. All 22 teams reported N-glycopeptide data while 20 teams reported O-glycopeptide data for File B that was used for the detailed data and correlation analyses. The sample sizes have been clearly mentioned in multiple sections of the manuscript in relevant places (e.g. p.8). No sample size calculation was performed. Since no biological conclusion was drawn, sample size is sufficient.

Data exclusions

File B was handled by all participants and thus used for the extensive data analysis and correlation testing to identify high performance search strategies and software solutions. While not being excluded from the reporting, File A that was only handled by a subset of the participants was not used for the downstream data and correlation analyses except for performance test N1 (synthetic N-glycopeptide test) due to relatively weak statistical power related to data reported from this dataset. These important points have been clearly mentioned in multiple sections of the manuscript (e.g. p.8).

Replication

As described in the manuscript, two LC-MS/MS data files (File A-B) of the same biological sample were shared with and analysed by participants of the study - the two data files were acquired using slightly different mass spectrometry methods to cater for as many software developers and users as possible in the community. As such no conventional biological or technical replicates were included in the experimental design. However, since the participants all analysed the same data, these may be considered as technical replicates of the data analysis process.

Randomization

Randomisation was not included in the experimental design due to the nature of the study. Biological replicates were not included in this study, thus randomization was not needed.

Blinding

The data collected by the participants were analysed by an independent study committee that did not have any conflict of interest in the study outcomes (described on p.24). No blinding of the reported data collected by the participants was performed, but the participant reports were deidentified in accordance with the study guidelines (established at the conception of the study) before the reports were made publicly

available upon manuscript submission. Biological replicates were not included in this study, thus blinding was not needed.

Reporting for specific materials, systems and methods

We require information from authors about some types of materials, experimental systems and methods used in many studies. Here, indicate whether each material, system or method listed is relevant to your study. If you are not sure if a list item applies to your research, read the appropriate section before selecting a response.

Materials & experimental systems

n/a	Involved in the study
☒	☐ Antibodies
☒	☐ Eukaryotic cell lines
☒	☐ Palaeontology and archaeology
☒	☐ Animals and other organisms
☒	☐ Human research participants
☒	☐ Clinical data
☒	☐ Dual use research of concern

Methods

n/a	Involved in the study
☒	☐ ChIP-seq
☒	☐ Flow cytometry
☒	☐ MRI-based neuroimaging
