# Supplementary material for: Community evaluation of glycoproteomics informatics solutions reveals high-performance search strategies for serum glycopeptide analysis
Source: Nat Methods. 2021 Nov 1;18(11):1304–16. doi: 10.1038/s41592-021-01309-x (PMC8566223; doi:10.1038/s41592-021-01309-x)
Supplement: Supplementary file 3 — Peer review information [file 41592_2021_1309_MOESM3_ESM.pdf]

natureresearch

Peer Review Information

Journal: Nature Methods
Manuscript Title: Community Evaluation of Glycoproteomics Informatics Solutions Reveals High-Performance Search Strategies of Serum *N*- and *O*-Glycopeptide Data
Corresponding author name(s): Morten Thaysen-Andersen

Reviewer Comments & Decisions:

Decision Letter, initial version:

Dear Dr. Thaysen-Andersen,

We have now received the reviews of your manuscript (NMETH-AS45546, "Community Evaluation of Glycoproteomics Informatics Solutions Reveals High-Performance Search Strategies of Glycopeptide Data") here at Nature Methods. Some serious issues were raised by the referees. Could you please send me an e-mail that delineates in a point-by point fashion how you would rectify these concerns if given the opportunity to revise your manuscript? This will help us as editors to make a more informed decision on your manuscript.

We realize that this analysis was a big, multi-lab undertaking and it is likely that some points are not realistic to address, but we would like to hear your thoughts about them.

If there are requests that you feel are inappropriate or would require unreasonable experimental effort to address, please fully explain your arguments.

This information is intended for the editors but be sure to provide sufficient detail for us to adequately assess it. If we decide we would like to send any portion of the response to a reviewer we will first request permission and allow you to rephrase your response.

We would appreciate hearing from you as soon as possible so that we can take your comments into consideration when making a decision. If you expect this to take more than a few days please contact me. Please do not actually revise the paper at this time.

Thank you for your prompt attention to this matter.

Sincerely,
Arunima

Arunima Singh, Ph.D.
Associate Editor
Nature Methods

Reviewers' Comments:

Reviewer #1:
Remarks to the Author:
This manuscript describes the evaluation of glycoproteomics informatics solutions for glycopeptide identification. In the past two decades, the development of bioinformatics tools for glycopeptide identification is burgeoning due to the rapid development of mass spectrometry and data analysis tools. However, the diversity of software results in the difficulty to compare them in systematic manners. This manuscript is the outcome of teamwork among several experts in glyco field and software developers. The coordination and following data analysis were the main values of this manuscript. The manuscript was nicely written but the authors shall address following points in this manuscript.

1. In this study, the authors showed the high discordance among all participants and software. For N-glycopeptides, only 43 N-glycopeptides out of 2,556 unique N-glycopeptides were commonly reported by at least 75% of the teams. For O-glycopeptides, only 3 O-glycopeptides out of 1,192 unique O-glycopeptides were commonly reported by at least half of the teams. The high discordance showed the diversity of the software but also create confusion for the community. The authors shall discuss the main causes of this observation. Did it contribute from the searching algorithm, parameters, experience of the participants?
2. For O-glycopeptide analysis, it was unclear how the authors defined the identified O-glycopeptides. Did it consider both site information and glycan compositions on the same peptides?
3. The results of synthetic glycopeptide performance tests were very interesting. In Figure S5, six out of 22 teams could not identify this peptide. Half of them were from developers. Do the authors imply that these three tools are not reliable? Will authors suggest the society not to use them for glycopeptide mapping? What can readers learn from these data?
4. Last year, both MSFragger-glyco and O-pair showed their high performance, sensitivity and accuracy in glycopeptide mapping. Since the goal of this study is to provide a comprehensive comparison, the readers will be eager to know the comments for the latest development of glyco tools from the community. The authors shall include the results from these two tools.

Since the authors are the experts in the glyco-community, they shall provide the society clear comments/suggestions on how to use available tools for glycopeptide mapping and the direction for software development.

Reviewer #2:
Remarks to the Author:
N- and O-glycoproteomics of complex samples is a challenging affair, but has in recent years become

achievable due to developments in mass spectrometry and data analysis. In the presented manuscript, Kawahara R. and co-authors report a considerable effort to compare contemporary bioinformatics solutions for identifying glycopeptides from mass spectrometric data. Two raw data files, generated by LC-MS/MS from proteolytically digested human serum, were distributed across 22 participants. Each participant then applied their preferred data analysis method and reported back in a standardized format, after which the findings were scored and compared in a variety of ways.

I think the central premise of the manuscript, a comparison of contemporary database search strategies for analysis of complex glycoproteomics samples, is an exciting one and a much-needed step towards handling several of the pervasive challenges. I also think that the manuscript in its current form is too much focused on comparing participants rather than their search strategies and that the interpretability of the results is complicated by the lack of a "ground truth" sample in which the glycoprotein abundances and glycosylation heterogeneities are known. I invite the authors to have a look at the comments and suggestions below:

1. The dataset is ideally positioned to make good claims on search strategies, which, as the authors mention, can be quite diverse in the glycoproteomics field. As it is, the manuscript can be rather team-centric, with most comparisons made on a participant level (Fig 1, Fig 2, Fig 3, Fig S1, Fig S3, Fig S5). I would much rather see a comparison of the search engines and for those used multiple times (11x Byonic) how the search settings affect the results. For instance, how does the number of allowed peptide modifications influence the number of truncated glycan structures and what is the correlation between similar settings for Byonic and Protein Prospector? Ideally, a new user would be able to decide on an engine/approach with the help of this manuscript.

2. Along the same line, a user would ultimately like to know which engine/approach best reports on the actual glycosylation that is present in their sample. Serum glycoproteomics is challenging in this regard, because, while reviews help, this actual "ground truth" remains elusive. The authors themselves acknowledge this and say that the current study will even help with establishing the ground truth by taking the weighted findings across participants, but this makes it paradoxical to apply the "match with literature" as a scoring factor in the same study.
I would find it extremely valuable if the authors would not only perform the data analyses on an undefined serum sample, but also on a well-defined (spiked) mixture of glycoproteins (e.g., immunoglobulin G for simple N- glycans, fibrinogen for small sialylated N-glycans, erythropoeietin for large sialylated N-glycans and O-glycans) that have already been characterized by other methods (released glycan analysis, NMR, etc.). With such a sample the relative strengths and weaknesses of the analysis workflow can be established, which could then be used to infer the "ground truth" in the complex serum sample.

3. I am not sure whether using multiFuc and NeuGc as implicitly false positive is the right thing to do. Serum proteins are well-known to increase in fucosylation with inflammation (see next comment), while NeuGc might be incorporated on glycans via dietary intake. I think this point needs to be further substantiated, perhaps some fragment spectra can be shown which have been misassigned, or the compositions further proven by monosaccharide analysis/released glycan analysis. If "dummy glycans" are required for the search actions, perhaps this can be performed with nonsensical compositions (HexNAc1-10 or Hex1-10) or monosaccharides (dideoxyhexose or deoxy-N-acetylhexosamine).

4. Considering the changeability of glycosylation, it would be valuable to have more information on the commercial donor sample: age, gender, and perhaps inflammation status measured via IL-6 or CRP.

5. I think the inclusion of the synthetic glycopeptide is a good one and I appreciate the comparison thereof in Fig S5; the methods should still have a small section on how this peptide was generated and the structure determined. Could the sample have contained related (minor) glycopeptides that could also be monitored, for example, sialylation variants and miscleavages? Does the peptide also occur in serum samples without spiking?

6. Why did the authors perform only trypic digestion, rather than using different proteases or a combination of proteases? I can imagine that many search engines have been trained on tryptic peptides and that the results would be more divergent for peptides that do not C-terminate on lysine.

7. Why are developers and users treated differently, for example in Fig 3?

8. Both dependent fragmentation methods include a round of CID fragmentation, can the authors report whether this fragmentation was beneficial for the characterization of the glycoproteome, or whether the peptide fragments were generally not sufficient for spectral matching?

9. I would appreciate the inclusion of more raw data comparisons, for example: MS/MS of a glycopeptide that was annotated the same across platforms and of one that was differently annotated across platforms. Hopefully, this would make it insightful how ions are handled across algorithms.

10. Some teams search for Na+ and K+ (and Ca2+?) adducts, which are currently grouped. Can the authors comment on whether searching for these adducts is helpful/harmful for the analysis?

11. Currently, I find the title somewhat misleading. The comparison is mostly between labs (rather than search strategies) and the glycopeptide data is serum N-/O-glycoproteomics data specifically.

12. While multifucosylated species were excluded as false positive, some multifucosylated compositions are still visible in network graph Fig S4 (for example, H4N4F2S1, H3N3F2S2).

13. While I understand that not every search engine can be included the authors do mention several that still appear to be valuable points of comparison, including pGlyco, MSFragger-Glyco and/or O-Pair Search. I think their exclusion should either be expanded upon in the discussion, or, with the data being available, a comparison could still be incorporated.

Reviewer #3:
Remarks to the Author:
Title: Community Evaluation of Glycoproteomics Informatics Solutions Reveals High-Performance Search Strategies of Glycopeptide Data

Summary:
As part of the Human Proteome Project – Human Glycoproteomics Initiative, Kawahara et al. report the results of a community effort to document and evaluate several glycoproteomic search algorithms and software platforms. Two LC-MS/MS data files of glycopeptides enriched from human serum were provided to 22 teams of either software developers or expert users, and teams reported both N- and O-glycopeptides with a common template to enable thorough analyses of outputs and metrics. This

important study not only compares relative performance of analysis pipelines for glycopeptide identifications, but also identifies key settings, variables, and features of algorithms that impact glycoproteomic searches. The manuscript is information dense, but this is a good thing considering the number of metrics reported for glycopeptide data. The figures are well designed and organized to complement the data. The tables provided are particularly useful and will be among the most referenced part of the manuscript by readers, especially Table 3 that summarizes search setting and output features associated with the study. The Discussion is also insightful, both summarizing the results and putting this study in context with current work and future needs. The large spread in glycopeptide identifications with relatively little overlap is simultaneously surprising and concerning – which further underscores the need for studies like this and consensus data to be reported. This manuscript merits publication in Nature Methods after the following minor comments have been addressed.

1. An addition that would be helpful to underscore the conclusions of this manuscript would be a comparison of two different search strategies by the same team, rather than the inter-team comparisons done throughout. Using the lessons learned here, one could imagine a "high-coverage" search strategy and a "high-accuracy" search strategy that would vary in the parameters spelled out in Table 3. Having the same team perform two different searches using the same algorithm and the same data would show how the range of glycoproteomic identifications can vary even within the same user(s) if different metrics are used. This would provide a frame of reference for readers of how these decisions will affect their data. This could be done on a high quality glycoproteomics dataset that is already publish to demonstrate how the lessons gleaned from this work are applicable beyond the two data files studied here.

2. Performance tests N6 and O5 that evaluate NeuGc and multi-Fuc-glycopeptides are described to measure the average of non-NeuGc and non-Fuc≥2 containing glyco-PSMs. I understand this is done because a measure of 1 is best (i.e., no NeuGc or Fuc≥2 glyco-PSMs) and can be used in the overall average score calculations, but the naming of the tests is counterintuitive. Several times I found myself having to double or triple check my understanding that a high NeuGc/Multi-Fuc score actually meant FEW NeuGc/Multi-Fuc hits. I recommend the authors either rename these tests or more explicitly describe this inverse relationship in the text.

3. The y-axis label is obstructing numbers on the y-axis of Supplemental Figure S3i.

4. Supplementary S5c, where the score is calculated as an average of the sensitivity and specificity, the scores do not seem to match. For example, Team 1 has a sensitivity of 50 and a specificity of 100. How then is the average result in a score of 50? This trend holds for all scores in this figure. Could the authors clarify?

5. The authors do not discuss quantification much, other than to use spectral counting for some of the performance tests. In the Discussion, can the authors put the future of glycoproteomics quantitative strategies into context with the data they present here? If we have this much trouble identifying the same things, how can we imagine reliable quantification?

Author Revision Plan after Initial comments

Dear Nature Methods Editors, Dear Dr Singh,

We are very pleased to learn that the three assigned reviewers find merit in our study. As requested, we here provide a concise point-by-point response to each of their comments. Generally, we find the comments relevant, reasonable, and addressable; the feedback will help us to strengthen the manuscript further. We anticipate that we will be able to revise our manuscript accordingly within a relatively short period (few weeks). We would like to carry out a few additional data searches and data/literature mining and comparison efforts as suggested by the reviewers. The specific strategies we plan to take to address their comments are briefly described below. We have identified a few specific requests that we find are relevant but unfortunately are not feasible given the design of the study. Please do not hesitate to let us know should you require more specific details of how we plan to address the constructive feedback provided by the three reviewers.

Reviewers' Comments:

Reviewer #1:
Remarks to the Author:
This manuscript describes the evaluation of glycoproteomics informatics solutions for glycopeptide identification. In the past two decades, the development of bioinformatics tools for glycopeptide identification is burgeoning due to the rapid development of mass spectrometry and data analysis tools. However, the diversity of software results in the difficulty to compare them in systematic manners. This manuscript is the outcome of teamwork among several experts in glyco field and software developers. The coordination and following data analysis were the main values of this manuscript. The manuscript was nicely written but the authors shall address following points in this manuscript.

1. In this study, the authors showed the high discordance among all participants and software. For N-glycopeptides, only 43 N-glycopeptides out of 2,556 unique N- glycopeptides were commonly reported by at least 75% of the teams. For O- glycopeptides, only 3 O-glycopeptides out of 1,192 unique O-glycopeptides were commonly reported by at least half of the teams. The high discordance showed the diversity of the software but also create confusion for the community. The authors shall discuss the main causes of this observation. Did it contribute from the searching algorithm, parameters, experience of the participants?

Response: Our study is the first community effort to assess the relative performance of different glycoproteomics search engines and search strategies carried out by experts in the field. While we found

that several high-performance search engines and search strategies are now available to the community, we agree that our study showed a surprising high discordance between the glycopeptides reported by the participants. The observed discordance is indeed concerning, but (exactly for that reason) highly important to convey to the scientific community (as also pointed out by Reviewer 3) at a time where glycoproteomics studies are increasingly making their way into the scientific literature (surveyed in Chernykh et al., Biochem Soc Trans, 49(1):161, 2021). We have discussed the factors that contribute to the discrepancy between participants including the search engine, search settings and post-search filtering methods (p28). However, we agree that these factors could be highlighted further in the manuscript and we will in the revised version also comment on the relevance of the team experience for accurate glycopeptide analysis as suggested by the reviewer.

2. For O-glycopeptide analysis, it was unclear how the authors defined the identified O- glycopeptides. Did it consider both site information and glycan compositions on the same peptides?

Response: The reported N- and O-glycopeptides were assessed for correctness according to their peptide sequence + glycan composition as detailed in Table 2 (p14-15). Thus, we did not directly consider site information in this study. Site localisation is less important for sequon-located N-glycosylation where sites often can be accurately inferred from peptide identify information (see discussion p29). The need for a follow-up study that addresses the ability of software and users to accurately report on O-glycosylation sites and other important features such as glycopeptide quantitation was discussed (p30). However, we will add a sentence in the methods and results sections of the revised manuscript to further clarify how N- and O-glycopeptides were reported and assessed in this study.

3. The results of synthetic glycopeptide performance tests were very interesting. In Figure S5, six out of 22 teams could not identify this peptide. Half of them were from developers. Do the authors imply that these three tools are not reliable? Will authors suggest the society not to use them for glycopeptide mapping? What can readers learn from these data?

Response: Four out of nine developers did not identify the synthetic N-linked glycopeptide spiked into the serum sample. Only two expert user teams failed to report on any of the corresponding nine MS2 spectra shown in Supplementary Fig S5. Notably, the users were (unlike the developers) allowed to employ subjective post-search filtering of the output data likely explaining their better performance in this test. To ensure a fair and holistic assessment of the relative team performance, we decided that this synthetic N- glycopeptide challenge (N1) should contribute as only one of six equally important N-glycopeptide performance tests (Table 2, p14). Backed by statistics, Table 3 (p24) summarises what the data from this study have taught us. The search settings and the search output features associated with high performance glycoproteomics data analysis across various performance areas were delineated in this table to guide readers towards performing better glycoproteomics experiments. We agree that it

would be appropriate to elaborate on the data generated from the synthetic glycopeptide challenge since it represents an easy-to-understand performance test that unlike some of the other tests is based on a "ground truth". Note that we also plan to perform manual glycoprofiling of abundant serum glycoproteins identified in the shared datasets and compare these glycoprofiles to the literature and the glycopeptides reported by teams to enable more accurate protein-centric comparisons and thus more reliable conclusions to be drawn from this study (see below).

4. Last year, both MSFragger-glyco and O-pair showed their high performance, sensitivity and accuracy in glycopeptide mapping. Since the goal of this study is to provide a comprehensive comparison, the readers will be eager to know the comments for the latest development of glyco tools from the community. The authors shall include the results from these two tools.

Response: These are relevant suggestions that, however, are not feasible to implement due to the design and timing of the study and due to our promise to the participants (commercial and academics alike) at the outset of the study to ensure a fair and unbiased comparison between developers and expert users in the field. Thus, we strongly believe that including new developer teams at this late stage in the process would not be appropriate. On one hand, it would disadvantage the existing developer teams already included in the study many of whom have released improved software upgrades since the data analysis period (e.g. GPQuest v3.0), but were not allowed to include data from these improved versions after the close of the study as decided by the study committee. On the other hand, the preliminary study outcomes (e.g. the team reports including all the reported glycopeptides, the identified consensus glycopeptides and the spiked synthetic glycopeptide data) are now all available on public servers including BioRxiv, ProteomeXchange and GlyConnect, which would also unfairly give new participants an advantage. Thus, this study is essentially a snapshot of the performance of the software solutions at the time the data analysis was performed (->2019). See response to Reviewer 3 for more.

Since the authors are the experts in the glyco-community, they shall provide the society clear comments/suggestions on how to use available tools for glycopeptide mapping and the direction for software development.

Response: We provide in Table 3 a detailed breakdown of recommendations based on the lessons learned from this study. Only statistically supported recommendations were included in this table. Following the reviewers' suggestions (see below), we are planning to undertake an additional set of search engine-specific comparisons where we expect to identify several additional search settings relevant for the software most participants use (Byonic, n = 11) also impacting the glycoproteomics performance. We suggest including lessons learned from these additional analyses in Table 3. We will also include a paragraph that discusses the future direction of software development based on outcomes and findings from this study.

Reviewer #2:
Remarks to the Author:

N- and O-glycoproteomics of complex samples is a challenging affair, but has in recent years become achievable due to developments in mass spectrometry and data analysis. In the presented manuscript, Kawahara R. and co-authors report a considerable effort to compare contemporary bioinformatics solutions for identifying glycopeptides from mass spectrometric data. Two raw data files, generated by LC-MS/MS from proteolytically digested human serum, were distributed across 22 participants. Each participant then applied their preferred data analysis method and reported back in a standardized format, after which the findings were scored and compared in a variety of ways.

I think the central premise of the manuscript, a comparison of contemporary database search strategies for analysis of complex glycoproteomics samples, is an exciting one and a much-needed step towards handling several of the pervasive challenges. I also think that the manuscript in its current form is too much focused on comparing participants rather than their search strategies and that the interpretability of the results is complicated by the lack of a "ground truth" sample in which the glycoprotein abundances and glycosylation heterogeneities are known. I invite the authors to have a look at the comments and suggestions below:

1. The dataset is ideally positioned to make good claims on search strategies, which, as the authors mention, can be quite diverse in the glycoproteomics field. As it is, the manuscript can be rather team-centric, with most comparisons made on a participant level (Fig 1, Fig 2, Fig 3, Fig S1, Fig S3, Fig S5). I would much rather see a comparison of the search engines and for those used multiple times (11x Byonic) how the search settings affect the results. For instance, how does the number of allowed peptide modifications influence the number of truncated glycan structures and what is the correlation between similar settings for Byonic and Protein Prospector? Ideally, a new user would be able to decide on an engine/approach with the help of this manuscript.

Response: This is a relevant comment that we will address in the revised manuscript. We plan to perform search engine-centric comparisons of the use of the same software (e.g. Byonic, 11 teams) in attempts to identify search engine-specific search settings impacting the performance. Similar to the statistically significant associations already reported in Figure 3 and Table 3, we will aim to generate statistical support for such search engine- specific relationships, which, for example, could be integrated as additional panels in Figure 3 or form an additional figure/table.

2. Along the same line, a user would ultimately like to know which engine/approach best reports on the actual glycosylation that is present in their sample. Serum glycoproteomics is challenging in this regard, because, while reviews help, this actual "ground truth" remains elusive. The authors themselves acknowledge this and say that the current study will even help with establishing the

ground truth by taking the weighted findings across participants, but this makes it paradoxical to apply the "match with literature" as a scoring factor in the same study. I would find it extremely valuable if the authors would not only perform the data analyses on an undefined serum sample, but also on a well-defined (spiked) mixture of glycoproteins (e.g., immunoglobulin G for simple N- glycans, fibrinogen for small sialylated N-glycans, erythropoeietin for large sialylated N-glycans and O-glycans) that have already been characterized by other methods (released glycan analysis, NMR, etc.). With such a sample the relative strengths and weaknesses of the analysis workflow can be established, which could then be used to infer the "ground truth" in the complex serum sample.

Response: This is a relevant suggestion which we will attempt to address by performing manual glycoprofiling (site-specific N-glycan distribution) of a few select high-abundance serum N-glycoproteins in our sample to allow comparison to trusted literature of those some glycoproteins (e.g. IgG, ceruloplasmin, haptoglobin, alpha-1-antitrypsin, and alpha- 2-macroglobulin, Clerc et al. Glycoconj J 33, 309, 2016). This will allow us to make more confident comparisons to the literature (a feature used for several performance tests) and also enable protein- and site-centric comparison to glycopeptides reported by the individual teams. Collectively, these additional analyses will improve the accuracy and strengthen the conclusions of the study. Finally, we will also expand, as suggested by Reviewer 1, on our discussion of data obtained from the N1 synthetic glycopeptide challenge that unlike some of the other performance tests is actually based directly on a ground truth (see also response above and below for more).

3. I am not sure whether using multiFuc and NeuGc as implicitly false positive is the right thing to do. Serum proteins are well-known to increase in fucosylation with inflammation (see next comment), while NeuGc might be incorporated on glycans via dietary intake. I think this point needs to be further substantiated, perhaps some fragment spectra can be shown which have been misassigned, or the compositions further proven by monosaccharide analysis/released glycan analysis. If "dummy glycans" are required for the search actions, perhaps this can be performed with nonsensical compositions (HexNAc1-10 or Hex1-10) or monosaccharides (dideoxyhexose or deoxy-N-acetylhexosamine).

Response: While we appreciate the reviewer's comment, we are confident that the shared data files have been recorded from non-inflamed healthy serum (see below) and that the glycopeptides in the sample do not contain any significant NeuGc and only low levels of multi-Fuc glycofeatures as described on p15-16. However, we agree that it is relevant to show the evidence for our claims. Thus, we will show exemplar MS2 spectra of clearly misannotated multi-Fuc and NeuGc glycopeptides and show that NeuGc diagnostic fragment ions are absent in the raw data in the SI. A few O-glycopeptides and a single N-glycopeptides were identified as consensus glycopeptides (see Supplementary Fig S4), so these clearly exist in serum albeit at a low level as backed by literature and our own analyses. We will show annotated MS2 spectra for the multi-Fuc consensus glycopeptides to support their existence.

Collectively this will provide readers with the evidence confirming that glycopeptides reported to carry NeuGc can be considered misidentified. We recommend that glycopeptides reported with multi-Fuc should be carefully inspected by manual expert annotation to validate their identify, for example, by the presence of low mass B-type ions corresponding to the fragment ions for antennary fucosylation. We will discuss these points in the revised manuscript.

4. Considering the changeability of glycosylation, it would be valuable to have more information on the commercial donor sample: age, gender, and perhaps inflammation status measured via IL-6 or CRP.

Response: We have obtained information from the vendor regarding the commercial donor sample used for this study specifying that this product is “normal” and “non- immune” sera from “healthy” individuals. https://www.thermofisher.com/antibody/product/Normal-Human-Serum-Control/31876 and
https://www.thermofisher.com/order/genomedatabase/dataSheetPdf?producttype=antibody&productsubtype=antibody_control&produc tId=31876&version=141
Moreover, the excellent agreements of the glycan compositions and source proteins reported by the high-performance teams to the glycoproteins previously characterised from healthy normal serum (see e.g. Clerc et al. Glycoconj J 33, 309, 2016) support that the data investigated here were obtained from non-inflamed “healthy” serum. Therefore, we do not consider inflammation markers and acute phase proteins and their glycosylation features being present in relevant quantities to significantly impact the reported outcomes.

5. I think the inclusion of the synthetic glycopeptide is a good one and I appreciate the comparison thereof in Fig S5; the methods should still have a small section on how this peptide was generated and the structure determined. Could the sample have contained related (minor) glycopeptides that could also be monitored, for example, sialylation variants and miscleavages? Does the peptide also occur in serum samples without spiking?

Response: We will add experimental details of how the (homogenous) synthetic glycopeptide was generated and structurally verified in the Online Methods and/or the Extended Methods in the SI:

Glycopeptide synthesis
An Asn-building block carrying a doubly sialylated, biantennary N-glycan was purified from chicken egg yolk powder. Previous studies have confirmed that a disialylated, biantennary N-glycan carrying just [illegible]2-6 linked NeuAc residues is the major component of the chicken egg yolk hexapeptide1, 2. In short, this glycosylated hexapeptide was subjected to extensive proteolysis to generate a glycosylated Asn, which was then converted into a fluorenylmethoxycarbonyl (Fmoc) protected building block as described earlier2, 3. Using this glycosylated Asn building block, the glycopeptide was synthesised by solid phase

peptide synthesis3-5. The peptide sequence was inspired by a tryptic glycopeptide present in human protein C also found in human plasma (UniProtKB entry: P04070, 284EVFVHPNYSK293. The purity and structural integrity after deprotection and purification were confirmed to contain only the doubly sialyated, biantennary N-glycan using reversed phase LC-MS/MS as described earlier4.

1. Seko, A. et al. Occurence of a sialylglycopeptide and free sialylglycans in hen's egg yolk. Biochim Biophys Acta 1335, 23-32 (1997).
2. Alagesan, K. & Kolarich, D. Improved strategy for large scale isolation of sialylglycopeptide (SGP) from egg yolk powder. MethodsX 6, 773-778 (2019).
3. Yamamoto, N. et al. Solid-phase synthesis of sialylglycopeptides through selective esterification of the sialic acid residues of an Asn-linked complex-type sialyloligosaccharide. Angewandte Chemie 42, 2537-2540 (2003).
4. Alagesan, K., Hinneburg, H., Seeberger, P.H., Silva, D.V. & Kolarich, D. Glycan size and attachment site location affect electron transfer dissociation (ETD) fragmentation and automated glycopeptide identification. Glycoconj J 36, 487-493 (2019).
5. Stavenhagen, K. et al. Quantitative mapping of glycoprotein micro-heterogeneity and macro-heterogeneity: an evaluation of mass spectrometry signal strengths using synthetic peptides and glycopeptides. Journal of mass spectrometry : JMS 48, 627-639 (2013).

To this end, we unfortunately did not identify other minor glycoforms of the synthetic glycopeptide that could be used for additional performance testing as suggested by the reviewer.

6. Why did the authors perform only tryptic digestion, rather than using different proteases or a combination of proteases? I can imagine that many search engines have been trained on tryptic peptides and that the results would be more divergent for peptides that do not C-terminate on lysine.

Response: We designed the study to simulate typical experimental conditions commonly used across the glycoproteomics field. We will be happy to include a small paragraph in the discussion of the revised manuscript speculating on the software performance for non- tryptic glycopeptides as used by some laboratories.

7. Why are developers and users treated differently, for example in Fig 3?

Response: The relative team performance was compared within (not between) the developer and expert user groups since these were given slightly different instructions to complete the study as described in the Online Methods (p31): “The expert user teams were free to use any search engine(s) at their disposal including manual annotation and filtering of search output. Developers were asked to return the list of glycopeptide identifications directly from their own software without manual post-search filtering.”. We will make sure this is more clearly communicated in the revised manuscript.

8. Both dependent fragmentation methods include a round of CID fragmentation, can the authors report whether this fragmentation was beneficial for the characterization of the glycoproteome, or whether the peptide fragments were generally not sufficient for spectral matching?

Response: The participants generally reported very few glycopeptides based on CID- MS/MS spectral evidence (p9). The number of reported glycopeptides based on CID spectra were significantly lower than HCD spectra (File B: HCD = 2,736, CID = 151). This under- reporting of CID-MS/MS spectra can most likely be attributed to the fact that relatively few software tools can handle this type of fragmentation data rather than a poor spectral quality of the CID spectra. Notably, CID-MS/MS is known to inform predominantly on the glycan part of the glycopeptides as opposed to the more peptide-centric HCD and site- centric EThcD data, providing another reason why only few teams report glycopeptides based on this fragmentation method. Of the nine developers, only two software solutions used CID spectral evidence to complete their reports (Team 1 – IQ-GPA, only high- resolution CID and Team 8 GlycoPAT, both high- and low-resolution CID data).
Surprisingly, the developer of Byonic (Team 4) did not use the available CID spectra, while most of the Byonic users in fact did. These relevant observations of CID-MS/MS will be discussed in the revised manuscript.

9. I would appreciate the inclusion of more raw data comparisons, for example: MS/MS of a glycopeptide that was annotated the same across platforms and of one that was differently annotated across platforms. Hopefully, this would make it insightful how ions are handled across algorithms.

Response: This is a good suggestion. We will in the revised manuscript include MS/MS spectra that were correctly identified across most platforms (consensus glycopeptides) and provide examples of the MS/MS spectra that were differently annotated across software solutions. This would provide insight into the features associated with the “easy-to-assign” spectra across search engines and indicate spectral features (e.g. low S/N, ion types, charge states) that make fragment spectra challenging to correctly annotate.

10. Some teams search for Na+ and K+ (and Ca2+?) adducts, which are currently grouped. Can the authors comment on whether searching for these adducts is helpful/harmful for the analysis?

Response: The only team that included adducts in their search space was Team 12. Team 12 reported adducted N-glycopeptides based on EThcD spectral evidence (File B). It is therefore difficult to make any broad conclusions as to whether searching for adducts is beneficial for the analysis. However, in attempts to address the point raised by the reviewer we plan to run a series of in-house Byonic-based searches of File B in which we, for example, will test the output of searches performed with and without

Na+ and K+ adduct in the search space while keeping the many other search settings constant. The outcomes of these efforts will be described in the revised manuscript.

11. Currently, I find the title somewhat misleading. The comparison is mostly between labs (rather than search strategies) and the glycopeptide data is serum N-/O- glycoproteomics data specifically.

Response: We will rephrase the title to include “serum N-/O-glycoproteomics data”. However, given the plan to include a greater search engine-centric focus and thereby exploring more features of the search strategies employed by teams using the same software (see above), we will keep this part in the title in the revised manuscript.

12. While multifucosylated species were excluded as false positive, some multifucosylated compositions are still visible in network graph Fig S4 (for example, H4N4F2S1, H3N3F2S2).

Response: We appreciate the comment by the reviewer and have therefore rephrased how we tackle the absent NeuGc- and low abundance multiFuc glycofeatures in the study (see response above). We agree that we have two consensus O-glycopeptides and a single consensus N-glycopeptides carrying double fuc as correctly shown in the network graphs in Fig S4. We will provide MS/MS spectral evidence for these. We will also provide more evidence of the absence of NeuGc in the shared data files an include misannotated multi- fucosylated glycopeptides reported across the teams (see response above for more).

13. While I understand that not every search engine can be included the authors do mention several that still appear to be valuable points of comparison, including pGlyco, MSFragger-Glyco and/or O-Pair Search. I think their exclusion should either be expanded upon in the discussion, or, with the data being available, a comparison could still be incorporated.

Response: See response above to Reviewer 1, Question 4.
Further, during the early phases of the study period, we reached out to all major developers that did not actively sign up to the study including pGlyco2.0 (as mentioned by Reviewer 2). This developer team accepted to be part of the study, but unfortunately did not provide data generated with pGlyco2.0. We therefore argue that we have been inclusive in our approach and that all major search engines at the time the study was conducted were given the opportunity to participate. As described (p29-30) follow-up studies comparing the performance of the very latest glycoproteomics software upgrades and informatics solutions not included in this study are therefore warranted and may form part of future Human Glycoproteomics Initiative studies. MSFragger-glyco, O-pair and pGlyco were all named and the original papers cited in the manuscript (p29).

Reviewer #3:
Remarks to the Author:
Title: Community Evaluation of Glycoproteomics Informatics Solutions Reveals High- Performance Search Strategies of Glycopeptide Data

Summary:
As part of the Human Proteome Project – Human Glycoproteomics Initiative, Kawahara et al. report the results of a community effort to document and evaluate several glycoproteomic search algorithms and software platforms. Two LC-MS/MS data files of glycopeptides enriched from human serum were provided to 22 teams of either software developers or expert users, and teams reported both N- and O-glycopeptides with a common template to enable thorough analyses of outputs and metrics. This important study not only compares relative performance of analysis pipelines for glycopeptide identifications, but also identifies key settings, variables, and features of algorithms that impact glycoproteomic searches. The manuscript is information dense, but this is a good thing considering the number of metrics reported for glycopeptide data. The figures are well designed and organized to complement the data. The tables provided are particularly useful and will be among the most referenced part of the manuscript by readers, especially Table 3 that summarizes search setting and output features associated with the study. The Discussion is also insightful, both summarizing the results and putting this study in context with current work and future needs. The large spread in glycopeptide identifications with relatively little overlap is simultaneously surprising and concerning – which further underscores the need for studies like this and consensus data to be reported. This manuscript merits publication in Nature Methods after the following minor comments have been addressed.

1. An addition that would be helpful to underscore the conclusions of this manuscript would be a comparison of two different search strategies by the same team, rather than the inter-team comparisons done throughout. Using the lessons learned here, one could imagine a "high-coverage" search strategy and a "high-accuracy" search strategy that would vary in the parameters spelled out in Table 3. Having the same team perform two different searches using the same algorithm and the same data would show how the range of glycoproteomic identifications can vary even within the same user(s) if different metrics are used. This would provide a frame of reference for readers of how these decisions will affect their data. This could be done on a high quality glycoproteomics dataset that is already published to demonstrate how the lessons gleaned from this work are applicable beyond the two data files studied here.

Response: This is an excellent idea. We will on one hand expand on comparisons made between teams that used Byonic in attempts to identify search engine-specific search settings impacting the performance (see also response above) and on the other hand also perform a few systematic in-house searches on the shared data files using lessons learned from this study. The aim is to demonstrate that

these controlled searches will “translate” to actual benefits for the glycopeptide data analysis by changing only a few key variables at a time. The suggestions of replicating a high-coverage” search strategy and a “high- accuracy” search strategy is a great suggestion. We will discuss the findings from these additional activities in the revised manuscript.

2. Performance tests N6 and O5 that evaluate NeuGc and multi-Fuc-glycopeptides are described to measure the average of non-NeuGc and non-Fuc≥2 containing glyco-PSMs. I understand this is done because a measure of 1 is best (i.e., no NeuGc or Fuc≥2 glyco- PSMs) and can be used in the overall average score calculations, but the naming of the tests is counterintuitive. Several times I found myself having to double or triple check my understanding that a high NeuGc/Multi-Fuc score actually meant FEW NeuGc/Multi-Fuc hits. I recommend the authors either rename these tests or more explicitly describe this inverse relationship in the text.

Response: We will clarify and rephrase this performance test in the revised manuscript.

3. The y-axis label is obstructing numbers on the y-axis of Supplemental Figure S3i.

Response: We will fix this mistake. Thank you for pointing this out.

4. Supplementary S5c, where the score is calculated as an average of the sensitivity and specificity, the scores do not seem to match. For example, Team 1 has a sensitivity of 50 and a specificity of 100. How then is the average result in a score of 50? This trend holds for all scores in this figure. Could the authors clarify?

Response: The score was in fact calculated by multiplying (not averaging) the sensitivity score and specificity score from this performance test. We apologise for this mistake. We will correct the description in the revised manuscript.

5. The authors do not discuss quantification much, other than to use spectral counting for some of the performance tests. In the Discussion, can the authors put the future of glycoproteomics quantitative strategies into context with the data they present here? If we have this much trouble identifying the same things, how can we imagine reliable quantification?

Response: We agree and will expand on the need to assess glycopeptide quantitation in future comparison studies (p30) in the revised manuscript.

Decision Letter, first revision:

14th May 2021

Dear Dr. Thaysen-Andersen,

Thank you for your letter detailing how you would respond to the reviewer concerns regarding your Analysis, "Community Evaluation of Glycoproteomics Informatics Solutions Reveals High-Performance Search Strategies of Glycopeptide Data". We have decided to invite you to revise your manuscript as you have outlined, before we reach a final decision on publication. While we understand that some of the analysis cannot be performed since the datasets are now public, we would like to request that you discuss more clearly the fact that there are newer tools available that aren't compared in this analysis and that this is a limitation of the study.

Please do not hesitate to contact me if you have any questions or would like to discuss these revisions further.

When revising your paper:

* include a point-by-point response to the reviewers and to any editorial suggestions

* please underline/highlight any additions to the text or areas with other significant changes to facilitate review of the revised manuscript

* address the points listed described below to conform to our open science requirements

* ensure it complies with our general format requirements as set out in our guide to authors at www.nature.com/naturemethods

* resubmit all the necessary files electronically by using the link below to access your home page

[REDACTED]

Note: This URL links to your confidential home page and associated information about manuscripts you may have submitted, or that you are reviewing for us. If you wish to forward this email to co-authors, please delete the link to your homepage.

We hope to receive your revised paper within 6 weeks. If you cannot send it within this time, please let us know. In this event, we will still be happy to reconsider your paper at a later date so long as nothing similar has been accepted for publication at Nature Methods or published elsewhere.

OPEN SCIENCE REQUIREMENTS

REPORTING SUMMARY AND EDITORIAL POLICY CHECKLISTS
When revising your manuscript, please update your reporting summary and editorial policy checklists.

Reporting summary: https://www.nature.com/documents/nr-reporting-summary.zip
Editorial policy checklist: https://www.nature.com/documents/nr-editorial-policy-checklist.zip

If your paper includes custom software, we also ask you to complete a supplemental reporting summary.

Software supplement: https://www.nature.com/documents/nr-software-policy.pdf

Please submit these with your revised manuscript. They will be available to reviewers to aid in their evaluation if the paper is re-reviewed. If you have any questions about the checklist, please see http://www.nature.com/authors/policies/availability.html or contact me.

Please note that these forms are dynamic 'smart pdfs' and must therefore be downloaded and completed in Adobe Reader. We will then flatten them for ease of use by the reviewers. If you would like to reference the guidance text as you complete the template, please access these flattened versions at http://www.nature.com/authors/policies/availability.html.

IMAGE INTEGRITY
When submitting the revised version of your manuscript, please pay close attention to our href="https://www.nature.com/nature-research/editorial-policies/image-integrity">Digital Image Integrity Guidelines. and to the following points below:

-- that unprocessed scans are clearly labelled and match the gels and western blots presented in figures.
-- that control panels for gels and western blots are appropriately described as loading on sample processing controls
-- all images in the paper are checked for duplication of panels and for splicing of gel lanes.

Finally, please ensure that you retain unprocessed data and metadata files after publication, ideally archiving data in perpetuity, as these may be requested during the peer review and production process or after publication if any issues arise.

DATA AVAILABILITY
Please include a "Data availability" subsection in the Online Methods. This section should inform readers about the availability of the data used to support the conclusions of your study, including accession codes to public repositories, references to source data that may be published alongside the paper, unique identifiers such as URLs to data repository entries, or data set DOIs, and any other statement about data availability. At a minimum, you should include the following statement: "The data that support the findings of this study are available from the corresponding author upon request", describing which data is available upon request and mentioning any restrictions on availability. If DOIs are provided, please include these in the Reference list (authors, title, publisher (repository name), identifier, year). For more guidance on how to write this section please see:
http://www.nature.com/authors/policies/data/data-availability-statements-data-citations.pdf

CODE AVAILABILITY
Please include a “Code Availability” subsection in the Online Methods which details how your custom code is made available. Only in rare cases (where code is not central to the main conclusions of the paper) is the statement “available upon request” allowed (and reasons should be specified).

We request that you deposit code in a DOI-minting repository such as Zenodo, Gigantum or Code Ocean and cite the DOI in the Reference list. We also request that you use code versioning and provide a license.

For more information on our code sharing policy and requirements, please see: https://www.nature.com/nature-research/editorial-policies/reporting-standards#availability-of-computer-code

MATERIALS AVAILABILITY
As a condition of publication in Nature Methods, authors are required to make unique materials promptly available to others without undue qualifications.

Authors reporting new chemical compounds must provide chemical structure, synthesis and characterization details. Authors reporting mutant strains and cell lines are strongly encouraged to use established public repositories.

More details about our materials availability policy can be found at https://www.nature.com/nature-portfolio/editorial-policies/reporting-standards#availability-of-materials

ORCID
Nature Methods is committed to improving transparency in authorship. As part of our efforts in this direction, we are now requesting that all authors identified as ‘corresponding author’ on published papers create and link their Open Researcher and Contributor Identifier (ORCID) with their account on the Manuscript Tracking System (MTS), prior to acceptance. This applies to primary research papers only. ORCID helps the scientific community achieve unambiguous attribution of all scholarly contributions. You can create and link your ORCID from the home page of the MTS by clicking on ‘Modify my Springer Nature account’. For more information please visit please visit www.springernature.com/orcid.

Please do not hesitate to contact me if you have any questions or would like to discuss these revisions further. We look forward to seeing the revised manuscript and thank you for the opportunity to consider your work.

Sincerely,
Arunima

Arunima Singh, Ph.D.
Associate Editor

Nature Methods

[DUPLICATE REVIEWER COMMENTS REMOVED]

Author Rebuttal, first revision:

Dear Nature Methods Editors, Dear Dr Singh,

We are very pleased to learn that the three assigned reviewers find merit in our study. Generally, we find the comments highly relevant, reasonable, and addressable; the constructive feedback has helped us strengthen the manuscript further and we thank the three expert reviewers for their valuable comments. We have in the revision process carried out several additional data searches and data/literature mining and comparative analyses as suggested by the reviewers. Please note that we have addressed a point raised by the editor at the end of this letter. An overview delineating all major changes introduced in the revised manuscript including on overview of the new and updated graphical elements has also been provided at the end of this document for your convenience. A few specific points raised by the reviewers were indeed relevant but unfortunately were not feasible given the design of the study. As requested, we here provide a concise point-by-point response to each of their comments.

Reviewers' Comments:

Reviewer #1:

Remarks to the Author:
This manuscript describes the evaluation of glycoproteomics informatics solutions for glycopeptide identification. In the past two decades, the development of bioinformatics tools for glycopeptide identification is burgeoning due to the rapid development of mass spectrometry and data analysis tools. However, the diversity of software results in the difficulty to compare them in systematic manners. This manuscript is the outcome of teamwork among several experts in glyco field and software developers. The coordination and following data analysis were the main values of this manuscript. The manuscript was nicely written but the authors shall address following points in this manuscript.

Response: We are pleased that the reviewer finds value in our manuscript. We thank the reviewer for their excellent suggestions that we have addressed below.

1. In this study, the authors showed the high discordance among all participants and software. For N-glycopeptides, only 43 N-glycopeptides out of 2,556 unique N- glycopeptides were commonly reported by at least 75% of the teams. For O- glycopeptides, only 3 O-glycopeptides out of 1,192 unique O-glycopeptides were commonly reported by at least half of the teams. The high discordance showed the diversity of the software but also create confusion for the community. The authors shall discuss the main causes of this observation. Did it contribute from the searching algorithm, parameters, experience of the participants?

Response: Our study is the first community effort to assess the relative performance of different glycoproteomics search engines and search strategies carried out by experts in the field. While we found that several high-performance search engines and search strategies are now available to the community, we agree that our study showed a surprisingly high discordance between the glycopeptides reported by the participants, even the ones using the same search engine. The observed discordance is indeed concerning, but (exactly for that reason) highly important to convey to the scientific community (as also pointed out by Reviewer 3) at a time where glycoproteomics studies are increasingly making their way into the scientific literature (surveyed in Chernykh et al., Biochem Soc Trans, 49(1):161, 2021). To the reviewer's specific question, while our analyses indicated that the data input and in particular, the choice of search engine, search settings and search output filtering impact the search performance, we did not find that the (self-reported) team experience was associated with the performance in our study. We have now expanded the discussion of these factors that do contribute to the discrepancy between participants and impact performance (Discussion, pp31-32).

2. For O-glycopeptide analysis, it was unclear how the authors defined the identified O- glycopeptides. Did it consider both site information and glycan compositions on the same peptides?

Response: The reported N- and O-glycopeptides were assessed for correctness according to their peptide sequence + glycan composition as detailed in Table 2. Thus, we did not directly consider site information in this study. Site localisation is less important for sequon-located N-glycosylation, where sites often can be accurately inferred from peptide identify information (see Discussion p32-33). The need for a follow-up study that addresses the ability of software and users to accurately report on O-glycosylation sites and other important features such as glycopeptide quantitation was mentioned in the Discussion (p33). We have added a sentence in the Results section (p15) of the revised manuscript to further clarify that N- and O-glycopeptides were reported and assessed to the level of the peptide sequence and glycan composition in this study.

3. The results of synthetic glycopeptide performance tests were very interesting. In Figure S5, six out of 22 teams could not identify this peptide. Half of them were from developers. Do the authors imply that these three tools are not reliable? Will authors suggest the society not to use them for glycopeptide mapping? What can readers learn from these data?

Response: Four out of nine developers did not identify the synthetic N-linked glycopeptide spiked into the serum sample. Only two expert user teams failed to report on any of the corresponding 12 MS2 spectra from File A and B, as shown in the updated Supplementary Figure S8 (former S5). Notably, the users were (unlike the developers) allowed to employ subjective post-search filtering of the output data, likely explaining their better performance in this test. To ensure a fair and holistic assessment of the relative team performance, we decided that this synthetic N-glycopeptide challenge (N1) should contribute as only one of six equally important N-glycopeptide performance tests (Table 2). Backed by statistics, Table 3 summarises what the data from the team-wide analysis of this study have taught us. The search settings and the search output features associated with high performance glycoproteomics data analysis across various performance areas were delineated in this table to guide readers towards performing better glycoproteomics experiments. We agree with the reviewer that it is appropriate to elaborate on the data generated from the synthetic glycopeptide challenge since it represents an easy-to-understand performance test that unlike some of the other tests (N2-N3, O1-O2) is based on a "ground truth" and we have done this in the Results (p19). Note that we also have now performed manual glycoprofiling of four abundant serum glycoproteins in File B as another measure of performance and compared these glycoprofiles to the literature and the glycopeptides reported by teams which have enabled greater confidence in the scoring and ranking process of teams and have provided readers with a more direct recommendation of software parameters to be aware of for accurate data interpretation (see below).

4. Last year, both MSFragger-glyco and O-pair showed their high performance, sensitivity and accuracy in glycopeptide mapping. Since the goal of this study is to provide a comprehensive comparison, the readers will be eager to know the comments for the latest development of glyco tools from the community. The authors shall include the results from these two tools.

Response: These are relevant suggestions. The glycoproteomics field is indeed moving at an exciting and exponential pace. The explosion of data has translated to the continued development and updating of tools, many of which featured in this extensive international consortium. Although we would like to include these very recent additional tools suggested by the reviewer, we simply cannot retrospectively fit their results into the study due to the design and timing of the study and due to our promise to the participants (commercial and academics alike) at the outset of the study to ensure a fair and unbiased comparison between developers and expert users in the field. Thus, we strongly believe that including new developer teams at this late stage in the process would not be appropriate. On the one hand, it would disadvantage the existing developer teams already included in the study, many of whom have released improved software upgrades since the data analysis period (e.g. GPQuest v3.0), but were not allowed to include data from these improved versions after the close of the study as decided by the study committee. On the other hand, the preliminary study outcomes (e.g. the team reports including all the reported glycopeptides, the identified consensus glycopeptides and the spiked synthetic

glycopeptide data) are now all available on public servers, including BioRxiv, ProteomeXchange and GlyConnect, which would also unfairly give new participants an advantage. Thus, this study is essentially a snapshot of the performance of the software solutions at the time the data analysis was performed. We have discussed these aspects including the development of exciting new software for glycopeptide analysis, including MSFragger-glyco and O-pair in the revised manuscript (Discussion, p33). See also response to Reviewer 3 for more.

Since the authors are the experts in the glyco-community, they shall provide the society clear comments/suggestions on how to use available tools for glycopeptide mapping and the direction for software development.

Response: We provide in Table 3 a detailed breakdown of recommendations based on the lessons learned from the team-wide (software-independent) analysis performed in this study. Only statistically supported recommendations were included in this table. Following the reviewers' suggestions (see below), we have performed a comprehensive search engine- centric analysis where we identified several performance-associated search settings relevant for the software used by most participants (Byonic, n = 11). We have included these new data in a new Figure 4 that also includes data from a series of systematic searches we performed using Byonic, as well as details of the optimised search strategies we recommend the community to use based on our findings. Data and findings from Figure 4 are presented in Results (pp22-24). We have also included a paragraph that discusses the future direction of software development and recommendations based on outcomes and findings from this study (Discussion, pp30-31).

Reviewer #2:
Remarks to the Author:

N- and O-glycoproteomics of complex samples is a challenging affair, but has in recent years become achievable due to developments in mass spectrometry and data analysis. In the presented manuscript, Kawahara R. and co-authors report a considerable effort to compare contemporary bioinformatics solutions for identifying glycopeptides from mass spectrometric data. Two raw data files, generated by LC-MS/MS from proteolytically digested human serum, were distributed across 22 participants. Each participant then applied their preferred data analysis method and reported back in a standardized format, after which the findings were scored and compared in a variety of ways.

I think the central premise of the manuscript, a comparison of contemporary database search strategies for analysis of complex glycoproteomics samples, is an exciting one and a much-needed step towards handling several of the pervasive challenges. I also think that the manuscript in its current form is too much focused on comparing participants rather than their search strategies and that the interpretability of the results is complicated by the lack of a "ground truth" sample in which

the glycoprotein abundances and glycosylation heterogeneities are known. I invite the authors to have a look at the comments and suggestions below:

Response: We are pleased that the reviewer finds our manuscript relevant. We sincerely thank the reviewer for their highly constructive suggestions that we have addressed below.

1. The dataset is ideally positioned to make good claims on search strategies, which, as the authors mention, can be quite diverse in the glycoproteomics field. As it is, the manuscript can be rather team-centric, with most comparisons made on a participant level (Fig 1, Fig 2, Fig 3, Fig S1, Fig S3, Fig S5). I would much rather see a comparison of the search engines and for those used multiple times (11x Byonic) how the search settings affect the results. For instance, how does the number of allowed peptide modifications influence the number of truncated glycan structures and what is the correlation between similar settings for Byonic and Protein Prospector? Ideally, a new user would be able to decide on an engine/approach with the help of this manuscript.

Response: We would like to thank the reviewer for this relevant comment that we have addressed in the revised manuscript. We have now performed a comprehensive search engine-centric analysis of data from the teams that use the same software (e.g. Byonic, 11 teams), which has led to the inclusion of a new Figure 4. The Byonic-centric analysis showed that teams used this search engine very differently, as illustrated by highly discrepant search settings across teams, and suggested that several of these search settings impact the performance. We, therefore, decided to also perform a series of systematic in-house searches using Byonic to identify how individual search settings (and data input, i.e. fragmentation type) impact the search performance under controlled conditions. These efforts not only led to the identification of multiple performance-associated search variables but also enabled us to improve and recommend several search strategies for "high accuracy" and "high coverage" glycoproteomics, findings that also have been included in the new Figure 4 and discussed in the text (Results, pp22-24). We anticipate that readers will find these additional analyses and recommendations useful when deciding on search engines and search strategies for their own glycoproteomics experiments (Discussion, pp29-32).

2. Along the same line, a user would ultimately like to know which engine/approach best reports on the actual glycosylation that is present in their sample. Serum glycoproteomics is challenging in this regard, because, while reviews help, this actual "ground truth" remains elusive. The authors themselves acknowledge this and say that the current study will even help with establishing the ground truth by taking the weighted findings across participants, but this makes it paradoxical to apply the "match with literature" as a scoring factor in the same study. I would find it extremely valuable if the authors would not only perform the data analyses on an undefined serum sample, but also on a well-defined (spiked) mixture of glycoproteins (e.g., immunoglobulin G for simple N- glycans, fibrinogen for small sialylated N-glycans, erythropoeietin for large sialylated N-glycans and O-glycans)

that have already been characterized by other methods (released glycan analysis, NMR, etc.). With such a sample the relative strengths and weaknesses of the analysis workflow can be established, which could then be used to infer the “ground truth” in the complex serum sample.

Response: These are relevant comments. The reviewer is correct that a shortcoming of the original manuscript was that several performance tests (i.e. N2-N3) were based on the match to robust literature of normal human serum. To further generate 'ground-truth' in addition to the spiked glycopeptide standard, we have now performed manual glycoprofiling (site- specific N-glycan distribution) of four high-abundance serum N-glycoproteins in our sample (IgG1, ceruloplasmin, haptoglobin, alpha-1-antitrypsin) and found an excellent quantitative agreement to relevant literature (R2 = 0.85-0.99, see new Supplementary Figure S4 for data). These findings strongly suggest that the glycosylation found in the serum sample used in this study accurately matches the glycosylation of normal serum studied by others, and validate the use of literature for the performance tests (Results, p10). Additionally, we have used the data from this manual glycoprofiling analysis to assess the relative performance of the teams in attempts to validate the scoring and ranking of teams using a “ground truth” rather than relying in part on inference from literature. Importantly, we found an excellent match between the scoring based on the original scoring method (N1-N6) and the new site- specific glycopeptide profiling data, which validates our scoring and ranking approach (see new Supplementary Figure S10 for data). Collectively, these additional analyses, which have been discussed in the Results section (p20) in the revised manuscript, have therefore improved the accuracy and strengthen the conclusions of the study.

3. I am not sure whether using multiFuc and NeuGc as implicitly false positive is the right thing to do. Serum proteins are well-known to increase in fucosylation with inflammation (see next comment), while NeuGc might be incorporated on glycans via dietary intake. I think this point needs to be further substantiated, perhaps some fragment spectra can be shown which have been misassigned, or the compositions further proven by monosaccharide analysis/released glycan analysis. If “dummy glycans” are required for the search actions, perhaps this can be performed with nonsensical compositions (HexNAc1-10 or Hex1-10) or monosaccharides (dideoxyhexose or deoxy-N-acetylhexosamine).

Response: While we highly appreciate the reviewer’s relevant comment, we are confident that the shared data files have been generated from non-inflamed healthy serum (see below) and that the glycopeptides in the sample do not contain any significant NeuGc and only low levels of detectable multi-Fuc glycan features. However, we agree that it is relevant to show the evidence for our claims. Thus, we have now substantiated these points by the inclusion of additional data shown in a new Supplementary Figure S5. This new figure confirms via a completely empty XIC MS/MS trace of NeuGc diagnostic ions (m/z 308/290 ion pair) and exemplar misannotated MS/MS spectra of falsely reported NeuGc-containing glycopeptides that NeuGc is not a feature of the studied sample. Further, the figure

shows via sparse XIC MS/MS signals for diagnostic ions of antenna Fuc (m/z 512/803, and thus by extension, glycopeptides carrying multi-Fuc containing BOTH core AND antenna-Fuc) and multiple examples of correctly and incorrectly annotated MS/MS spectra that multi-Fuc glycopeptides do exist but are very rare in our data. Collectively, this data validates our approach to use NeuGc and multi-Fuc as implicitly false positive for one of the six complementary performance tasks. We have discussed this new supporting data in the Results section (pp10- 11 and 15-16) in the revised manuscript.

4. Considering the changeability of glycosylation, it would be valuable to have more information on the commercial donor sample: age, gender, and perhaps inflammation status measured via IL-6 or CRP.

Response: We have obtained information from the vendor regarding the commercial donor serum sample used for this study: the product is “normal” and “non-immune” sera from “healthy” individuals. https://www.thermofisher.com/antibody/product/Normal-Human- Serum-Control/31876
and
https://www.thermofisher.com/order/genomedatabase/dataSheetPdf?producttype=antibody&product subtype=antibody_control&produc tId=31876&version=141. Moreover, the excellent agreement between the quantitative site- specific N-glycan profiling data of four high-abundance serum glycoproteins (selected because they are considered acute-phase proteins, i.e. elevated and aberrantly glycosylated during inflammation) to the site-specific glycosylation of these proteins previously characterised from healthy normal serum support that the data investigated in our study were obtained from non-inflamed “healthy” serum (see also response to point 2 above and the new Supplementary Figure S4 for data). Therefore, we do not consider inflammation markers and aberrant glycosylation features of acute-phase proteins being present in relevant quantities to significantly impact the reported outcomes. We have discussed the new Supplementary Figure S4 and that it serves to validate the use of literature for some of the performance tests in the revised manuscript (Results, p10).

5. I think the inclusion of the synthetic glycopeptide is a good one and I appreciate the comparison thereof in Fig S5; the methods should still have a small section on how this peptide was generated and the structure determined. Could the sample have contained related (minor) glycopeptides that could also be monitored, for example, sialylation variants and miscleavages? Does the peptide also occur in serum samples without spiking?

Response: We thank the reviewer for pointing out this mistake. We have now added the experimental details of how the (homogenous) synthetic glycopeptide was generated and structurally verified in the Extended Methods in the SI noting that the manuscript has a word limit precluding inclusion of this additional text in the main text.

The following references were also added to the Extended Methods section in the SI:

1. Seko, A. et al. Occurence of a sialylglycopeptide and free sialylglycans in hen's egg yolk. Biochim Biophys Acta 1335, 23-32 (1997).
2. Alagesan, K. & Kolarich, D. Improved strategy for large scale isolation of sialylglycopeptide (SGP) from egg yolk powder. MethodsX 6, 773-778 (2019).
3. Yamamoto, N. et al. Solid-phase synthesis of sialylglycopeptides through selective esterification of the sialic acid residues of an Asn-linked complex-type sialyloligosaccharide. Angewandte Chemie 42, 2537-2540 (2003).
4. Alagesan, K., Hinneburg, H., Seeberger, P.H., Silva, D.V. & Kolarich, D. Glycan size and attachment site location affect electron transfer dissociation (ETD) fragmentation and automated glycopeptide identification. Glycoconj J 36, 487-493 (2019).
5. Stavenhagen, K. et al. Quantitative mapping of glycoprotein micro-heterogeneity and macro-heterogeneity: an evaluation of mass spectrometry signal strengths using synthetic peptides and glycopeptides. Journal of mass spectrometry : JMS 48, 627-639 (2013).

To the other constructive comment raised by the reviewer, we, unfortunately, did not identify other minor glycoforms of the synthetic glycopeptide that could be used for additional performance testing, but we would like to inform the reviewer that we have updated the Supplementary Figure S8 to now display all 12 MS/MS spectra from File A-B that were originally used for the performance testing (N1). No performance scores were changed as a result of this update. We have also improved the annotation by labelling each spectrum with colour coded asterisks which we hope readers will find useful.

6. Why did the authors perform only tryptic digestion, rather than using different proteases or a combination of proteases? I can imagine that many search engines have been trained on tryptic peptides and that the results would be more divergent for peptides that do not C-terminate on lysine.

Response: We designed the study to simulate typical experimental conditions commonly used across the glycoproteomics field as described in Discussion section (p32). This is the reason we used trypsin as the protease in this study. We have included a small paragraph in the Discussion (p33) of the revised manuscript highlighting that the software performance for non-tryptic glycopeptides (as well as other interesting parameters such as TMT tagged glycopeptides and step-HCD) should be tested in future comparative studies.

7. Why are developers and users treated differently, for example in Fig 3?

Response: The relative team performance was compared within (not between) the developer and expert user groups since these were given slightly different instructions to complete the study as described in the Online Methods (p35): "The expert user teams were free to use any search engine(s) at their disposal including manual annotation and filtering of search output. Developers were asked to

return the list of glycopeptide identifications directly from their own software without manual post-search filtering.”. We have emphasised this important detail in the Results (p11) and Online Methods (p35) of the revised manuscript.

8. Both dependent fragmentation methods include a round of CID fragmentation, can the authors report whether this fragmentation was beneficial for the characterization of the glycoproteome, or whether the peptide fragments were generally not sufficient for spectral matching?

Response: The participants generally reported very few glycopeptides based on CID-MS/MS spectral evidence. The number of reported glycopeptides based on CID spectra were significantly lower than HCD spectra (File B: HCD = 2,736, CID = 151), Supplementary Figure S2. This under-reporting of CID-MS/MS spectra (discussed in the Results section p9) can most likely be attributed to the fact that relatively few software solutions can handle (or indeed make good use of) this type of fragmentation data rather than poor spectral quality of the provided CID data. Notably, CID-MS/MS is known to inform predominantly on the glycan part of the glycopeptides as opposed to the more peptide-centric HCD and site-centric EThcD data, possibly providing another reason why only few teams report glycopeptides based on this fragmentation method in this study which does not address the glycan sequence and topology. Of the nine developers, only two software solutions used CID spectral evidence to complete their reports (Team 1 – IQ-GPA, only high-resolution CID and Team 8 GlycoPAT, both high- and low-resolution CID data). Interestingly, Team 1 and 8 both came out well in our performance ranking. It remains to be tested whether the inclusion of CID data is a critical feature for the performance of IQ-GPA and GlycoPAT. Thus, we prefer not to speculate on this potential benefit of CID for these two software solutions. To more systematically address whether CID benefits the glycopeptide search performance of Byonic, we have now conducted a series of controlled search engine-centric in-house searches in which we systematically varied the data input (fragmentation spectral types) while keeping the search settings and data filtering constant. The data from these additional analyses (see Supplementary Table S19c) demonstrated that low-resolution CID data does neither significantly benefit the Byonic search performance when used alone nor when used in concert with the other more efficient fragmentation modes (i.e. HCD and EThcD). Further, analysis of the performance profiles across the Byonic teams that did use CID and those that did not use CID did not provide any evidence to support that CID provides measurable benefits to the Byonic search of glycoproteomics data (Supplementary Table S19d). These points have briefly been discussed in the Results section (p24) in the revised manuscript.

9. I would appreciate the inclusion of more raw data comparisons, for example: MS/MS of a glycopeptide that was annotated the same across platforms and of one that was differently annotated across platforms. Hopefully, this would make it insightful how ions are handled across algorithms.

Response: This is a really good suggestion. We have now included several manually annotated MS/MS spectra of glycopeptides that were correctly or incorrectly interpreted by the teams (see new Supplementary Figure S5 and Supplementary Figure S6). The individual spectra included for those figures were carefully selected as representative examples to support and align with the conclusions of the study. In one of those figures (Supplementary Figure S6), we included an example of an HCD-MS/MS scan that most teams assigned correctly; a consensus glycopeptide that was correctly annotated by 16 of 22 teams. We have also in those figures focused on MS/MS spectra of glycopeptides that carry “difficult-to-assign” features such as NeuAc><NeuGc, multi-Fuc, Met oxidation and Cys carbamidomethylation. These challenging spectra were, as expected, inconsistently reported across teams. Please note that the main purpose of the Supplementary Figure S5 is to show that NeuGc glycopeptides are not detected and multi-Fuc glycopeptides are only rarely detected in human serum, and that teams reporting on these features often have misassigned the spectra. The two new figures have been mentioned in the Results section (pp15-16).

10. Some teams search for Na+ and K+ (and Ca2+?) adducts, which are currently grouped. Can the authors comment on whether searching for these adducts is helpful/harmful for the analysis?

Response: The two teams that included adducts (Na+ and K+, not Ca2+) in their search space were Team 2 and 12. Both of these teams reported adducted N-glycopeptides based on EThcD spectral evidence (File B). In attempts to test more systematically whether searching for adducts is beneficial for the analysis, we have now performed a series of in-house Byonic- based searches in which we, amongst many other variables, tested the relative performance of searches performed with and without Na+/K+ adducts for a limited subset of the glycan search space (inspired by Team 2 and 12) while keeping other search settings constant, see new Supplementary Table S19a for data. The outcomes from these additional analyses, which were generally communicated in the new Figure 4, showed that while the inclusion of adducts in a targeted manner in the glycan search space mildly benefits the search performance, greater performance gains were achieved with a literature-guided narrow glycan search space (which was also more frequently used by teams) thus the adduct search data have not been included directly in Figure 4 but can instead be found in Supplementary Table S19a (see permutation 12 for data).

11. Currently, I find the title somewhat misleading. The comparison is mostly between labs (rather than search strategies) and the glycopeptide data is serum N-/O-glycoproteomics data specifically.

Response: We thank the reviewer for this suggestion. We have rephrased the title to include “serum N- and O-glycopeptide data” and the running title to include “serum glycoproteomics data”. Given that we, in the revision phase, have included a comprehensive search engine-centric analysis leading to the identification of several performance- associated features of the search strategies employed by teams using Byonic (see above), we have decided to keep this part in the title of the revised manuscript. The

revised title is: “Community Evaluation of Glycoproteomics Informatics Solutions Reveals High-Performance Search Strategies of Serum N- and O-Glycopeptide Data”. The revised running title is: “High-performance search strategies of serum glycoproteomics data”.

12. While multifucosylated species were excluded as false positive, some multifucosylated compositions are still visible in network graph Fig S4 (for example, H4N4F2S1, H3N3F2S2).

Response: We appreciate the comment by the reviewer. We have now provided evidence for the rarely detected multi-Fuc glycofeature in the human serum sample investigated in this study (see Supplementary Figure S5 and response above). Albeit at low abundance, multi- Fuc glycopeptides were (unlike NeuGc glycopeptides) undoubtedly present in the sample, as supported by manual annotation of an HCD-MS/MS spectrum corresponding to a multi-Fuc glycopeptide, Supplementary Figure S5d. In further support of the presence of multi-Fuc glycopeptides, the reviewer correctly points out that two consensus O-glycopeptides and a single consensus N-glycopeptide carrying double Fuc were reported by the majority of the teams as shown in the network graphs in Supplementary Figure S7. However, the scarcity of the multi-Fuc and absence of NeuGc glycofeatures in our sample mean that we consider it valid to estimate that glycoPSMs reported with NeuGc and multi-Fuc features are putative false positives when assessed at the global scale for the purpose of scoring teams (N6 and O5).

13. While I understand that not every search engine can be included the authors do mention several that still appear to be valuable points of comparison, including pGlyco, MSFragger-Glyco and/or O-Pair Search. I think their exclusion should either be expanded upon in the discussion, or, with the data being available, a comparison could still be incorporated.

Response: See response above to Reviewer 1, Question 4.
Further, during the early phases of the study period, we reached out to all major developers that did not proactively sign up for the study including pGlyco2.0 (as mentioned by Reviewer 2). This developer team accepted to be part of the study but unfortunately, did not provide data generated with pGlyco2.0. We, therefore, argue that we have been inclusive in our approach and that all major search engines at the time the study was conducted were given the opportunity to participate. As now described in the Discussion (p33), this study is essentially a snapshot of the performance of the software solutions at the time the data analysis was performed and follow-up studies comparing the performance of the very latest glycoproteomics software upgrades and informatics solutions that are not included in this study are therefore warranted and may form part of future Human Glycoproteomics Initiative studies. MSFragger-glyco, O-pair and pGlyco were all named and the original papers cited in the in the Discussion of this manuscript (p33).

Reviewer #3:
Remarks to the Author:

Title: Community Evaluation of Glycoproteomics Informatics Solutions Reveals High- Performance Search Strategies of Glycopeptide Data

Summary:
As part of the Human Proteome Project – Human Glycoproteomics Initiative, Kawahara et al. report the results of a community effort to document and evaluate several glycoproteomic search algorithms and software platforms. Two LC-MS/MS data files of glycopeptides enriched from human serum were provided to 22 teams of either software developers or expert users, and teams reported both N- and O-glycopeptides with a common template to enable thorough analyses of outputs and metrics. This important study not only compares relative performance of analysis pipelines for glycopeptide identifications, but also identifies key settings, variables, and features of algorithms that impact glycoproteomic searches. The manuscript is information dense, but this is a good thing considering the number of metrics reported for glycopeptide data. The figures are well designed and organized to complement the data. The tables provided are particularly useful and will be among the most referenced part of the manuscript by readers, especially Table 3 that summarizes search setting and output features associated with the study. The Discussion is also insightful, both summarizing the results and putting this study in context with current work and future needs. The large spread in glycopeptide identifications with relatively little overlap is simultaneously surprising and concerning – which further underscores the need for studies like this and consensus data to be reported. This manuscript merits publication in Nature Methods after the following minor comments have been addressed.

Response: We are very pleased with the positive comments. We sincerely thank the reviewer for their highly constructive suggestions that we have addressed below.

1. An addition that would be helpful to underscore the conclusions of this manuscript would be a comparison of two different search strategies by the same team, rather than the inter-team comparisons done throughout. Using the lessons learned here, one could imagine a "high-coverage" search strategy and a "high-accuracy" search strategy that would vary in the parameters spelled out in Table 3. Having the same team perform two different searches using the same algorithm and the same data would show how the range of glycoproteomic identifications can vary even within the same user(s) if different metrics are used. This would provide a frame of reference for readers of how these decisions will affect their data. This could be done on a high quality glycoproteomics dataset that is already published to demonstrate how the lessons gleaned from this work are applicable beyond the two data files studied here.

Response: This is an excellent idea, and we thank the reviewer for this suggestion. We have, on the one hand, now expanded on comparisons made between all teams that used Byonic in attempts to identify

search engine-specific search settings impacting the performance of this software (see also response above), and on the other hand, also performed a series of systematic in-house searches using Byonic on the shared data files to unpick performance- associated variables for Byonic. We considered it out of the scope of this study to perform these additional searches on new glycoproteomics data as the manuscript is already information dense in the current form (as acknowledged by this reviewer). Importantly, these additional analyses have led to improved Byonic-focused search strategies for N- and O- glycopeptides that the community can directly benefit from. We considered the reviewer's excellent suggestion of devising a "high coverage" and "high accuracy" search strategy and have additionally added a "balanced" search strategy which attempts to find a suitable compromise between specificity and sensitivity in a single search. We have included these data as a new Figure 4 (and in Supplementary Table S19) and discussed the findings from these additional analyses in the Results section (pp22-24) and Discussion (p31) in the revised manuscript.

2. Performance tests N6 and O5 that evaluate NeuGc and multi-Fuc-glycopeptides are described to measure the average of non-NeuGc and non-Fuc≥2 containing glyco-PSMs. I understand this is done because a measure of 1 is best (i.e., no NeuGc or Fuc≥2 glyco- PSMs) and can be used in the overall average score calculations, but the naming of the tests is counterintuitive. Several times I found myself having to double or triple check my understanding that a high NeuGc/Multi-Fuc score actually meant FEW NeuGc/Multi-Fuc hits. I recommend the authors either rename these tests or more explicitly describe this inverse relationship in the text.

Response: We have clarified and rephrased the N6 and O5 performance tests directly in the Table 2 (where readers are most likely to see this) in the revised manuscript. Note that we have also expanded on the text relating to the absence of NeuGc and rarity of multi-Fuc in the context of N6 and O5 (e.g. Results pp15-16) and included a new figure to support this Supplementary Figure S5, thus making it likely that readers now better understand this performance test.

3. The y-axis label is obstructing numbers on the y-axis of Supplemental Figure S3i.

Response: We have fixed this mistake (similar issue with Supplementary Figure S3b). Thank you for pointing this out.

4. Supplementary S5c, where the score is calculated as an average of the sensitivity and specificity, the scores do not seem to match. For example, Team 1 has a sensitivity of 50 and a specificity of 100. How then is the average result in a score of 50? This trend holds for all scores in this figure. Could the authors clarify?

Response: The score was in fact calculated by multiplying (not averaging) the sensitivity and specificity scores from this performance test. We apologise for this mistake. We have corrected the description

directly in the Table 2 and Extended Methods in the revised manuscript. We also inform the reviewer that we have updated Supplementary Figure S8 (former S5) to now show the 12 MS/MS spectra from File A-B that were originally used for the performance testing (N1). No performance scores were changed as a result of this update, some of the original spectra were simply left out in the figure by mistake in the original submission. We have also improved the annotation by labelling each spectrum with colour coded asterisks, which we hope the readers will find useful.

5. The authors do not discuss quantification much, other than to use spectral counting for some of the performance tests. In the Discussion, can the authors put the future of glycoproteomics quantitative strategies into context with the data they present here? If we have this much trouble identifying the same things, how can we imagine reliable quantification?

Response: We agree and have now expanded on the need to assess the ability of software to perform glycopeptide quantitation in future comparison studies in the Discussion section (p33) in the revised manuscript.

Editorial comment:
Remarks to the Author:
We have decided to invite you to revise your manuscript as you have outlined, before we reach a final decision on publication. While we understand that some of the analysis cannot be performed since the datasets are now public, we would like to request that you discuss more clearly the fact that there are newer tools available that aren't compared in this analysis and that this is a limitation of the study.

Response: We thank the editor for this comment. To address this point, we have now expanded the discussion concerning the limitations of the study in the Discussion section (p33): The revised paragraph is as follows:

“Most software currently available for glycoproteomics data analysis participated in this study. However, several glycopeptide search engines e.g. pGlyco52, MSFragger-Glyco53, and O-Pair Search54 were unfortunately not represented due to LC-MS/MS data incompatibility or due to their development after the study period. Thus, this study is essentially a snapshot of the performance of software available at the time the data analysis was performed. Highlighting the rapid progress in glycoproteome informatics, most of the software solutions participating in this study have been improved and new versions released after the evaluation period. For example, GPQuest v2.0, GlycoPAT v1.0 and Protein Prospector v5.20.23 tested herein have been superseded by more recent versions i.e. GPQuest v2.1, GlycoPAT v2.0 and Protein Prospector v.6.2.2. Thus, a limitation of this study is that newer tools are available at the time of publication that were not compared in our analysis. Follow- up studies comparing the performance of these latest glycoproteomics software upgrades and informatics

solutions not included in this study are therefore warranted. Beyond testing the ability of participants to identify the peptide and glycan components of glycopeptides from glycoproteomics data, such future comparative studies should ideally also test the ability to accurately quantify (relative, absolute) and report on modification sites of identified glycopeptides and could explore other relevant parameters not addressed herein including the use of alternative proteases, TMT-labelling, and stepped-HCD-MS/MS data amongst other experimental conditions gaining popularity in glycoproteomics."

Overview of major changes introduced in the revision phase:
- Added co-author (Anastasia Chernykh) for considerable work relevant to the revision of the manuscript.
- Validated the use of literature for the performance tests (N2-N3, O1-O2) by performing quantitative comparison of the observed site-specific glycoprofile of four serum glycoproteins to the literature (Supplementary Figure S4).
- Validated the scoring and ranking of the team by devising an independent scoring of teams based on the observed site-specific N-glycan distribution of four serum glycoproteins (Supplementary Figure S10, Supplementary Table S17).
- Performed comprehensive search engine-centric analysis of the 11 teams using Byonic and determined the impact of key search variables (search settings and data input) on the Byonic performance (Figure 4, Supplementary Table S19)
- Have kept but updated Figure 1-3 and Table 1-3 to match changes elsewhere in the revised manuscript.
- Have added method details of the additional analyses to the SI Method section and Online Methods.
- Have shortened the manuscript including the Abstract (now 210 words) and Introduction where possible.
- Have modified the Title and Running title and added N- and O-glycosylation to the Keywords.
- Have repositioned main figures and tables in manuscript to a more appropriate location.
- Have included multiple new references in the Extended Method section and removed five references (the original Ref 8-12 were substituted with a recent review reference, originally Ref 13) from the main manuscript to lower the total citation count in accordance with guidelines.
- Have updated the Editorial Policy Checklist and Reporting Summary as requested
- Have provided evidence of absence of NeuGc and only low levels of detectable multi- Fuc glycan features (Supplementary Figure S5)
- Have provided examples of our manually annotated MS/MS spectra that were correctly and incorrectly identified by teams (Supplementary Figure S6).

Overview of new and changed/updated graphical elements included in the revision:

	Graphical element	Brief description
New figures/tables		
	Figure 4	Results from search engine-centric analysis of the 11 teams using Byonic and the impact of key search variables (search settings and data input) on the Byonic performance
	Supplementary Figure S4	Comparison of site-specific glycoprofile of four serum glycoproteins in the investigated serum sample and glycoprofiles of those proteins reported by literature
	Supplementary Figure S5	Evidence of the absence of NeuGc and low levels of detectable multi-Fuc glycan features in the investigated serum sample
	Supplementary Figure S6	Examples of annotated MS/MS spectra correctly and incorrectly identified by teams
	Supplementary Figure S10	Independent glycoprotein-centric scoring to validate the team scoring and ranking
	Supplementary Table S17	Data supporting the independent glycoprotein-centric scoring to validate team scoring and ranking
	Supplementary Table S19	Data supporting the search engine-centric analysis of Byonic performance
Changed/updated figures/tables		
	Figure 1-3	Minor updates to match changes elsewhere in the revised manuscript
	Supplementary Figure S3	Minor adjustment of Y-axis legend
	Supplementary Figure S8	Inclusion of three annotated MS/MS spectra from the synthetic *N*-glycopeptide identified in File A and labelling of key fragment type of spectra using colour coded asterisks
	Supplementary Table S3	Updated correction of the MS/MS scan numbers from Team 9 as requested by these participants
	Supplementary Table S5, S7 and S12	Updated calculation of normalised specificity and sensitivity of synthetic *N*-glycopeptide and protein source score for global sensitivity and specificity scores (Supplementary Table S16)
	Supplementary Table S16	Summary of the global sensitivity and specificity scores for *N*- and *O*-glycopeptides

Decision Letter, second revision:

26th Jul 2021

Dear Morten,

Thank you for submitting your revised manuscript "Community Evaluation of Glycoproteomics Informatics Solutions Reveals High-Performance Search Strategies of Serum <i>N</i>- and <i>O</i>-Glycopeptide Data" (NMETH-AS45546A). It has now been seen by the original referees and their comments are below. The reviewers find that the paper has improved in revision, and therefore we'll be happy in principle to publish it in Nature Methods, pending minor revisions to satisfy the referees' final requests and to comply with our editorial and formatting guidelines.

Please note, we are overruling Reviewer #1's concerns.

We are now performing detailed checks on your paper and will send you a checklist detailing our editorial and formatting requirements in about a week. Please do not upload the final materials and make any revisions until you receive this additional information from us.

TRANSPARENT PEER REVIEW
Nature Methods offers a transparent peer review option for new original research manuscripts submitted from 17th February 2021. We encourage increased transparency in peer review by publishing the reviewer comments, author rebuttal letters and editorial decision letters if the authors agree. Such peer review material is made available as a supplementary peer review file. Please state in the cover letter 'I wish to participate in transparent peer review' if you want to opt in, or 'I do not wish to participate in transparent peer review' if you don't. Failure to state your preference will result in delays in accepting your manuscript for publication.
Please note: we allow redactions to authors' rebuttal and reviewer comments in the interest of confidentiality. If you are concerned about the release of confidential data, please let us know specifically what information you would like to have removed. Please note that we cannot incorporate redactions for any other reasons. Reviewer names will be published in the peer review files if the reviewer signed the comments to authors, or if reviewers explicitly agree to release their name. For more information, please refer to our FAQ page.

Thank you again for your interest in Nature Methods Please do not hesitate to contact me if you have any questions.

Sincerely,
Arunima

Arunima Singh, Ph.D.
Senior Editor
Nature Methods

ORCID
IMPORTANT: Non-corresponding authors do not have to link their ORCIDs but are encouraged to do

so. Please note that it will not be possible to add/modify ORCIDs at proof. Thus, please let your co-authors know that if they wish to have their ORCID added to the paper they must follow the procedure described in the following link prior to acceptance:
https://www.springernature.com/gp/researchers/orcid/orcid-for-nature-research

Reviewer #1 (Remarks to the Author):

The revised version strengthens the weakness of this manuscript.

Point 1. The revised version can provide a better understanding on the diversity of software for the readers.

Point 2. This comment was specific for O-glycopeptides. In table 3, the performance tests showed that this study considered the Unique O-glycopeptides (unique peptide sequence and O-glycan composition). In my opinion, the definition of "unique" varies from person to person. I would suggest adding site-localization information was not considered in table 3 to reduce the ambiguity although readers can obtain this information at the result section.

Point 3. I agree with the changes.

Point 4. I do not fully agree with the authors regarding the statement "we strongly believe that including new developer teams at this late stage in the process would not be appropriate." Both MSFragger-glyco and O-pair raised a lot of attention for glycobiologists and non-glycobiologists last year due to their high performance and search speed. The scientific community is eager to know the opinions about the new and long-existing tools from the experts as authors. The most-used tool, Byonic, in this study has been developed for more than 10 years. Therefore, the developers shall have more experience and better understanding on the fragmentation patterns and scoring of glycopeptides than the new software developers. In addition, there are no significant changes on glycopeptide assignment from Byonic version 2.14 to the latest version. Therefore, the occurrence of a big change on glycopeptide assignment is unlikely when the latest Byonic version is applied. In my opinion, the authors do not need to redo the whole analysis but shall at least run file B with both tools as side-by-side comparison and provide comments on the long-existing tools and the latest ones in the discussion part.

Reviewer #2 (Remarks to the Author):

Kawahara R. and co-authors have performed an extensive revision on basis of the reviewer comments. I find the manuscript much improved by the inclusion of the Byonic search parameter comparison, as well as by the various additional investigations of the raw mass spectrometric data. These changes have been incorporated well into the main manuscript and the main message remains clear and timely.

I see no reason to recommend against publication of the manuscript.

Reviewer #3 (Remarks to the Author):

The authors did a thorough job responding to our comments and those from the other reviewers. Especially valuable are the addition of the search engine-centric analysis of the teams using Byonic (as summarized in Figure 4); the comparison of the "accuracy", "coverage", and "balanced" search settings relative to default; the comparison to "ground truth" common N-glycoproteins to support their scoring metrics and evaluations; and the manually annotated spectra that were correctly and incorrectly identified by various teams. We thank the authors for their careful attention to each comment made. Our concerns have been addressed, and this manuscript now looks suitable for publication.

Author Rebuttal, Second Revision:

Response to reviewer's final comments

Dear Editor,

We are delighted to hear that the three reviewers find that the revisions have strengthened our manuscript. We here (in red) respond to the final comments raised by Reviewer #1. For clarity, we have below copied in below the decision letter we received from the journal editors.

Our ref: NMETH-AS45546A

26th Jul 2021

Dear Morten,

Thank you for submitting your revised manuscript "Community Evaluation of Glycoproteomics Informatics Solutions Reveals High-Performance Search Strategies of Serum N- and O-Glycopeptide Data" (NMETH-AS45546A). It has now been seen by the original referees and their comments are below. The reviewers find that the paper has improved in revision, and therefore we'll be happy in principle to publish it in Nature Methods, pending minor revisions to satisfy the referees' final requests and to comply with our editorial and formatting guidelines.

Please note, we are overruling Reviewer #1's concerns.

We have factored this ruling into our response to Reviewer #1 below.

We are now performing detailed checks on your paper and will send you a checklist detailing our editorial and formatting requirements in about a week. Please do not upload the final materials and make any revisions until you receive this additional information from us.

TRANSPARENT PEER REVIEW
Nature Methods offers a transparent peer review option for new original research manuscripts submitted from 17th February 2021. We encourage increased transparency in peer review by publishing the reviewer comments, author rebuttal letters and editorial decision letters if the authors agree. Such peer review material is made available as a supplementary peer review file. Please state in the cover letter 'I wish to participate in transparent peer review' if you want to opt in, or 'I do not wish to participate in transparent peer review' if you don't.

We wish to participate in transparent peer review.

Failure to state your preference will result in delays in accepting your manuscript for publication. Please note: we allow redactions to authors' rebuttal and reviewer comments in the interest of confidentiality. If you are concerned about the release of confidential data, please let us know specifically what information you would like to have removed. Please note that we cannot incorporate redactions for any other reasons. Reviewer names will be published in the peer review files if the reviewer signed the comments to authors, or if reviewers explicitly agree to release their name. For more information, please refer to our FAQ page.

Thank you again for your interest in Nature Methods Please do not hesitate to contact me if you have any questions.

Sincerely,
Arunima

Arunima Singh, Ph.D.
Senior Editor
Nature Methods

ORCID
IMPORTANT: Non-corresponding authors do not have to link their ORCIDs but are encouraged to do so. Please note that it will not be possible to add/modify ORCIDs at proof. Thus, please let your co-authors know that if they wish to have their ORCID added to the paper they must follow the procedure described in the following link prior to acceptance:
https://www.springernature.com/gp/researchers/orcid/orcid-for-nature-research

We have shared this information with our many coauthors.

Reviewer #1 (Remarks to the Author):

The revised version strengthens the weakness of this manuscript.

We are delighted to hear that Reviewer #1 finds that the revision has strengthened our manuscript.

Point 1. The revised version can provide a better understanding on the diversity of software for the readers.

We argue that we have already discussed the diversity of current software in the introduction of the manuscript e.g. (p7): "Glycoproteomics has experienced the development of diverse commercial and academic software showing promise for precise annotation and identification of glycopeptides from MS/MS data20, 21. While some of these tools are already well-established and widely applied in glycoproteomics22, the relative performance of software available to the community remain untested leaving a critical knowledge gap that hinders rapid progress in the field."

20. Hu, H., Khatri, K. & Zaia, J. Algorithms and design strategies towards automated glycoproteomics analysis. Mass Spectrom Rev 36, 475-498 (2017).
21. Abrahams, J.L. et al. Recent advances in glycoinformatic platforms for glycomics and glycoproteomics. Curr Opin Struct Biol 62, 56-69 (2020).
22. Cao, W. et al. Recent advances in software tools for more generic and precise intact glycopeptide analysis. Mol Cell Proteomics (2020).

However, we note that a new software for glycopeptide analysis (StrucGP) was recently published in a quality journal and have added this promising tool in the following sentence in the revised manuscript (p22): "Most software currently available for glycoproteomics data analysis participated in this study. However, several glycopeptide search engines e.g. pGlyco52, MSFragger-Glyco53, O-Pair Search54 and StructGP55 were unfortunately not represented due to LC-MS/MS data incompatibility or due to their development after the study period".

52. Liu, M.Q. et al. pGlyco 2.0 enables precision N-glycoproteomics with comprehensive quality control and one-step mass spectrometry for intact glycopeptide identification. Nat Commun 8, 438 (2017).
53. Polasky, D.A., Yu, F., Teo, G.C. & Nesvizhskii, A.I. Fast and comprehensive N- and O-glycoproteomics analysis with MSFragger-Glyco. Nat Methods 17, 1125-1132 (2020).
54. Lu, L., Riley, N.M., Shortreed, M.R., Bertozzi, C.R. & Smith, L.M. O-Pair Search with MetaMorpheus for O-glycopeptide characterization. Nat Methods 17, 1133-1138 (2020).
55. Shen, J. et al. StrucGP: de novo structural sequencing of site-specific N-glycan on glycoproteins using a modularization strategy. Nat Methods 18, 921-929 (2021).

Point 2. This comment was specific for O-glycopeptides. In table 3, the performance tests showed that this study considered the Unique O-glycopeptides (unique peptide sequence and O-glycan composition). In my opinion, the definition of “unique” varies from person to person. I would suggest adding site-localization information was not considered in table 3 to reduce the ambiguity although readers can obtain this information at the result section.

We have now added the suggested text in the relevant table (new Table 1).

Point 3. I agree with the changes.
Terrific.

Point 4. I do not fully agree with the authors regarding the statement “we strongly believe that including new developer teams at this late stage in the process would not be appropriate.” Both MSFragger-glyco and O-pair raised a lot of attention for glycobiologists and non-glycobiologists last year due to their high performance and search speed. The scientific community is eager to know the opinions about the new and long-existing tools from the experts as authors. The most-used tool, Byonic, in this study has been developed for more than 10 years. Therefore, the developers shall have more experience and better understanding on the fragmentation patterns and scoring of glycopeptides than the new software developers. In addition, there are no significant changes on glycopeptide assignment from Byonic version 2.14 to the latest version. Therefore, the occurrence of a big change on glycopeptide assignment is unlikely when the latest Byonic version is applied. In my opinion, the authors do not need to redo the whole analysis but shall at least run file B with both tools as side-by-side comparison and provide comments on the long-existing tools and the latest ones in the discussion part.

We find that the considerable potential of the more recent tools was already accurately discussed in the Introduction (p7) (“While informatics challenges undoubtedly still exist in glycoproteomics, our study highlights that several computational tools, some already demonstrating high performance, others considerable potential, are available to the community”) and in the Discussion e.g. (p20) “Excitingly, these three academic tools were recently developed (< 5 years ago), and thus, hold a considerable potential in the field.”. The existence of the more established tools was also mentioned in the Discussion (p19): “Protein Prospector51 and Byonic33, developed 10-20 years ago, have pioneered the glycopeptide informatics field and are search engines already commonly used in glycoproteomics8, 31, 33.”. To this end and to align with the editorial ruling (see above), we have therefore left the manuscript unchanged.

Reviewer #2 (Remarks to the Author):

Kawahara R. and co-authors have performed an extensive revision on basis of the reviewer comments. I find the manuscript much improved by the inclusion of the Byonic search parameter comparison, as well as by the various additional investigations of the raw mass spectrometric data. These changes have been incorporated well into the main manuscript and the main message remains clear and timely.

I see no reason to recommend against publication of the manuscript.

We are delighted to hear that Reviewer #2 finds our work suitable for publication.

Reviewer #3 (Remarks to the Author):

The authors did a thorough job responding to our comments and those from the other reviewers. Especially valuable are the addition of the search engine-centric analysis of the teams using Byonic (as summarized in Figure 4); the comparison of the “accuracy”, “coverage”, and “balanced” search settings relative to default; the comparison to “ground truth” common N-glycoproteins to support their scoring metrics and evaluations; and the manually annotated spectra that were correctly and incorrectly identified by various teams. We thank the authors for their careful attention to each comment made. Our concerns have been addressed, and this manuscript now looks suitable for publication.

We are delighted to hear that Reviewer #3 finds our work suitable for publication.

Final Decision Letter:

22nd Sep 2021

Dear Morten,

I am pleased to inform you that your Analysis, "Community Evaluation of Glycoproteomics Informatics Solutions Reveals High-Performance Search Strategies of Serum <i>N</i>- and <i>O</i>-Glycopeptide Data", has now been accepted for publication in Nature Methods. Your paper is tentatively scheduled for publication in our November print issue, and will be published online prior to that. The received and accepted dates will be March 19, 2021 and September 22, 2021. This note is intended to let you know what to expect from us over the next month or so, and to let you know where to address any further questions.

Acceptance is conditional on the data in the manuscript not being published elsewhere, or announced in the print or electronic media, until the embargo/publication date. These restrictions are not intended to deter you from presenting your data at academic meetings and conferences, but any enquiries from the media about papers not yet scheduled for publication should be referred to us.

In approximately 10 business days you will receive an email with a link to choose the appropriate publishing options for your paper and our Author Services team will be in touch regarding any additional information that may be required.

Please note that <i>Nature Methods</i> is a Transformative Journal (TJ). Authors may publish their research with us through the traditional subscription access route or make their paper immediately open access through payment of an article-processing charge (APC). Authors will not be required to make a final decision about access to their article until it has been accepted. Find out more about Transformative Journals

Authors may need to take specific actions to achieve compliance with funder and institutional open access mandates. For submissions from January 2021, if your research is supported by a funder that requires immediate open access (e.g. according to Plan S principles) then you should select the gold OA route, and we will direct you to the compliant route where possible. For authors selecting the subscription publication route our standard licensing terms will need to be accepted, including our self-archiving policies. Those standard licensing terms will supersede any other terms that the author or any third party may assert apply to any version of the manuscript.

You will not receive your proofs until the publishing agreement has been received through our system.

If you have any questions about our publishing options, costs, Open Access requirements, or our legal forms, please contact ASJournals@springernature.com

Your paper will now be copyedited to ensure that it conforms to Nature Methods style. Once proofs are generated, they will be sent to you electronically and you will be asked to send a corrected version within 24 hours. It is extremely important that you let us know now whether you will be difficult to contact over the next month. If this is the case, we ask that you send us the contact information (email, phone and fax) of someone who will be able to check the proofs and deal with any last-minute problems.

If, when you receive your proof, you cannot meet the deadline, please inform us at rjsproduction@springernature.com immediately.

Once your manuscript is typeset and you have completed the appropriate grant of rights, you will receive a link to your electronic proof via email with a request to make any corrections within 48 hours. If, when you receive your proof, you cannot meet this deadline, please inform us at rjsproduction@springernature.com immediately.

Once your paper has been scheduled for online publication, the Nature press office will be in touch to confirm the details.

If you have posted a preprint on any preprint server, please ensure that the preprint details are

updated with a publication reference, including the DOI and a URL to the published version of the article on the journal website.

Once your paper has been scheduled for online publication, the Nature press office will be in touch to confirm the details.

Content is published online weekly on Mondays and Thursdays, and the embargo is set at 16:00 London time (GMT)/11:00 am US Eastern time (EST) on the day of publication. If you need to know the exact publication date or when the news embargo will be lifted, please contact our press office after you have submitted your proof corrections. Now is the time to inform your Public Relations or Press Office about your paper, as they might be interested in promoting its publication. This will allow them time to prepare an accurate and satisfactory press release. Include your manuscript tracking number NMETH-AS45546B and the name of the journal, which they will need when they contact our office.

About one week before your paper is published online, we shall be distributing a press release to news organizations worldwide, which may include details of your work. We are happy for your institution or funding agency to prepare its own press release, but it must mention the embargo date and Nature Methods. Our Press Office will contact you closer to the time of publication, but if you or your Press Office have any inquiries in the meantime, please contact press@nature.com.

To assist our authors in disseminating their research to the broader community, our SharedIt initiative provides you with a unique shareable link that will allow anyone (with or without a subscription) to read the published article. Recipients of the link with a subscription will also be able to download and print the PDF.
As soon as your article is published, you will receive an automated email with your shareable link.

You can now use a single sign-on for all your accounts, view the status of all your manuscript submissions and reviews, access usage statistics for your published articles and download a record of your refereeing activity for the Nature journals.

Nature Research journals encourage authors to share their step-by-step experimental protocols on a protocol sharing platform of their choice. Nature Research's Protocol Exchange is a free-to-use and open resource for protocols; protocols deposited in Protocol Exchange are citable and can be linked from the published article. More details can found at www.nature.com/protocolexchange/about.

Please note that you and any of your coauthors will be able to order reprints and single copies of the issue containing your article through Nature Research Group's reprint website, which is located at http://www.nature.com/reprints/author-reprints.html. If there are any questions about reprints please send an email to author-reprints@nature.com and someone will assist you.

Please feel free to contact me if you have questions about any of these points.

Best regards,
Arunima

Arunima Singh, Ph.D.
Senior Editor
Nature Methods

** Visit the Springer Nature Editorial and Publishing website at www.springernature.com/editorial-and-publishing-jobs for more information about our career opportunities. If you have any questions please click here.**
